# Clinical outcomes of mitochondrial‐enhancing nutraceutical supplementation in psychiatric disorders: A systematic review

**DOI:** 10.1002/gps3.70023

**Published:** 2026-06-01

**Authors:** Juan Tortajada, Bengisu Kevser Bulduk, Belén Alfonso‐Landete, Paula Alcaide‐Barriga, Bernat Ballvé‐Gelonch, Glòria Garrabou, Elisabet Vilella, Yolanda Alonso, Alba Valiente‐Pallejà, Lourdes Martorell

**Affiliations:** ^1^ Hospital Universitari Institut Pere Mata (HUIPM) Reus Catalonia Spain; ^2^ Institut de Recerca Biomèdica Catalunya Sud (IRB CatSud) Reus Catalonia Spain; ^3^ Universitat Rovira i Virgili (URV) Reus Catalonia Spain; ^4^ Centro de Investigación Biomédica en Red en Salud Mental CIBERSAM‐Instituto de Salud Carlos III Madrid Spain; ^5^ Inherited Metabolic Diseases and Muscular Disorders Research Group Department of Internal Medicine‐Hospital Clínic of Barcelona (HCB) Faculty of Medicine and Health Science University of Barcelona (UB) Barcelona Catalonia Spain; ^6^ Institut d'Investigacions Biomèdiques August Pi i Sunyer (IDIBAPS) Barcelona Catalonia Spain; ^7^ Centro de Investigación Biomédica en Red de Enfermedades Raras CIBERER‐Instituto de Salud Carlos III Madrid Spain

**Keywords:** dietary supplements, mental disorders, mitochondria, N‐acetylcysteine, vitamin D3

## Abstract

**Background:**

Nutraceutical supplementation targeting mitochondrial function has been proposed as a beneficial therapeutic strategy to improve physical and mental health in psychiatric patients.

**Aims:**

To summarise the results of studies evaluating nutraceutical supplementation targeting mitochondrial function in patients with psychiatric disorders.

**Methods:**

Following the Preferred Reporting Items for Systematic Reviews and Meta‐Analyses guidelines, we searched PubMed, Embase and Scopus databases from 1 January 2007 to 30 April 2024. Reports were included if they evaluated outcomes of nutraceutical supplementation in patients with psychiatric disorders or related conditions. Additionally, we performed a risk‐of‐bias analysis of the studies compatible with the RoB2 tool.

**Results:**

Of the 2061 records identified, 122 studies met the inclusion criteria, evaluating vitamin D3, N‐acetylcysteine, acetyl‐L‐carnitine, coenzyme Q10, alpha‐lipoic acid, magnesium, vitamin B6, vitamin B7, folic acid, vitamin B12, vitamin E, vitamin A, vitamin C and vitamin B3. The most studied nutraceuticals were vitamin D3 (27.05%) and N‐acetylcysteine (15.6%). Among randomised controlled clinical trials (RCTs), vitamin D3 was the most extensively investigated and accounted for the highest number of trials reporting improvements in clinical outcomes, although findings were heterogeneous. Notably, 14.8% of the studies evaluated combinations of three or more nutraceuticals. Dietary supplements were extensively evaluated for autism spectrum disorder (28 studies), schizophrenia spectrum disorder (27 studies), major depressive disorder or related depressive symptoms (22 studies), attention‐deficit hyperactivity disorder (9 studies) and bipolar spectrum disorder (6 studies). A substantial proportion of studies were not RCTs but open‐label single‐arm trials or case reports. Significant heterogeneity was observed in the nutraceutical components used, treatment duration and the outcomes assessed. Overall, the risk of bias was high, and the methodological quality was generally low.

**Conclusions:**

Promising findings in nutraceutical studies for psychiatric disorders face challenges, including small sample sizes, short follow‐up periods and a lack of treatment standardisation. Future research requires robust RCTs with standardised protocols and validated biomarkers of efficacy.

## Abstract (highlights)

**WHAT IS ALREADY KNOWN ON THIS TOPIC**

- Mitochondrial dysfunction has been implicated in psychiatric disorders such as schizophrenia, bipolar disorder, major depressive disorder and autism spectrum disorder. Nutraceutical supplementation, targeting mitochondrial function, has been proposed as a potential therapeutic strategy to address oxidative stress, neuroinflammation and neurotransmitter imbalances in these disorders.

**WHAT THIS STUDY ADDS**

- This systematic review summarises 122 studies that evaluated the effects of nutraceuticals, such as vitamin D3, N‐acetylcysteine and others, on psychiatric disorders. It includes a quality assessment of existing studies using the Risk of Bias 2 tool. Although most studies reported clinical or biological improvements, a high risk of bias and significant heterogeneity in treatment components, duration and outcomes were evident, calling for more robust trials.

**HOW THIS STUDY MIGHT AFFECT RESEARCH, PRACTICE OR POLICY**

- The findings suggest that nutraceuticals hold promise as adjunctive treatments in psychiatric care. However, the lack of standardisation and small sample sizes in current studies indicate the need for more rigorous research to guide clinical practice and inform health policy on the use of mitochondrial‐enhancing nutraceuticals in mental health treatment.

## INTRODUCTION

Psychiatric disorders are complex conditions influenced by a combination of environmental and genetic factors. Although the neurobiology of these disorders remains elusive, abnormalities in synaptic plasticity, neuronal connectivity and neuroinflammation have been implicated.^1^, ^2^ Mitochondrial dysfunction has been reported in several psychiatric disorders, including schizophrenia (SCZ),^3^ bipolar disorder (BD),^4^ major depressive disorder (MDD)^5^ and autism spectrum disorder (ASD).^6^ This association is not surprising given that the brain, despite representing only 2% of body weight, consumes up to 20% of the body's total energy, which is primarily produced in the mitochondria.^7^

Essential for neuronal activity, growth, development and plasticity, mitochondria play a central role in brain processes that require elevated energy levels. As key signalling hubs, mitochondria regulate cellular processes crucial for cell differentiation, proliferation, apoptosis and the immune response.^8^ These membrane‐bound organelles are responsible for generating most of the cell's chemical energy, primarily in the form of adenosine triphosphate (ATP), through the oxidative phosphorylation system (OXPHOS) located on the inner mitochondrial membrane. The OXPHOS system involves the oxidoreductase Complexes I‐IV of the electron transport chain and the ATP synthase enzyme of Complex V. This system is influenced by several cofactors, vitamins and minerals, including acetyl‐L‐carnitine (ALCAR), coenzyme Q10 (CoQ10), alpha‐lipoic acid (ALA), magnesium (Mg), vitamin B1 (thiamine), vitamin B2 (riboflavin), vitamin B3 (niacin), vitamin B5 (pantothenic acid), vitamin B6 (pyridoxine), vitamin B7 (biotin), vitamin B9 (folic acid [FA]), vitamin B12 (cobalamin), vitamin E (tocopherol), N‐acetylcysteine (NAC), vitamin A (retinol), vitamin C (ascorbic acid) and vitamin D3 (cholecalciferol). Detailed information on the composition, sources, functions and other relevant details can be found in Supporting Information S1: Appendix S1, including figure S1, which summarises the effect of nutraceutical supplements on key biological processes in mitochondria.

Nutraceutical supplementation has been investigated in psychiatric disorders due to its potential to address mitochondrial dysfunction, oxidative stress, inflammation and neurotransmitter imbalances. The primary objective of this systematic review is to summarise and present the key findings of studies that have used nutraceutical supplementation to affect the mitochondrial function in individuals with psychiatric disorders and related conditions.

## MATERIAL AND METHODS

### Search strategy and selection criteria

**FIGURE 1:**
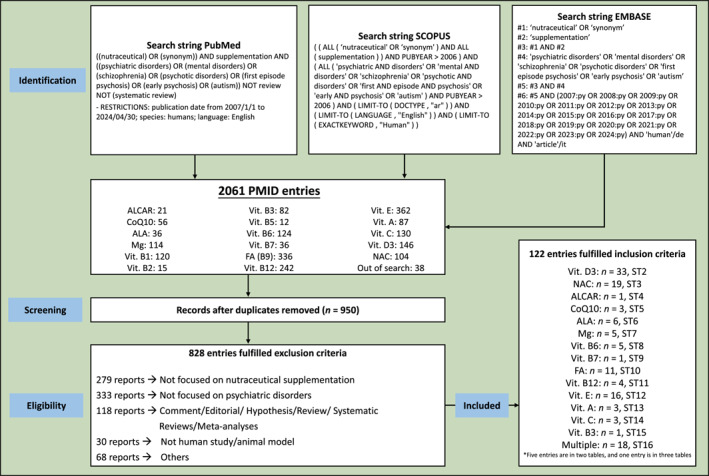
PRISMA flow diagram illustrating the study selection process for the systematic review. Flow diagram illustrating the study selection process for the systematic review. Records were identified through searches in PubMed, Scopus and Embase using predefined search strategies with filters for publication date (2007–2024), human studies and English language. A total of 2061 records were retrieved, of which 950 remained after duplicate removal. Following screening, 122 studies met inclusion criteria and were categorised according to nutraceutical type. The distribution of included studies across ST is shown for each compound. ALA, alpha‐lipoic acid; ALCAR, acetyl‐L‐carnitine; CoQ10, coenzyme Q10; FA, folic acid; Mg, magnesium; NAC, N‐acetylcysteine; PMID, PubMed identifier; PRISMA, Preferred Reporting Items for Systematic Reviews and Meta‐analyses; ST, Supplementary Table; Vit, vitamin.

We searched PubMed, Embase and Scopus for English‐language articles published between 1 January 2007 and 30 April 2024. Depending on the database, different searches were performed by combining keywords such as nutraceutical and its synonyms, supplementation, psychiatric disorders, mental disorders, SCZ, psychotic disorders, first‐episode psychosis, early psychosis and autism, according to the Preferred Reporting Items for Systematic Reviews and Meta‐Analyses (PRISMA) guidelines.^9^ Specific search terms used in each database are shown in figure 1.

Titles and abstracts retrieved were screened, and empirical studies that met the eligibility criteria were selected. The search strategy, data collection, extraction and assessment were performed independently by three authors (Belén Alfonso‐Landete, Juan Tortajada and Bengisu Kevser Bulduk). In cases of disagreement, a fourth author (Lourdes Martorell) facilitated consensus. The abstraction and summary of the main study results were performed independently by one of the three main authors and checked by another.

### Inclusion and exclusion criteria

Studies were included if they reported on human outcomes of nutraceutical supplementation in psychiatric disorders and were written in English. Exclusion criteria included studies not focused on psychiatric disorders, nutraceutical supplementation, commentaries, editorials, hypotheses, reviews, meta‐analyses, animal model studies and others (mainly prevention studies and evaluations of supplements not included in our review). No other limitations were applied.

### Review process

The combined search identified 2061 potentially eligible studies. After 1111 duplicate records were removed, 950 records were screened for eligibility; 828 did not meet the criteria, leaving 122 reports. The remaining 122 reports that met the inclusion criteria were reviewed in detail and their references are provided in Appendix S1. Figure 1 shows the PRISMA flowchart detailing the stages of the systematic review, and table S1 shows the PRISMA reporting checklist.

### Data extraction

From eligible articles, we recorded the first author's name, PMID number, year of publication, number of patients and controls studied, age, sex, disease or condition, study group (supplement or placebo), supplement dose and treatment duration, measures assessed, outcomes and additional information.

### Assessment of risk of bias in included studies

Four review authors (Belén Alfonso‐Landete, Juan Tortajada, Bengisu Kevser Bulduk and Paula Alcaide‐Barriga) independently assessed the quality of the studies according to the Cochrane Handbook for Systematic Reviews of Interventions, using the Risk of Bias 2 (RoB 2) tool, the latest version of which was available in August 2019.^10^ This tool estimates the overall risk of bias (ROB) as low risk, some concerns or high risk based on five domains: bias arising from the randomisation process, bias due to deviations from intended interventions, bias due to missing outcome data, bias in measurement of the outcome and bias in selection of the reported outcome. Disagreements were resolved by consensus with the involvement of another group member. To visualise the results, we used robvis, a visualisation tool designed to visualise ROB assessments performed as part of a systematic review. This tool uses a colour‐coded ranking system, where green indicates low ROB, yellow indicates some concerns and red indicates high ROB.

## RESULTS

### Characteristics of the studies reviewed

Studies that met the inclusion criteria were categorised into 15 groups based on the specific nutraceutical supplement studied. Some reports were included manually after reading specific references of the included studies. Each study was assigned to a category if the supplement was used either alone or in combination with another nutraceutical supplement. Consequently, some reports were included in more than one category if they presented results from the use of two or more supplements. Studies that evaluated more than two supplements were classified as multiple. The 15 categories identified were as follows: vitamin D3, NAC, ALCAR, CoQ10, ALA, Mg, vitamin B6, vitamin B7, FA, vitamin B12, vitamin E, vitamin A, vitamin C, vitamin B3 and multiple. For each category, we provide a supplementary table with a comprehensive summary of pertinent data, including the number of patients and controls analysed, age, sex, disease or condition, study groups, nutraceutical and placebo information, treatment duration, outcomes assessed, results and additional information. Additionally, the results are presented by diagnosis (tables 1, 2, 3, 4) for the main psychiatric disorders examined: ASD, SCZ, MDD, ADHD and BD. The tables detail the positive and negative results for each specific outcome in each study. These tables are intended to facilitate interpretation and support the analysis presented in the following sections.

**TABLE 1:** Nutraceutical supplementation and outcome measures evaluated in patients with ASD

Results	Author, year	Study design	PMID	Study population		Nutraceutical intervention		Outcome measures			
				*n*	Age (y) in P/C	Component	Treatment time (m)	Clinical symptoms	Cognitive function	Biological markers	Oxidative stress
				P/C							
+	Lai et al. (2021)^11^	Cross‐sectional	33328600	138/0	4.2 (1.2)	Vit A	6	**SRS**	N/A	**Retinol/OXT,** anthropometric measures, **CD38 gene expression** in PBMC, **RARs**	N/A
+	Pesko et al. (2020)^12^	Case series	32071590	4/0	14–17	NAC	1	**ABC‐I**	N/A	N/A	N/A
−	Kerley et al. (2020)^13^	Post hoc analysis of RCT	30301427	18/17	8.5 (3.5)	Vit D3	5	N/A	N/A	25(OH)D	N/A
+	Infante et al. (2020)^14^	Case report	30545280	1/0	23	Vit D3, EPA, DHA	24	CGI‐S, **CGI‐I, CARS**	N/A	**25(OH)D, arachidonic acid, EPA, DHA, omega‐6 PUFA, omega‐3 PUFA**	N/A
−	Feng et al. (2020)^15^	Case report	31914053	1/0	2.5	Vit D3	6	CARS	N/A	25(OH)D	N/A
−	Debi Ann et al. (2020)^16^	Case report	33036783	2/0	4	Vit B6, Mg	2	CARS2‐ST, ATEC, BEARS, 6‐GSI	N/A	N/A	N/A
−	Mazahery et al. (2019)^17^	RCT	30607782	117/0	2.5–9	Vit D3, DHA	12	SRS‐2, SPM	N/A	25(OH)D, omega‐3 PUFA	N/A
+	Mousavinejad et al. (2018)^18^	Nonstandard randomised	29684771	90/90	3–12	CoQ10	3.3	**CARS, gastrointestinal problems**, s**leep disorders**, verbal communication, playing with friends	N/A	**CoQ10**	**MDA, TAS**, GR, GSSG, **GPx, SOD**
+	Guo et al. (2018)^19^	Open‐label	29122693	33/32	5.1 (1.1)	Vit A	6	ABC, **CARS**, GDS	N/A	**Retinol, 5‐hydroxytryptamine, mRNA expression levels of retinoic acid receptors, tryptophan hydroxylase 1**	N/A
+	Adams et al. (2018)^20^	RCT	29562612	67/50	3–58	Multiple nutraceuticals^a^ and additional treatments	12	ADOS, **CARS‐2, SAS‐Pro, PDD‐BI, ATEC, ABC, SRS, SSP, PGI‐2, 6‐GSI**, **VABS‐II**, grip strength	**RIAS**	Vit/mineral, PUFA, homocysteine, carnitine, digestive enzymes	N/A
−	Dean et al. (2017)^21^	RCT	27316706	102/0	3.1–9.9	NAC	6	SRS, CCC‐2, PGI‐I, CGI‐I, CGI‐S, RBS‐R, DBC‐P	N/A	N/A	N/A
+	Siscoe et al. (2017)^22^	Case report	28272116	1/0	8	FA	9	ABA, MOAS	N/A	N/A	N/A
+	Liu et al. (2017)^23^	Nonrandomised, single‐blind intervention study	28938872	64/0	1–8	Vit A	6	ABC, CARS, SRS	N/A	**Retinol, CD38, RORA mRNA level test**	N/A
+	Kałużna‐Czaplińska et al. (2017)^24^	Cross‐sectional	28608247	236/0	3–16	Vitamins B, Mg, PUFA	‐	N/A	N/A	**Tryptophan**, BMI, Specific gravity, pH, leucocytes, nitrite, protein, glucose, ketones, urobilinogen, bilirubin, erythrocytes	N/A
+	Feng et al. (2017)^25^	Open‐label	26783092	215/285	5 (1.1)	Vit D3	3	**ABC, CARS**	N/A	**25(OH)D**	N/A
+	Kerley et al. (2017)^26^	RCT	28626020	38/0	7.4 (13.6)	Vit D3	5	ABC, **DD‐CGAS**, SRS	N/A	**25(OH)D**, immunity markers, systemic inflammatory markers	N/A
+	Saad et al. (2016)^27^	Open‐label	25876214	122/100	5 (1.4)	Vit D3	3	**CARS**	N/A	25(OH)D	N/A
+	Wink et al. (2016)^28^	RCT	27103982	31/0	4–12	NAC	3	CGI‐I	N/A	N/A	**GSH**
+	Sun et al. (2016)^29^	Open‐label	27338456	66/0	4.5 (1.2)	FA	3	**ABC, ATEC, PEP‐3, CARS**	N/A	Vit B12, **FA, homocysteine**	**GSH, GSSG**
+	Jia et al. (2015)^30^	Case report	25511123	1/0	2.66	Vit D3	2	**ABC, CARS, CGI‐S**	N/A	**25(OH)D**	N/A
+	Ziats et al. (2015)^31^	Case report	25943046	1/0	4	L‐carnitine	4.5	N/A	N/A	**Trimethyllysine, gamma‐butyrobetaine, free carnitine**, total acylcarnitine	N/A
+	Marler et al. (2014)^32^	Case report	24815193	1/0	4	NAC	2	**Self‐injurious behaviour**	N/A	N/A	N/A
+	Ghanizadeh et al. (2013)^33^	RCT	23886027	40/0	3.5–16	NAC	2	**ABC**	N/A	N/A	N/A
+	Hardan et al. (2012)^34^	RCT	22342106	33/0	3.2–10.7	NAC	3	CGI, SRS, **ABC, RBS‐R**	N/A	N/A	N/A
+	Adams et al. (2011)^35^	RCT	22151477	141/44	10.6 (5.5)	Multiple nutraceuticals^a^	3	PDD‐BI, ATEC, SAS, **PGI‐R**	N/A	**Metabolic parameters**	N/A
+	Kałużna‐Czaplińska et al. (2011)^36^	Nonrandomised, pre‐post intervention study	21840465	20/10	4–7	Vit B2, vit B6, Mg	3	N/A	N/A	**Dicarboxylic acids, creatinine**	N/A
+	Kałużna‐Czaplińska et al. (2011)^37^	Nonrandomised, pre‐post intervention study	21530806	30/21	4–11	FA, vit B6, vit B12	3	N/A	N/A	**Urinary homocysteine**	N/A
+	Xia et al. (2011)^38^	Case report	21417812	1/0	9	DMG, vit B6, Mg	3	**ATEC**	N/A	N/A	N/A

*Note*: Significant (+) or no (−) improvement in any of the parameters assessed. Measures in bold indicate a significant improvement in the intervention for that measure. Age is presented as mean (SD) or range.

*Nutraceutical supplements*: ALCAR, acetyl‐L‐carnitine; CoQ10, coenzyme Q10; Cr, chromium; DHA, docosahexaenoic acid; DMG, dimethylglycine; EPA, eicosapentaenoic acid; FA, folic acid; I, iodine; Li, lithium; Mg, magnesium; Mn, manganese; Mo, molybdenum; NAC, N‐acetylcysteine; PUFA, polyunsaturated fatty acids; Se, selenium; S, sulphur; Vit, vitamin; Zn, zinc.

*Measures*: 25(OH)D, 25‐hydroxyvitamin D3; 6‐GSI, 6‐item Gastrointestinal Severity Index; ABA, applied behaviour analysis; ABC, Aberrant Behaviour Checklist; ADOS, Autism Diagnostic Observation Schedule; ATEC, Autism Treatment Evaluation Checklist; BEARS, BEARS sleep screening tool; BMI, body mass index; CARS, Childhood Autism Rating Scale; CARS2‐ST, Childhood Autism Rating Scale, Second Edition‐Standard Version; CCC‐2, Children's Communication Checklist‐Second Edition; CGI‐I, Clinical Global Impression‐Improvement scale; CGI‐S, Clinical Global Impression‐Severity scale; DBC‐P, Developmental Behaviour Checklist‐Primary Carer Version; DD‐CGAS, Developmental Disabilities‐Children's Global Assessment Scale; DHA, docosahexaenoic acid; EPA, eicosapentaenoic acid; FA, folic acid; GDS, Gesell Developmental Scale; GPx, glutathione peroxidase; GR, glutathione reductase; GSH, reduced glutathione; GSSG, oxidised glutathione; MDA, malondialdehyde; MOAS, Modified Overt Aggression Scale; OXT, oxytocin; PBMC, peripheral blood mononuclear cells; PDD‐BI, Pervasive Developmental Disorders‐Behaviour Inventory; PEP‐3, Psychoeducational Profile‐Third Edition; PGI‐2, Parent Global Impressions‐Revised‐2; PGI‐I, Parent Global Impression‐Improvement scale; PGI‐R, Parental Global Impressions‐Revised; PUFA, polyunsaturated fatty acids; RARs, retinoic acid receptors; RBS‐R, Repetitive Behaviour Scale–Revised; RIAS, Reynolds Intellectual Assessment Scales; RORA, retinoic acid‐related orphan receptor alpha; SAS, Simpson Angus Scale; SAS‐Pro, Severity of Autism Scale; SOD, superoxide dismutase; SPM, Sensory Processing Measures; SRS, Social Responsiveness Scale; SRS‐2, SRS‐Second Edition; SSP, Short Sensory Profile; TAS, total antioxidant status; TSH, thyroid stimulating hormone; VABS‐II, Vineland Adaptive Behaviour Scale‐Second edition; Vit, vitamin.

Abbreviations: ASD, autism spectrum disorder; C, controls; d, days; m, months; N/A, unassessed outcomes; P, patients; PMID, PubMed identifier; RCT, randomised controlled trial; SD, standard deviation; y, years.

^a^ Vit A, vit C, vit D3, vit E, vit B1, vit B2, vit B3, vit B5, vit B6, vit B12, ALCAR, FA, biotin, choline, inositol, mixed carotenoids, CoQ10, NAC, Ca, Cr, I, Li, Mg, Mn, Mo, Se, S and Zn.

**TABLE 2:** Nutraceutical supplementation and outcome measures evaluated in patients with SCZ or related psychotic disorders

Results	Author, year	Study design	PMID	Study population		Nutraceutical intervention		Outcome measures				
				*n*	Age (y) in P/C	Component	Treatment time (m)	Clinical symptoms	Cognitive function	Quality of life	Biological markers	Oxidative stress
				P/C								
−	Zhang et al. (2023)^39^	Nonrandomised, observational	37252140	44/0	28.3 (4.4)	Vit C	3	PANSS	N/A	N/A	BMI, WHR, T‐c, TG, HDL‐c, LDL‐c	N/A
−	De Lima et al. (2023)^40^	RCT	36584248	35/0	37.8 (8.0)	ALA	4	BPRS, extrapyramidal symptoms	TMT, block Corsi test, subtest digit span, animal fluency test, COWAT‐FAS, RAVLT, Stroop	N/A	BMI, inflammatory parameters, haematological parameters, TNF‐α, S100 PG, nitrite	MPO, TBARS
−	Neill et al. (2022)^41^	RCT	35857752	75/0	39.74	NAC	13	PANSS, CDS, SAFTEE	MCCB	MANSA, AQoL	N/A	N/A
+	Mishra et al. (2022)^42^	RCT	36069950	20/0	18–65	ALA	2	SAPS, **SANS**, UKU‐SERS	SCoRS		**BDNF**	GSH, MDA
−	Maguire et al. (2021)^43^	RCT	33347024	72/0	18–70	CoQ10	3, 6	Energy, depression, anxiety, negative symptoms, physical activity, functional status	CPT‐IP, SWM, WMS, processing speed, executive function, general cognitive function	QoL	CoQ10, lactate, blood pressure	N/A
−	Gaughran et al. (2021)^44^	RCT	34962559	149/0	18–65	Vit D3	6	PANSS, CDS, GAF	N/A	N/A	WC, BMI, HbA1c, T‐c, CRP, vit D	N/A
+	Ghaderi et al. (2019)^45^	RCT	30791895	60/0	44 (7)	Vit D3	3	**PANSS**	N/A	N/A	**25(OH)D, metabolic parameters**	N/A
+	Mullier et al. (2019)^46^	Pilot of RCT	31283822	20/74	25 (6)	NAC	6	N/A	N/A	N/A	**fMRI**	N/A
+	Yang et al. (2019)^47^	RCT	30712814	19/0	49.1 (10.4)	NAC	2	MMN, **ASSR**, negative symptoms	N/A	N/A	N/A	N/A
+	Allot et al. (2019)^48^	RCT	30771856	120/0	19.9 (2.7)	FA, vit B6, vit B12	3	PANSS, BPRS, GAF, SANS, CDSS, YMRS, CGI	Composite neurocognition (11 test battery: **Attention/vigilance**)	N/A	**Homocysteine**	N/A
+	Breier et al. (2018)^49^	RCT	29588126	60/0	16–30	NAC	13	**PANSS‐T**, PANSS‐P, **PANSS‐N**, PANSS‐G, CGI‐S, PSP	BACS	N/A	MRI	N/A
+	Conus et al. (2018)^50^	RCT	29462456	63/0	18–40	NAC	6	PANSS, GAF, SOFAS, UKU‐SERS	MCCB (9 of 10 subtests)		MRI	**GSH**_**BC**_ **, GSH**_**mPFC**_, CYS_Pl_, **GPx**_**BC**_
+	Sepehrmanesh et al. (2018)^51^	RCT	29126981	84/0	39.1 (2.1)	NAC	3	**PANSS‐T, PANSS‐P, PANSS‐N**, PANSS‐G	**MMSE**, **neuropsychological tests**	N/A	N/A	N/A
+	Itokawa et al. (2018)^52^	Open‐label	29064136	10/0	38–64	Vit B6	6	**PANSS**	N/A	N/A	N/A	**Pentosidine**
+	Roffman et al. (2018)^53^	RCT	28289280	55/0	45.5 (11.1)	FA	3	**PANSS‐T, PANSS‐N, PANSS‐G**, **SANS**, **CDSS**	MCCB	N/A	FA, **MRI**	N/A
+	Rapado‐Castro et al. (2017)^54^	Post hoc analysis of RCT	27894373	58/0	39.8 (12.3)	NAC	6	N/A	Attention, **working memory**, executive function	N/A	N/A	N/A
+	Sanders et al. (2017)^55^	Open‐label	29053478	10/0	18–60	ALA	4	**BPRS**	**TMT, block Corsi test, subtest digit span**, animal fluency test, COWAT‐FAS, RAVLT	N/A	BMI, ALT, AST, HbA1c, FA, vit B12, hs‐CRP, nitrite, **TBARS**, IL‐1β, IL‐4, interferon γ, IDO	GSH
+	Vidović et al. (2017)^56^	Open‐label	28009525	18/0	25–60	ALA	3	N/A	N/A	N/A	**Glucose**, HDL‐c, LDL‐c, T‐c, **TG**, **AST**, ALT, GGT, **BMI**, **WC**, body fat, **FLI**, **adiponectin**, MUFA, PUFA, n‐6 PUFA, n‐3 PUFA, n6/n3, leptin, O_2_^−^	MDA, TAC, SFA
−	Krivoy et al. (2017)^57^	RCT	29226809	47/0	41 (10.5)	Vit D3	2	PANSS, CDS	MoCA	N/A	25(OH)D, metabolic parameters	N/A
+	Monsivais et al. (2016)^58^	Case report	27059873	1/0	38	NAC	12	**BPRS**	**Neuropsychological testing not specified**	N/A	**MRI**	N/A
−	Vidović et al. (2014)^59^	Open‐label	25191766	18/38	25–60	ALA	1.5, 3	N/A	N/A	N/A	BMI, fat %, WC, WHR, SBP, DBP, glucose, T‐c, HDL‐c, LDL‐c, TG, uric acid, TBARS	AOPP, TAS, SH, SOD
−	Emsley et al. (2014)^60^	RCT	24996507	33/0	18–48	PUFA, ALA	24 or until relapse	Mean times to relapse	N/A	N/A	N/A	N/A
+	Roffman et al. (2013)^61^	RCT	23467813	140/0	18–68	FA, vit B12	4	**PANSS**, **SANS**, CDSS	N/A	N/A	RBC, folate levels, homocysteine, vit B12	N/A
+	Hill et al. (2011)^62^	RCT	21334854	32/0	46	FA	3	**SANS** modified, GAF, **PANSS**, CDSS	NAART, CVLT, FAS letter test, FAS animal test, **cognitive composite**	**QoL**	**Serum folate**, **RBC folate, vit B12**, homocysteine	N/A
+	Kuo et al. (2009)^63^	Case report	19892219	1/0	31	Vit B12	0.5	N/A	N/A	N/A	**Vit B12**	N/A
+	Berk et al. (2008)^64^	RCT	18436195	140/0	36.6 (10.9)	NAC	1	PANSS‐P, **PANSS‐T, PANSS‐N, PANSS‐G**, **CGI**, SOFAS, BARS, SAS, AIMS	N/A	GAF	N/A	N/A
+	Sivrioglu et al. (2007)^65^	Open‐label	17688987	17/0	18–55	PUFA, vit E, vit C	4	**BPRS**, **SANS**, **SAS**, **BARS**	N/A	N/A	Vit E, vit C	RBC‐MDA, **RBC‐SOD**, GPx

*Note*: Significant (+) or no (−) improvement in any of the parameters assessed. Measures in bold indicate a significant improvement in the intervention for that measure. Age is presented as mean (SD) or range.

*Nutraceutical supplements*: ALA, alpha‐lipoic acid; FA, folic acid; CoQ10, coenzyme Q10 (ubiquinone); NAC, N‐acetylcysteine; PUFA, polyunsaturated fatty acids; Vit, vitamin.

*Measures*: 25(OH)D, 25‐hydroxyvitamin D3; AIMS, Abnormal Involuntary Movement Scale; ALT, alanine aminotransferase activity; AOPP, Advanced Oxidation Protein Products; AQoL, Assessment of Quality of Life; ASSR, auditory steady‐state response; AST, aspartate aminotransferase activity; BACS, Brief Assessment of Cognition in Schizophrenia; BARS, Barnes Akathisia Rating Scale; BDNF, brain‐derived neurotrophic factor; BMI, body mass index; BPRS, Brief Psychiatric Rating Scale; CDS, Calgary Depression Scale; CDSS, Calgary Depression Scale for Schizophrenia; CGI, Clinical Global Impression; CGI‐S, CGI‐Severity Scale; CoQ10, coenzyme Q10; COWAT‐FAS, Fluency and Controlled Oral Word Association Test; CPT‐IP, Continuous Performance Test‐Identical Pairs version; CRP, C‐reactive protein; CVLT, California Verbal Learning Test; CYS_Pl_, plasmatic cysteine; DBP, diastolic blood pressure; FA, folic acid; FLI, fatty liver index; fMRI, functional magnetic resonance imaging; GAF, Global Assessment of Functioning; GGT, gamma‐glutamyl transferase; GP_X_, glutathione peroxidase; GSH, reduced glutathione; GSH_BC_, blood cell GSH; GSH_mPFC_, brain GSH; GPx_BC_, blood cell GSH peroxidase activity; HbA1c, haemoglobin A1c; HDL‐c, high‐density lipoprotein cholesterol; hs‐CRP, high‐sensitivity C‐reactive protein; IDO, indoleamine 2,3‐dioxygenase activity; IL‐1β, interleukin 1β; IL‐4, interleukin 4; LDL‐c, low‐density lipoprotein cholesterol; MANSA, Manchester Short Assessment of Quality of Life; MCCB, MATRICS (Measurement and Treatment Research to Improve Cognition in Schizophrenia) Consensus Cognitive Battery; MDA, malondialdehyde; MetS, metabolic syndrome; MMN, Mismatch Negativity; MMSE, Mini‐Mental State Examination; MoCA, Montreal Cognitive Assessment; MPO, myeloperoxidase; MRI, magnetic resonance imaging; MUFA, monounsaturated fatty acids; NAART, National Adult Reading Test; PANSS, Positive and Negative Syndrome Scale; PANSS‐G, PANSS general score; PANSS‐N, PANSS negative score; PANSS‐P, PANSS positive score; PANSS‐T, Positive and Negative Syndrome Scale total score; PSP, Personal and Social Performance; PUFA, polyunsaturated fatty acids; QoL, Quality of Life; RAVLT, Rey Auditory Verbal Learning Test; RBC, red blood cells; S100 PG, S100 calcium‐binding protein; SAFTEE, Systematic Assessment for Treatment Emergent Events; SANS, Scale for the Assessment of Negative Symptoms; SAPS, Scale for the Assessment of Positive Symptoms; SAS, Simpson Angus Scale; SBP, systolic blood pressure; SCoRS, Schizophrenia Cognitive Rating Scale; SFA, saturated fatty acids; SH, sulfhydryl groups; SOD, superoxide dismutase; SOFAS, Social and Occupational Functioning Assessment Scale; SWM, spatial working memory; TAC, total antioxidant capacity; TAS, total antioxidant status; TBARS, thiobarbituric acid‐reactive substances; T‐c, total cholesterol; TG, triglycerides; TMT, Trail Making Test; UKU‐SERS, Udvalg for Kliniske Undersøgelser Side Effect Rating Scale; Vit, vitamin; WC, waist circumference; WHR, waist‐to‐hip ratio; WMS, Wechsler Memory Scale; YMRS, Young Mania Rating Scale.

Abbreviations: C, controls; m, months; N/A, unassessed outcomes; P, patients; PMID, PubMed identifier; RCT, randomised controlled trial; SCZ, schizophrenia; SD, standard deviation; y, years.

**TABLE 3:** Nutraceutical supplementation and outcome measures evaluated in patients with MDD or related depressive symptoms

Results	Author, year	Study design	PMID	Study population		Nutraceutical intervention		Outcome measures		
				*n*	Age (y) in P/C	Component	Treatment time (m)	Clinical symptoms	Quality of life	Biological markers
				P/C						
+	Rahman et al. (2023)^66^	RCT	36462182	20 487/0	69.3	Vit D3	60	**PHQ‐9**	N/A	N/A
−	Kumar et al. (2022)^67^	RCT	35843459	59/0	37 (11)	Vit D3	3	HAM‐D, MADRS, BDI, CGI‐S, CGI‐I	N/A	25(OH)D
+	Amini et al. (2022)^68^	RCT	31900080	81/0	28 (1)	Vit D3	2	**EPDS**	N/A	**25(OH)D,** Ca, TNF‐α, IL‐6, oestradiol
+	Afsharfar et al. (2021)^69^	RCT	33745609	46/21	53.7 (7.7)	Mg	2	**BDI**	N/A	BDNF, **Mg**
−	Okereke et al. (2020)^70^	RCT	32749491	0/18 353	67.4 (7)	Vit D3 + EPA/DHA	63.6	PHQ‐8	N/A	25(OH)D
+	van der Burg et al. (2020)^71^	Post hoc analysis of RCT	31555976	96/0	18–70	SAMe, folinic acid, vit B12, PUFA, 5‐HTP, Zn, vit B6, vit C, Mg	2	N/A	N/A	**Folate, vit B12, Zn,** homocysteine, **BDNF, RBC‐PUFA**
+	Libuda et al. (2020)^72^	RCT	32108263	113/0	11–18.9	Vit D3	6	BDI‐II, **DISYPS‐II**	N/A	**25(OH)D**
+	Alghamdi et al. (2020)^73^	Open‐label	31836995	62/0	41.5 (1.8)	Vit D3	3	**BDI**	N/A	**VitD3, serotonin**
+	Kaviani et al. (2020)^74^	RCT	32217340	56/0	43 (1.2)	Vit D3	2	**BDI‐II**	N/A	**25(OH)D**, iPTH, oxytocin, platelet serotonin
−	Hansen et al. (2019)^75^	RCT	30944021	62/0	16–65	Vit D3	3	HAM‐D, MDI	WHO‐5	Weight, WC, blood pressure, 25(OH)D, CRP, phosphate, Ionised Ca, PTH
+	Dartois et al. (2019)^76^	Case series	31058543	10/0	14.4 (2.8)	FA	4–18.25	Subjective improvement in **depression, anxiety and irritability** symptoms	N/A	N/A
−	Bot et al. (2019)^77^	RCT	30835307	1025/0	46.5	PUFA, Se, FA, vit D3, Ca, behavioural therapy	12	12‐month cumulative onset of an episode of MDD, PHQ‐9, IDS30‐SR, GAD‐7, SQUASH, body weight perception	EQ‐5D‐5L	TEFQ‐R18, GA^2^LEN‐FFQ
−	Mousa et al. (2018)^78^	Mixed (cross‐sectional + RCT)	28803880	0/48	32 (8.5)	Vit D3	4	BDI	N/A	25(OH)D, anthropometric data
+	Bahrami et al. (2018)^79^	Nonrandomised pre‐post intervention	28759290	940/0	14.6 (1.5)	Vit D3	2.25	**BDI‐II**, Buss‐Perry aggression Questionnaire	N/A	**25(OH)D**
+	Tarleton et al. (2017)^80^	Open‐label	28654669	126/0	52.7	Mg	1.5	**PHQ‐9, GAD‐7**	N/A	N/A
+	Rajizadeh et al. (2017)^81^	RCT	28241991	60/0	20–60	Mg	2	**BDI‐II**	N/A	N/A
+	Vaziri et al. (2016)^82^	RCT	27544544	0/153	26.3 (4.6)	Vit D3	2.5–3	**EPDS**	N/A	**25(OH)D**
+	Wang et al. (2016)^83^	RCT	27022679	726/0	53.2	Vit D3	13	**BDI‐II**	N/A	Ca, P, **iPTH**, **25(OH)D**, albumin, prealbumin
−	Bedson et al. (2014)^84^	RCT	25052890	440/0	19–81	FA	3	BDI‐II, CGI, MADRS, UKU‐SERS	EQ‐5D‐5L, SF‐12	FA, vit B12, homocysteine
+	Wang et al. (2013)^85^	RCT	23885048	52/0	10.5 (1.5)	Vit C	0.33	**POMS‐B, DT**	N/A	**Vit C**, **25(OH)D, PTH,** CRP
−	Christensen et al. (2011)^86^	RCT	20805005	900/0	60–74	FA, vit B12	6	K‐10, PHQ‐9	N/A	Folate, homocysteine
+	Almeida et al. (2010)^87^	RCT	20976769	563/0	63.0 (11.4)	FA, vit B6, vit B12	12–126	Onset of DSM‐IV major depression, prevalence of major/minor depression	N/A	N/A

*Note*: Significant (**+**) or no (**−**) improvement in any of the parameters assessed. Age is presented as mean (SD) or range.

*Nutraceutical supplements*: 5‐HTP, 5‐hydroxytryptophan; Ca, calcium; DHA, docosahexaenoic acid; EPA, eicosapentaenoic acid; FA, folic acid; Mg, magnesium; NAC, N‐acetylcysteine; PUFA, polyunsaturated fatty acids; SAMe, S‐adenosylmethionine; Se, selenium; Vit, vitamin; Zn, zinc.

*Measures*: 25(OH)D, 25‐hydroxyvitamin D3; BDI, Beck Depression Inventory; BDI‐II, BDI‐Second Edition; BDNF, brain‐derived neurotrophic factor; BMI, body mass index; Ca, calcium; CGI, Clinical Global Impression; CGI‐I, CGI for Improvement; CGI‐S, CGI for severity of illness; CRP, C‐reactive protein; DISYPS‐II, Diagnostic System for Mental Disorders in Childhood and Adolescence, Self‐ and Parent Rating; DSM‐IV, Diagnostic and Statistical Manual of Mental Disorders, Fourth Edition; DT, distress thermometer; EPDS, Edinburgh Postnatal Depression Scale; EQ‐5D‐5L, health‐related quality of life developed by the EuroQol Group; FA, folic acid; GAD‐7, Generalised Anxiety Disorder 7 item scale; GA^2^LEN‐FFQ, Global Allergy and Asthma European Network food frequency questionnaire; GSI, Global Severity Index; HAM‐A, Hamilton Anxiety Rating Scale; HAM‐D, Hamilton Depression Rating Scale; IDS30‐SR, Inventory of Depressive Symptomatology; IL‐6, interleukin 6; iPTH, intact‐Parathyroid Hormone; K‐10, Kessler Psychological Distress Scale; MADRS, Montgomery‐Asberg Depression Rating Scale; MDD, major depressive disorder; MDI, Major Depression Inventory; Mg, magnesium; MSS, Mania Self Rating Scale; P, phosphorous; PHQ‐8/9, Patient Health Questionnaire; POMS‐B, Profile of Mood States—Brief; PSDI, Positive Symptom Distress Index; PST, Positive Symptom Total; PTH, parathyroid hormone; PUFA, polyunsaturated fatty acids; RBC, red blood cells; SCL‐90, Symptom Check List; SF‐12, 12‐Item Short Form Survey; SQUASH, Short Questionnaire to Assess Health Enhancing Physical Activity; TEFQ‐R18, Three Factor Eating Questionnaire‐ Revised; TNF‐α, tumour necrosis factor‐alpha; UKU‐SERS, Udvalg for Kliniske Undersøgelser Side Effect Rating Scale; YMRS, Young Mania Rating Scale; WC, waist circumference; WHO‐5, World Health Organisation‐Five Well‐Being Index; WHR, waist‐to‐hip ratio; Zn, zinc.

Abbreviations: C: controls; d: days; MDD: major depressive disorder; m: months; N/A: unassessed outcomes; P: patients; PMID: PubMed identifier; RCT: randomised controlled trial; SD: standard deviation; w: weeks; y: years.

**TABLE 4:** Nutraceutical supplementation and outcome measures evaluated in patients with ADHD, BD or bipolar spectrum disorders

Results	Author, year	Study design	PMID	Study population		Nutraceutical intervention		Outcome measures				
				*n*	Age (y) in P/C	Component	Treatment time (m)	Clinical symptoms	Cognitive function	Biological Behavioural outcomes	Biological markers	Oxidative stress
				P/C								
ADHD												
+	Yang et al. (2023)^88^	Cross‐sectional	37904452	80/80	7.39 (2.1)	Vit D3	0.5–1.5	**SNAP‐IV, PSQ**	N/A		Serum 25(OH)D	N/A
+	Samadi et al. (2022)^89^	RCT	36052304	75/0	8.6 (0.3)	Vit D3	3	N/A	N/A		**25(OH)D**, IL‐6, TNF‐α	N/A
+	Mohammadzadeh et al. (2022)^90^	RCT	35685610	75/0	8.6 (0.3)	Vit D3	3	N/A	N/A		**25(OH)D**,	PON‐1 activity, TAC, 8‐isoprostane
+	Bortolasci et al. (2021)^91^	Post hoc analysis of RCT	34438354	60/0	46.3 (10.8)	NAC	4	**MADRS**	N/A		**Metabolites**, glucagon, **amino acids**	N/A
+	Hemamy et al. (2021)^92^	RCT	33865361	66/0	9.1 (1.6)	Mg, vit D	2	**SDQ**	N/A		**25(OH)D, Mg**	N/A
+	Surman et al. (2019)^93^	RCT	30566416	41/0	39.5 (10.1)	FA	3	CGI, AISRS, GAF, HAM‐A, HAM‐D, SAS‐SR, CBS‐SR, BRIEF‐A, **ASR**	**CANTAB**		N/A	N/A
+	Mohammadpour et al. (2018)^94^	RCT	27924679	62/0	7.8 (1.6)	Vit D3	2	**ADHD‐RS,** CPRS, **WPREMB**	N/A		**25(OH)D**	N/A
+	Huss et al. (2010)^95^	Observational cohort	20868469	810/0	8.6	PUFA, Mg, Zn	3	**SDQ, SNAP‐IV**	N/A			N/A
**−**	Raz et al. (2009)^96^	RCT	19364294	63/0	10.5 (1.5)	PUFA, vit E, vit C	1.75	Conners scale short version (by parents and teachers)	N/A		Haematological parameters, PUFA	N/A
BD or bipolar spectrum disorders												
−	Badrfam et al. (2021)^97^	RCT	34662000	50/0	18–65	Vit B6	2	YMQ	MMSE	PSQI, appetite questionnaire	Homocysteine, inflammatory markers, fat profile, lab tests, anthropometric measures	N/A
+	Ashton et al. (2020)^98^	Post hoc analysis of RCT	31661974	133/0	> 18	NAC alone or a combination treatment including NAC	4	**MADRS**, **BDRS**, LIFE‐RIFT, **SOFAS**, **CGI‐I**	N/A	N/A	N/A	N/A
+	Jahangard et al. (2019)^99^	RCT	31346916	89/0	38.5 (10.8)	CoQ10	2	**MADRS**	N/A	N/A	**TNF‐α**, IL‐6, **IL‐10**	**TAC**, **TTG**, CAT, **NO**, MDA
+	Rapado‐Castro et al. (2017)^54^	Post hoc analysis of RCT	27894373	58/0	39.8 (12.3)	NAC	6	N/A	Attention, **WM**, executive function	N/A	N/A	N/A
−	Marsh et al. (2017)^100^	RCT	28777983	33/0	44.3	Vit D3	3	MADRS, HAM‐A, YMRS	N/A	N/A	N/A	N/A
+	Sikoglu et al. (2015)^101^	Open‐label	26091195	35/0	12.2 (3.3)	Vit D3	2	**YMRS**, CGI‐S, **CDRS**, CSSR‐S	N/A	N/A	MRI (ACC metabolites including glutamate, **GABA**, glucose, creatine and lactate, among others)	N/A

*Note*: Significant (**+**) or no (**−**) improvement in any of the parameters assessed. Measures in bold indicate a significant improvement in the intervention for that measure. Age is presented as mean (SD) or range.

*Nutraceutical supplements*: ALA, alpha‐lipoic acid; CoQ10, coenzyme Q10 (ubiquinone); EFA, essential fatty acids; FA, folic acid; Mg, magnesium; NAC, N‐acetylcysteine; PUFA, polyunsaturated fatty acids; Vit, vitamin; Zn, zinc.

*Measures*: 25(OH)D, 25‐hydroxyvitamin D3; ACC, anterior cingulate cortex; ADHD‐RS, Attention Deficit and Hyperactivity Disorder Rating Scale; AISRS, Adult ADHD Investigator Symptom Report Scale; ASR, Adult Self Report; BDRS, Bipolar Depression Rating Scale; BRIEF‐A, Behaviour Rating Inventory of Executive Function—Adult form; CANTAB, Cambridge Neuropsychological Test Automated Battery; CAT, catalase activity; CBS‐SR, Barkley Current Behaviour Scale—Self Report; CDRS, Children's Depression Rating Scale; CGI, Clinical Global Impression; CGI‐I, CGI—Improvement; CGI‐S, CGI—Severity; CPRS, Conner's Parent Rating Scale; CSSR‐S, Columbia‐Suicide Severity Rating Scale; GABA, gamma‐aminobutyric acid; GAF, Global Assessment of Functioning; HAM‐A, Hamilton Anxiety Rating Scale; HAM‐D, Hamilton Depression Rating Scale; HDL‐c, high‐density lipoprotein cholesterol; IL‐6, interleukin 6; IL‐10, interleukin‐10; LDL‐c, low‐density lipoprotein cholesterol; LIFE‐RIFT, Longitudinal Interval Follow‐Up Evaluation—Range of Impaired Functioning Tool; MADRS, Montgomery‐Asberg Depression Rating Scale; MDA, malondialdehyde; Mg, magnesium; MMSE, Mini‐Mental State Examination; MRI, magnetic resonance imaging; NO, nitric oxide; PON‐1, paraoxonase‐1; PSQ, Parent Symptom Questionnaire; PSQI, Pittsburgh Sleep Quality Index; SAS‐SR, Simpson Angus Scale—Self Report; SDQ, Strengths and Difficulties Questionnaire; SNAP‐IV, Swanson, Nolan and Pelham Rating Scale; SOFAS, Social and Occupational Functioning Assessment Scale; TAC, total antioxidant capacity; T‐c, total cholesterol; TG, triglycerides; TNF‐α, tumour necrosis factor‐alpha; TTG, total thiol groups; WM, working memory; WPREMB, Weekly Parent Ratings of Evening and Morning Behaviour; YMQ, Young Mania Questionnaire; YMRS, Young Mania Rating Scale.

Abbreviations: ADHD, attention‐deficit hyperactivity disorder; BD, bipolar disorder; C, controls; d, days; m, months; N/A, unassessed outcomes; P, patients; PMID, PubMed identifier; RCT, randomised controlled trial; SD, standard deviation; w, weeks; y, years.

### Vitamin D3 supplementation

After the review process, 33 articles were selected for further analysis (Supporting Information S2: table S2). A wide range of doses was used with different routes of administration (oral and intramuscular) and dosing regimens (daily, weekly, monthly or single dose). Treatment duration generally ranged from 4 to 60 weeks, although some studies reported substantially longer follow‐up periods of up to 2, 5 or 5.3 years.

In psychotic disorders, results were mixed and differed according to diagnosis and intervention. In patients with early psychosis, Gaughran et al.^102^ found no significant differences between the vitamin D3 and placebo groups in mental health or metabolic outcomes. In patients with chronic SCZ treated with clozapine, Krivoy et al.^57^ likewise found no significant effect of vitamin D3 on psychotic, depressive or metabolic parameters, although a trend towards improved cognition was reported. In contrast, in patients with SCZ, Ghaderi et al.^45^ reported that vitamin D3 combined with probiotics improved Positive and Negative Syndrome Scale (PANSS) scores and metabolic parameters.

For ASD, eight studies evaluated vitamin D3 supplementation, although three of them were case reports. Among controlled studies, Feng et al.^25^ reported significant clinical improvement with reductions in ASD rating scales and with more pronounced effects in younger children. Likewise, Saad et al.^27^ found that higher serum 25(OH)D concentrations were associated with greater improvement in ASD rating scales. However, Mazahery et al.^17^ found no significant improvement in core ASD symptoms with vitamin D3 alone, and Kerley et al.^26^ reported improvement in self‐care but not in the primary endpoint of stereotypic behaviour.

In ADHD, three studies evaluated vitamin D3 supplementation. Mohammadpour et al.^94^ assessed vitamin D3 as an adjunct to methylphenidate and found significant differences between the vitamin D3 and placebo groups in evening behaviour and total scores on the weekly parent ratings of evening and morning behaviour, although no differences were observed in other ADHD scale scores. By contrast, Samadi et al.^89^ and Mohammadzadeh et al.^90^ focused mainly on biochemical outcomes and reported significant increases in serum 25(OH)D levels after supplementation.

In studies focused on assessing improvements in depressive symptoms, findings were heterogeneous. Among the larger studies, Rahman et al.^66^ found no overall benefit of monthly vitamin D3 supplementation on depressive symptoms in older adults, although subgroup analyses suggested possible benefit in participants taking antidepressants at baseline and in those with lower predicted 25(OH)D concentrations. Okereke et al.^70^ and Vyas et al.^103^ found no significant benefit of vitamin D3 supplementation on the incidence of depression or on mood scores in individuals at risk of depression. Similarly, Kumar et al.^67^ and Hansen et al.^75^ reported no significant differences between vitamin D3 and placebo in depressive symptom scores in individuals presenting MDD and vitamin D deficiency or depressive symptoms, respectively. However, other studies reported improvements in depressive outcomes. Kaviani et al.^74^ found improved Beck Depression Inventory (BDI)‐II scores in subjects with mild to moderate depression, and Alghamdi et al.^73^ reported lower BDI scores after supplementation, particularly in women and in men with severe depression. Wang et al.^83^ found a significant association between supplementation and improvement in BDI‐II scores in dialysis patients with vascular depression, but not in MDD overall. In postpartum depression, Amini et al.^68^ reported significant reductions in depression scores in vitamin D3 supplementation groups compared with the placebo group, with a larger effect when vitamin D3 was combined with calcium. In pregnant women, Vaziri et al.^82^ found greater reductions in depression scores in the intervention group during late pregnancy and in the first weeks postpartum. In adolescents with depressive symptoms, Libuda et al.^72^ reported improvement in parent‐rated depressive symptoms but not in BDI‐II scores, whereas in adolescent girls, Bahrami et al.^79^ found a reduction in depression scores.

### NAC supplementation

We reviewed 19 studies that used NAC supplementation in patients with SCZ or SCZ‐related disorders (9 studies), ASD (6 studies), BD (2 studies), PTSD (1 study) and treatment‐resistant depression (1 study) (Supporting Information S2: table S3). Doses administered ranged from 0.45 to 3.6 g/d for at least 4 weeks. Two studies found a significant improvement in the Aberrant Behaviour Checklist (ABC) irritability subscore after 8 and 12 weeks of treatment compared with placebo,^33^, ^34^ but no changes in core symptoms of ASD were observed,^33^ and no statistical differences were found between the placebo and NAC groups on any other outcome measures.^21^, ^28^ Several reports have examined the use of NAC in early psychosis and SCZ, focusing on negative symptoms, diagnostic imaging tests and other clinical scaling data. Increased functional connectivity along the cingulum, particularly between the caudal anterior part and the isthmus of the cingulate cortex, has been observed using functional magnetic resonance imaging.^46^ However, another magnetic resonance imaging study found no changes in brain morphology.^49^ In addition, other studies have reported a significant improvement in neurocognitive functions, including attention, short‐term and working memory, executive function and processing speed,^50^, ^51^, ^54^ as well as a NAC‐associated increase in neural synchrony as assessed by the auditory steady‐state response in SCZ.^47^ Overall, most studies reported an improvement in global PANSS scores with the use of NAC,^49^, ^51^, ^64^ except for one study in which there was no significant difference between groups.^50^ Notably, two of these studies also reported improvements in negative symptoms. However, in treatment‐resistant SCZ, Neill et al.^41^ evaluated the efficacy of NAC and found no significant improvement in negative symptoms, overall cognition or quality of life over a 1‐year treatment period.

### ALCAR supplementation

Only one study met the inclusion criteria for ALCAR supplementation (Supporting Information S2: table S4). In this case report, a 4‐year‐old male with ASD and a *TMLHE* (trimethyllysine hydroxylase, epsilon) gene mutation was given L‐carnitine at a dosage of 200 mg/kg/day for 4.5 months. After supplementation, the child showed progress in achieving developmental milestones, and plasma carnitine levels returned to normal.^31^

### CoQ10 supplementation

Three studies reported the results of CoQ10 supplementation in SCZ, BD and ASD, using doses ranging from 0.03 to 0.3 g/d for at least 8 weeks (Supporting Information S2: table S5). No notable effects were observed in SCZ. However, some beneficial effects of CoQ10 were reported in BD, including improvements in inflammatory and oxidative stress biomarkers and reductions in depressive symptoms,^99^ as well as in ASD, where improved sleep patterns and relief of gastrointestinal problems were observed in children.^103^

### ALA supplementation

We identified six studies evaluating ALA supplementation in individuals with SCZ and associated disorders at doses ranging from 0.1 to 0.5 g/d for at least 45 days (Supporting Information S2: table S6). One study evaluated the efficacy of combining ALA with polyunsaturated fatty acids (PUFA) supplementation to prevent relapse after discontinuation of antipsychotics in individuals who had been successfully treated for 2–3 years after a first episode of SCZ, schizoaffective disorder or schizophreniform disorder. However, no differences in the mean time to relapse were observed between the intervention and placebo groups.^60^ Similarly, another study conducted by De Lima et al.^40^ in 2023 found no significant improvement in psychopathology, cognition, antipsychotic side effects or oxidative stress and inflammation after supplementation.^40^ Notably, two studies reported that ALA supplementation improved psychopathology in patients diagnosed with SCZ,^42^, ^55^ whereas the remaining two studies assessed oxidative stress and metabolic markers but did not assess psychopathological measures.^56^, ^59^ These studies found that ALA supplementation reduced lipid peroxidation and protein oxidative damage and improved nonenzymatic antioxidant capacity in healthy controls, but no significant changes were found in patients with SCZ.^59^

### Mg supplementation

Supporting Information S2: table S7 includes five reports on the use of Mg treatment for ADHD, MDD and ASD, at doses ranging from 0.06 to 0.5 g/d for at least 6 weeks. All the studies reported some significant improvements. Three independent studies found a significant reduction in depressive symptoms in patients with MDD, as shown by the results of different assessments using different questionnaires.^69^, ^80^, ^81^ In addition, a study combining Mg and vitamin D showed a significant reduction in emotional, conduct and peer problems and a significant increase in other behavioural scores in individuals with ADHD.^92^ Furthermore, two children with ASD who were treated with Mg and vitamin B6 showed a significant improvement in hyperactive behaviour.^16^

### Vitamin B6 supplementation

Five studies reported results of vitamin B6 supplementation for various psychiatric disorders, including ASD, BD, SCZ and tic disorders. The trials administered doses ranging from 0.003 to 2.4 g/d for a minimum of 8 weeks, as shown in Supporting Information S2: table S8, although three of them used vitamin B6 in combination with other nutraceutical supplements. The general findings highlight the lack of any significant benefit from vitamin B6 supplementation, independent of the use of other nutraceuticals. In fact, one study of patients with BD reported an improvement in cognitive status in the placebo group.^97^ Similarly, patients with tic disorders have shown a greater reduction in tic severity in the psychoeducational intervention group,^104^ compared with those who received vitamin B6 supplementation. In patients with ASD, vitamin B6 and vitamin B12 supplementations were found to be effective in reducing urinary homocysteine levels. This effect was enhanced when the combination was supplemented with FA.^105^

### Vitamin B7 supplementation

Although one report met the criteria for inclusion in this review because it included vitamin B7 supplementation (Supporting Information S2: table S9), vitamin B7 was not the target of the study as it was included in both the intervention and placebo groups.^106^

### FA supplementation

Eleven studies (Supporting Information S2: table S10) evaluated the effects of FA, either alone (in eight studies), combined with vitamin B12 (in two studies), or with behavioural analysis or structured education (in one study). The doses of FA used varied from 400 μg to 15 mg, and the duration of treatment ranged from 12 weeks to 24 months. The diagnoses studied were ADHD,^93^ ASD,^22^ MDD,^76^, ^84^ eating disorders,^107^ SCZ,^53^, ^61^, ^62^ and depressive symptoms.^86^ FA supplementation has been reported to improve adaptive functioning in ADHD patients,^93^ and cognitive and depressive status in people with eating disorders.^107^ Sociability, cognitive verbal/preverbal, receptive language, affective expression and communication improved in individuals with ASD who received FA and structured teaching compared to those in the placebo group receiving only structured teaching.^29^ A study of patients with SCZ reported that FA improved symptomatology as assessed by the PANSS, specifically, the total score (PANSS‐T), the negative score (PANSS‐N) and the general score (PANSS‐G). Improvement in PANSS‐T and PANSS‐G scores was influenced by the genetic background in folate‐related genes, whereas improvement in PANSS‐N scores was observed regardless of genetic background.^53^ In contrast, previous reports have suggested that improvement in PANSS‐N scores depended on an individual's genetic background in folate‐related genes and their serum folate levels.^53^, ^61^, ^62^ Another study found similar results in people who received a combination of FA and vitamin B12. The treatment led to a significant improvement in negative symptoms compared with placebo when the genetic variant rs202676 of the folate hydrolase 1 (*FOLH1*) gene was taken into account.^61^ Conversely, supplementation with 5 mg FA for 12 weeks showed no evidence of clinical efficacy in improving antidepressant treatment in patients with MDD.^84^

### Vitamin B12 supplementation

The efficacy of vitamin B12 has been evaluated in combination with FA, vitamin B6 or both (Supporting Information S2: table S11). Notably, in a case report study by Kuo et al.,^63^ a 31‐year‐old man with below‐average cobalamin levels and symptoms suggestive of SCZ experienced symptom relief and recovery of vitamin B12 levels following administration of 1 mg of vitamin B12 for 2 weeks. The management of negative symptoms in patients with SCZ is improved by the administration of vitamin B12 and FA, as noted in the section of FA supplementation.^53^, ^61^ Furthermore, the section on vitamin B6 supplementation highlights the efficacy of the combination of vitamin B12 and vitamin B6 in reducing urinary homocysteine levels in patients with ASD.^105^

### Vitamin E supplementation

None of the trials identified was designed to assess the benefits of vitamin E supplementation. Vitamin E was used primarily as an antioxidant to assess the effects of PUFA in 16 trials conducted in a wide range of psychiatric conditions (Supporting Information S2: table S12). The doses administered ranged from 0.002 to 0.8 g/d for at least 6 weeks. Only one trial evaluated the effects of combined vitamin A and vitamin E supplementation compared with PUFA supplementation in people with SCZ, schizoaffective disorder or schizophreniform disorder. This study, which was conducted using a placebo‐controlled 2 × 2 factorial design (placebo PUFA and placebo vitamins; placebo PUFA and active vitamin A + vitamin E; active PUFA and placebo vitamins; and active PUFA and active vitamin A + vitamin E), showed that vitamins or eicosapentaenoic acid (EPA) had an adverse effect on the course of psychotic symptoms (*d* = 0.40, *p* = 0.003; *d* = 0.37, *p* = 0.005, respectively), especially persecutory delusions (*d* = 0.48, *p* < 0.001; *d* = 0.47, *p* < 0.001, respectively). However, the combination of vitamin A, vitamin E and EPA neutralised the negative effect of each component on psychosis (interaction *d* = 0.31; *p* = 0.02).^108^

### Vitamin A supplementation

Only three studies evaluating vitamin A supplementation met the inclusion criteria (Supporting Information S2: table S13), and all of them focused on people with ASD. All of them used a single dose of 200 000 IU of vitamin A, except Lai et al., who used a single dose of 200 000 IU in one intervention group and a weekly formulation of 50 000 IU/week for 11 weeks, plus a 3000 IU/day for 13 weeks in the other intervention group. Treatment lasted 24 weeks in all three studies. Analysis of laboratory tests showed that, at baseline, children with severe ASD had significantly higher levels of 5‐hydroxytryptamine and significantly lower levels of retinol than children with mild to moderate ASD.^19^ After supplementation, retinol levels increased significantly, along with changes in other gene expression parameters (retinoic acid receptors, retinoic acid‐related orphan receptor alpha and CD38).^11^, ^19^, ^23^ Regarding ASD symptoms, one study reported a significant improvement in Childhood Autism Rating Scale (CARS) scores following a single treatment dose,^19^ whereas another found no significant differences in ABC, CARS and Social Responsiveness Scale (SRS).^23^ A third study reported positive results in SRS scores following weekly treatment.^11^

### Vitamin C supplementation

Three trials were included for vitamin C (Supporting Information S2: table S14), but only one of these evaluated vitamin C as a nutraceutical supplement. In this study, vitamin C was associated with a reduction in mood disturbance and psychological distress (71% and 51%, respectively), with a significant association between improved mood and increased plasma vitamin C concentrations.^85^

### Vitamin B3 supplementation

Although one study met the criteria for inclusion in this review (Supporting Information S2: table S15), vitamin B3 was not the study's target. Instead, it was used as an active placebo because it could induce an acute physiological response (flushing), thought to aid in blinding.^109^

### Multiple nutraceutical supplementation

The results of studies evaluating three or more nutraceutical components are summarised in Supporting Information S2: table S16. These reports combined a wide variety of components, including vitamins, PUFAs, probiotics, enzymes and salts, among others, in different doses and treatment durations and focused on different psychiatric disorders such as ADHD,^95^, ^96^ ASD,^20^, ^24^, ^35^, ^36^, ^38^, ^105^ BD,^110^, ^111^ MDD,^71^, ^87^, ^106^, ^112^ PTSD,^113^ SCZ,^65^ first‐episode psychosis^48^ and in individuals with depressive symptoms without a diagnosis in the past 6 months.^77^ Of these, Russell et al.,^111^ Dean et al.^110^ and Adams et al.^20^, ^35^ are the ones that proposed the use of more components in their assessments. In 2011, Adams et al. conducted a study of at least 30 supplements and found that these products could be beneficial for ASD patients by improving symptoms of hyperactivity, temper tantrums and language, among others; however, the difference from the placebo group was not significant for the other stated goals of the intervention.^35^ In the 2018 study, Adams et al. used a different nutraceutical cocktail of 32 supplements, this time achieving improvements in nonverbal intelligence quotient and a substantial 18‐month developmental gain in communication, daily living skills and social skills.^20^ On the other hand, Dean et al.^110^ and Russell et al.^111^ proposed studies in patients with BD using a cocktail of 17 supplements, although the specific components differed between studies. For Dean et al., only the study design and rationale were published, and the intervention was intended for patients with BD during the depressive phase.^110^ In contrast, Russell et al. reported no significant differences in depressive or manic symptoms over time between BD and BD + PTSD.^111^

Almeida et al. found the combination of FA, vitamin B6 and vitamin B12 was associated with a lower risk of MDD compared with placebo in survivors of stroke.^87^ However, in patients with psychosis, the same combination, but with almost double the doses of vitamin B6 and vitamin B12, had no significant effect on PANSS score or neurocognitive composite.^48^ Remarkably, in patients with ADHD, supplementation with PUFAs, magnesium and zinc showed a significant reduction in symptoms of attention deficit and hyperactivity/impulsivity in a large sample with a low incidence of adverse effects (1.7%).^95^

### Risk of bias analysis

During the ROB analysis, 25 articles were excluded because they were not compatible with the Cochrane RoB 2 tool. These articles were excluded for several reasons, including the lack of a control group, case report design or the absence of outcome data. Overall, most of the included trials showed methodological concerns in one or more domains, most commonly bias arising from the randomisation process, deviations from the intended interventions and missing outcome data. Ultimately, among the 97 included studies, the majority were rated as having some concerns or high ROB. Of the 45 studies rated as having some concerns, 35 were RCTs, whereas 44 studies (22 RCTs) were rated as having high ROB, indicating generally low methodological quality. Only eight trials were rated as having low ROB, all of which were RCTs. Detailed ROB assessments for each nutraceutical supplement are shown in figure S2.

### Summary of results by diagnosis

**FIGURE 2:**
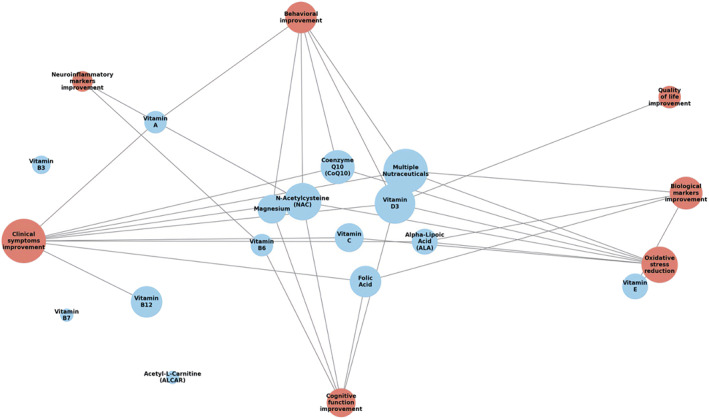
Relationship between mitochondrial‐targeting nutraceuticals and key associated outcomes. Blue nodes represent individual nutraceuticals (e.g., vitamin D3, N‐acetylcysteine and coenzyme Q10), whereas red nodes indicate outcome domains (e.g., clinical symptoms, cognitive function, oxidative stress and quality of life). Edges illustrate associations reported in the included studies, with node size reflecting the relative frequency of evidence supporting each nutraceutical or outcome.

Dietary supplements were extensively evaluated for ASD (28 studies, including nine RCTs; table 1), SCZ spectrum disorders (27 studies, including 18 RCTs; table 2), MDD or related depressive symptoms (22 studies, including 18 RCTs; table 3), as well as ADHD and BD spectrum disorders (9 and 6 studies, respectively, including 7 and 5 RCTs; table 4). Not all nutraceuticals were investigated under comparable methodological conditions or to the same extent across studies. Substantial heterogeneity was observed across nutraceutical type, dosage, intervention duration, study populations and clinical outcome measures. As a result, the available data could not be meaningfully extracted or pooled for quantitative synthesis. Therefore, the results are presented using a narrative approach, aimed at summarising patterns of evidence and highlighting conditions and compound‐specific trends, rather than deriving aggregated effect estimates. The main characteristics of each study, including study design, population, nutraceutical intervention, outcome measures and reported clinical improvements, are summarised in tables 1, 2, 3, 4. When analyses were restricted to RCTs and focused exclusively on clinical outcomes, improvement in at least one clinical domain was reported in five of nine studies conducted in ASD (two involving multiple nutraceuticals, two with NAC and one with Vit D3). In SCZ spectrum disorders, 9 of 18 studies reported clinical improvement (4 with NAC, 2 with FA, 1 with FA plus Vit B12, 1 with ALA and 1 with Vit D3). Similarly, 9 of 18 studies in MDD reported clinical improvement (6 with Vit D3, 2 with Mg and 1 with Vit C). In ADHD, clinical improvement was observed in four of seven studies (one with NAC, one with FA, one with Vit D3 and one with Mg plus Vit D), whereas in bipolar spectrum disorders, two of six studies reported positive effects (one with NAC and one with CoQ10). The relationship between different nutraceutical categories and key associated outcomes is shown in figure 2, highlighting the links between specific compounds and their potential mental health benefits.

## DISCUSSION

### Main findings

The use of nutritional interventions in the adjuvant treatment of psychiatric disorders, in the form of dietary or nutritional supplements, has developed rapidly in recent years,^114^ and there is increasing evidence describing a bioenergetic cause for psychiatric disorders. Commonly used psychotropic drugs affect the activity of mitochondrial complexes, and novel bioenergetic targeting therapies may improve the suboptimal response to currently available psychotropic treatments.^115^ We aimed to summarise the clinically relevant evidence for the efficacy of nutraceutical supplements that affect mitochondrial function in psychiatric disorders. Most studies using either a single nutraceutical or a combination of components reported clinical or metabolic improvements in ASD, SCZ, MDD, BD and ADHD. Meanwhile, there were too few studies to draw conclusions about the effects on psychiatric conditions such as chronic tic disorder, Tourette's syndrome, eating disorders, borderline personality disorder, posttraumatic stress disorder, postpartum depression and intellectual disability.

For ASD, 22 of the 28 studies reported a clinical benefit of nutraceutical supplementation. However, a variety of nutraceuticals were evaluated, and treatment duration was highly heterogeneous, ranging from 1 to 24 months. In particular, two nutraceuticals have been more frequently investigated as single components: vitamin D3 and NAC, each with six studies. For vitamin D3, two studies evaluated a large number of participants (*n* = 215 and *n* = 122) and reported a significant improvement in CARS scores.^25^, ^27^ For NAC, studies with 40 patients^33^ and 33 patients^34^ reported improvement in ABC scores, but a subsequent study in a larger sample (*n* = 102) found no improvement.^21^

In SCZ, 19 of 27 studies evaluating nutraceutical supplementation reported some clinical benefits. Several nutraceuticals were evaluated, with NAC and ALA being the most frequently studied, with nine and six studies, respectively. NAC improved SCZ symptoms in three studies with treatment durations of 4, 12 and 52 weeks,^49^, ^51^, ^64^ but NAC did not significantly improve SCZ symptoms in treatment‐resistant patients taking clozapine.^41^ Evidence for ALA supplementation is more limited: 2 studies including 10 and 20 patients reported clinical improvement,^42^, ^55^ but a more recent study including 35 patients found no clinical benefit.^40^

In MDD and related depressive symptoms, vitamin D3 has been studied extensively in adolescents and adult patients, often in large patient and control populations and mainly as a single agent. Vitamin D3 supplementation has been reported to improve depressive symptoms in the elderly,^66^ adult^73^, ^74^, ^83^ and adolescent^72^, ^79^ populations. Additionally, two studies reported reductions in depression scores in perinatal depression^82^ and postpartum depression.^68^ However, some studies found no evidence that vitamin D3 supplementation improved depressive symptoms,^67^, ^75^, ^78^ although it is worth noting that some of these studies focused on specific phenotypes, such as overweight/obesity and vitamin D deficiency, in addition to depressive disorders.

Nine studies evaluated the benefits of nutraceutical supplementation in ADHD, with all but one reporting some metabolic or clinical benefit. Vitamin D3 was evaluated as a single nutraceutical component in four studies. Of these, four evaluated metabolic parameters and three observed increases in 25(OH)D levels,^88^, ^89^, ^90^ whereas two assessed clinical outcomes, reporting improvements in ADHD‐Rating Scale, Weekly Parent Ratings of Evening and Morning Behaviour^18^ and in Attention‐Deficit Hyperactivity Disorder of Swanson, Nolan and Pelham, version IV and Parental Symptom Questionnaire scores.^88^

For BD, four studies reported some benefit from nutraceutical supplementation, whereas two studies reported no benefit. Two studies evaluated the benefit of vitamin D3 supplementation as a single component, one of which focused on adult patients with BD and found no evidence of clinical improvement as measured by the Montgomery–Asberg Depression Rating Scale or the Hamilton Anxiety Rating Scale,^100^ whereas the other study focused on adolescent patients with BD and found clinical improvement with significant reductions in Young Mania Rating Scale and Calgary Depression Rating Scale scores and increases in anterior cingulate cortex GABA.^101^

Vitamin D3 is the most extensively studied nutraceutical across psychiatric conditions, yet the evidence remains inconsistent. In psychotic disorders, available trials are limited and have not shown consistent clinical benefits, whereas large‐scale studies in the prevention of depression have failed to demonstrate an overall effect. Although some smaller trials report improvements in depressive symptoms, particularly in subgroups such as individuals with moderate to severe depression, perinatal populations or those with low baseline vitamin D levels, findings in ASD and ADHD are variable and constrained by small sample sizes and heterogeneous study designs. Across diagnoses, 16 RCTs have evaluated vitamin D3, of which 9 reported improvements in at least one clinical outcome; however, these effects were inconsistent and often confined to specific symptom domains or particular subgroups. Taken together, current evidence does not support a generalised therapeutic effect of vitamin D3 on psychiatric outcomes, although context‐dependent benefits in selected populations cannot be ruled out.

The role of mitochondrial dysfunction in psychiatric disorders has received increasing attention in recent years. Mitochondria, the powerhouses of cells, play a critical role in energy production, regulation of oxidative stress and neurotransmitter metabolism, all of which have been implicated in the pathophysiology of psychiatric disorders. This systematic review identified several studies that have investigated the potential benefits of nutraceuticals targeting mitochondrial function in psychiatric disorders. Some of these studies used randomised controlled designs and objective measures of the mitochondrial function, such as mitochondrial enzyme activity and markers of oxidative stress, whereas others did not. In addition, some studies have used neuroimaging techniques, such as magnetic resonance imaging, to assess changes in brain bioenergetics after supplementation with nutraceuticals, specifically in NAC,^46^, ^49^, ^50^, ^58^ FA^53^ and vitamin D3.^101^

### Limitations

There are several limitations of this study, including small sample sizes, short duration and heterogeneity of treatment options and protocols. The included studies generally reported the active compound and dosage of the supplements administered; however, detailed information about manufacturers, formulation standardisation or independent verification of supplement composition was not provided. Consequently, potential variability in supplement formulation across studies cannot be completely ruled out. In addition, the complexity of psychiatric disorders, which includes different mood and psychotic states and varying symptom severity, poses challenges in assessing the treatment response and generalisability of findings. Innovative approaches, such as utilising platelets as biomarkers to predict drug responses in SCZ, may offer pathways for more personalised and effective strategies. These approaches could also be applied to explore the efficacy of nutraceutical interventions.^116^ Abnormalities in mitochondrial function have been implicated in the aetiology and progression of psychiatric disorders. Studies evaluating the efficacy of nutraceutical supplementation in psychiatric disorders have reported mixed results. Although some studies have demonstrated improvements in symptoms and cognitive function, others have failed to replicate these findings. Methodological limitations, including small sample sizes, short follow‐up periods and variability in outcome measures, may contribute to the observed inconsistency across studies. Additionally, we based this systematic review in the context of modern psychiatric diagnostic criteria and contemporary nutraceutical formulations; however, restricting the inclusion period to 1 January 2007 through 30 April 2024, limiting the search to English‐language publications and searching only three databases may have resulted in the omission of relevant studies.

### Implications

Over the past decade, significant progress has been made in elucidating the intricate structure, function and physiological implications of mitochondria, particularly in relation to metabolic syndromes such as diabetes, obesity, stroke, hypertension and heart disease. In addition, there has been remarkable progress in the field of therapeutic interventions, encompassing diverse modalities such as lifestyle modifications (including a healthy diet and regular exercise), pharmacological innovations and mitochondria‐targeted methodologies. These multifaceted strategies have primarily aimed to ameliorate mitochondrial dysfunction and mitigate oxidative stress while preserving mitochondrial integrity in the context of metabolic syndrome.^117^ The challenge will be to translate the progress made in metabolic disorders to mental disorders.

### Conclusions

Nutraceutical supplementation targeting mitochondrial function is a promising approach for the treatment of psychiatric disorders. Although preliminary evidence suggests potential benefits in conditions such as ASD, SCZ, MDD, ADHD and BD, further research is needed to clarify the efficacy and mechanisms of action. Addressing methodological limitations and standardising treatment protocols are essential steps in advancing our understanding of the role of nutraceuticals in improving mitochondrial function and mental health outcomes. Furthermore, socioeconomic factors, such as education or income, as well as intelligence, can influence access to and adherence to nutraceutical interventions. These considerations underline the need for inclusive strategies that account for demographic and socioeconomic disparities in psychiatric care.^118^

## FUNDING

This work was supported by the Instituto de Salud Carlos III, Grant Numbers PI21/01812 and PI24/01023; the Catalan Agency of research and Universities (AGAUR) 2021 SGR‐01065; and co‐funded by the European Union. Juan Tortajada and Paula Alcaide‐Barriga were the recipients of an industrial doctorate (2020 DI 00085 and 2024 DI 00109, respectively), and Bengisu Kevser Bulduk and Bernat Ballvé‐Gelonch were recipients of a grant for the recruitment of new research staff (2020 FI_B 00650 and 2024 FI_100149, respectively) from the Generalitat de Catalunya. Alba Valiente‐Pallejà received a Talent‐Health fellowship from the Diputació de Tarragona. The funders had no role in the study design, data collection and interpretation, writing of the report or the decision to submit the manuscript for publication.

## CONFLICT OF INTEREST STATEMENT

The authors declare no conflicts of interest.

## Supporting information

Supporting Information S1

Supporting Information S2

Figure S2

Table S1
