# Supplementary material for: Clinical outcomes of mitochondrial‐enhancing nutraceutical supplementation in psychiatric disorders: A systematic review
Source: Gen Psychiatr. 2026 Jun 1;39(3):e70023. doi: 10.1002/gps3.70023 (PMC13239229; doi:10.1002/gps3.70023)
Supplement: Supplementary file 1 — Supporting Information S1 [file GPS3-39-e70023-s004.docx]

**SUPPLEMENTAL APPENDIX**

1. **Composition, sources, functions and other relevant characteristics on mitochondrial cofactors, vitamins and minerals**

**Vitamin D3**

Known as cholecalciferol, and vitamin D2, also known as ergocalciferol, are the two main forms of vitamin D found in foods and dietary supplements. Vitamin D, a fat-soluble vitamin, occurs naturally in some foods and is also produced endogenously when ultraviolet rays from sunlight strike the skin, initiating vitamin D synthesis. Vitamin D obtained from sun exposure, foods, and supplements is biologically inert and requires two hydroxylations in the body for activation. The first hydroxylation, which occurs in the liver, converts vitamin D to 25-hydroxyvitamin D [25(OH)D], also known as “calcidiol.” Subsequent hydroxylation, primarily in the kidney, produces the physiologically active 1,25-dihydroxyvitamin D [1,25(OH)2D], also known as “calcitriol”. ^1^ Vitamin D promotes calcium absorption in the intestine and maintains adequate serum calcium and phosphate concentrations. It also plays multiple roles in the body, including reducing inflammation and modulating processes such as cell growth, neuromuscular function, immune function, and glucose metabolism. ^1,2^ Numerous genes encoding proteins that regulate cell proliferation, differentiation, and apoptosis are modulated, in part, by vitamin D. Many tissues possess vitamin D receptors, some of which convert 25(OH)D to 1,25(OH)2D. The current primary indicator of vitamin D status is the serum concentration of 25(OH)D. Research has previously described the association between low vitamin D levels and mental illness. A recent study reported that the average vitamin D level among patients at an academic psychiatric hospital was 23.59 ng/mL, which was below normal levels (>30 ng/mL). In addition, the authors found a significant association between vitamin D levels <20 ng/mL and symptoms of psychosis.^3^

**N-Acetyl Cysteine (NAC)**

NAC, the N-acetylated derivative of the natural amino acid L-cysteine, does not occur naturally but shares its origin with cysteine found in garlic, eggs, yogurt and chicken/turkey meat.^4,5^ According to EFSA, the reference intake of cysteine for adults is about 17 mg/kg per day, and the recommended daily intake of supplemental NAC varies between 1-2 capsules per day, containing 500-600 mg per capsule, depending on the product type. NAC serves to stimulate glutathione biosynthesis, promotes detoxification, and acts directly as a free radical scavenger. Recognized for its role as an antioxidant and glutathione inducer, it is indicated for mucolytic therapy and the treatment of acetaminophen overdose. In recent years, NAC has expanded its scope to be studied as an antioxidant, anti-infective, and antiviral agent, with a wide range of off-label indications. Randomized clinical trials have evaluated its efficacy in psychiatric disorders, including somatic symptom disorder, bipolar-related disorders, depressive disorders, anxiety disorders, obsessive-compulsive disorders, and substance use disorders.^6^

**ALCAR**

Carnitine, a collective term that includes L-carnitine, acetyl-L-carnitine (ALCAR), and propionyl-L-carnitine, serves as a carrier for long-chain fatty acyl-CoA into the mitochondria, facilitating beta-oxidation. Although carnitine can be obtained from the diet and is synthesized in the kidney, liver, and brain, it is considered a “conditionally essential” nutrient for humans, especially when intracellular levels are low. Human plasma and tissues, including the brain, contain both free L-carnitine and acylated derivatives with different carbon chain lengths.^7,8^ Notably, acetyl L-carnitine exhibits increased permeability across the blood-brain barrier compared to free carnitine. Its roles in energy metabolism are diverse, including facilitating the entry of acetyl-CoA into mitochondria during fatty acid oxidation, enhancing acetylcholine production, stimulating protein and membrane phospholipid synthesis, and preventing excessive neuronal cell death.^7^ In recent years, the therapeutic potential of carnitine for neuroprotection has attracted considerable interest. There is compelling evidence from preclinical studies that both L-carnitine and ALCAR can improve energy status, reduce oxidative stress, and prevent subsequent cell death in models of adult, neonatal, and paediatric brain injury. Recent reviews highlight acetyl-L-carnitine as a potentially effective and well-tolerated option for individuals suffering from depression.^7,8^

**Coenzyme Q10 (CoQ10)**

CoQ10, also known to as ubiquinone due to its quinone structure, is a lipid-soluble benzoquinone that can be synthesized by human cells in most tissues from the amino acid tyrosine; however, it is also found in meat, poultry, and fish.^9,10^ CoQ10 plays a critical role in the OXPHOS process and is essential to produce adenosine triphosphate (ATP), which is responsible for storing and transporting chemical energy within the cell.^11^ During periods of oxidative stress, CoQ10 increases DNA resistance to damage, serving as a potent antioxidant. In its reduced form (ubiquinol), CoQ10 scavenges excess reactive oxygen species, preventing critical damage and protecting membrane phospholipids, low-density lipoproteins, and mitochondrial membrane proteins from free radical-induced oxidative damage.^10,11^ In neurological and neuropsychiatric disorders, CoQ10 is used to support mitochondrial function, thereby improving overall brain function. The effects of CoQ10 supplementation have been studied in several conditions, including BD and SCZ.^10,11^

**Alpha-Lipoic acid (ALA)**

Also known as 1,2-dithiolane-3-pentanoic acid or thioctic acid, is an organosulfur lipid and water-soluble compound synthesized in mitochondria by the lipoic acid synthase system. Among plant sources, spinach stands out as the most beneficial, while kidney is the richest meat source of ALA. ALA is known to enhance and restore intrinsic antioxidant systems and actively participates in the removal of heavy metals from the bloodstream, which are known culprits of oxidative stress.^12^ In addition to its antioxidant properties, ALA, together with its reduced form, dihydrolipoic acid (DHLA), serves as a critical modulator of signal transduction in several central and peripheral 5´-AMP-activated protein kinase pathways.^13^ Some studies have demonstrated the beneficial effects of ALA, especially in patients with SCZ, demonstrating its ability to mitigate the side effects associated with antipsychotic drugs.^13–15^

**Magnesium (Mg)**

Mg is a vital cation that is essential for many functions within the central nervous system. It is the fourth most abundant mineral ion and is obtained primarily from foods such as green leafy vegetables, whole grains, nuts, and fish.^16^ As a cofactor in over 300 enzymatic reactions, Mg plays a critical role in maintaining the structural function of proteins, nucleic acids, and mitochondria. In addition, Mg has an anti-apoptotic function in the mitochondrial permeability transition and counteracts calcium overload-induced apoptosis.^17^ Inadequate Mg intake can lead to hypomagnesemia, which affects 2-15% of the general population and may pose a risk to metabolic and cardiovascular health. While the evidence regarding psychiatric conditions remains inconclusive, available data suggest that Mg supplementation may have beneficial effects.^16^

**Vitamin B6**

Vitamin B6 consists of six active compounds, including pyridoxine, pyridoxal, and pyridoxamine, each of which contains an amino group. Their respective 5’-phosphate esters, pyridoxal 5’-phosphate (PLP) and pyridoxamine 5’-phosphate (PMP), serve as the active coenzyme forms of vitamin B6. Found in abundance in a variety of foods, the richest sources of vitamin B6 include fish, beef liver, other organ meats, potatoes, other starchy vegetables, and fruits (excluding citrus fruits).^18,19^ Glycosylated forms of naturally occurring pyridoxine in fruits, vegetables, and grains have reduced bioavailability.^20^ Vitamin B6, in its coenzyme forms, plays a critical role in more than 100 enzymatic reactions, primarily associated with protein metabolism.^18,21^ It contributes to gluconeogenesis and glycogenolysis, immune function, haemoglobin formation, amino acid metabolism (by both PLP and PMP), and metabolism of one-carbon units, carbohydrates, and lipids (especially by PLP). In addition, vitamin B6 is essential for cognitive development through neurotransmitter synthesis and the regulation of homocysteine levels. Pyridoxine has a significant and selective modulating effect on central serotonin and GABA production, with studies exploring its potential to improve the treatment of anxiety or depression.^22^ Plasma PLP is the standard measure of vitamin B6 status, with concentrations above 30 nmol/L indicating adequate status in adults.^20^ However, the Food and Nutrition Board (FNB) of the Institute of Medicine of the National Academies uses a plasma PLP level of 20 nmol/L as the primary indicator of adequacy for calculating the Recommended Dietary Allowances (RDAs) for adults.^18,19^

**Vitamin B7**

Commonly known as biotin, acts as a cofactor for five carboxylases: propionyl-CoA carboxylase, pyruvate carboxylase, methylcrotonyl-CoA carboxylase [MCC], acetyl-CoA carboxylase 1, and acetyl-CoA carboxylase 2. These carboxylases are essential for the metabolism of fatty acids, glucose, and amino acids^18,23–25^ Vitamin B7 also plays a key role in histone modifications, gene regulation (by modifying the activity of transcription factors), and cell signalling. Foods rich in vitamin B7 include organ meats, eggs, fish, meat, seeds, nuts, and certain vegetables such as sweet potatoes.^26^ The amount of vitamin B7 in foods can vary depending on plant variety, season, and processing techniques. Most of the vitamin is bound to protein, while some dietary vitamin B7 is in a free form and is absorbed in the small intestine for storage in the liver.^18,27,28^ Abnormal or decreased urinary excretion of vitamin B7 may indicate a deficiency. The most reliable markers of individual vitamin B7 status, including both deficiency and sufficiency, are biotinylated MCC and white blood cell propionyl-CoA carboxylase.^26,29,30^ In a paediatric evaluation of 19 children, adolescents, and young adults with propionic acidaemia, ASD was reported to be a common disorder, highlighting the results of propionyl-CoA carboxylase activity.^31^

**Folic acid**

Vitamin B9, also known as folate, occurs naturally in some foods, is added to others, and is available as a dietary supplement. Dietary folates exist in the form of tetrahydrofolate (THF) and typically contain additional glutamate residues, forming polyglutamates. Folic acid (FA), the fully oxidized monoglutamate form of vitamin B9, is used in fortified foods and most supplements.^32^ Folate acts as a coenzyme or substrate in single-carbon transfers in the synthesis of nucleic acids (DNA and RNA) and in the metabolism of amino acids. The major form of folate in plasma is 5-methyl-THF. The estimated total body content of folate is 15-30 mg, with approximately half stored in the liver and the remainder in the blood and body tissues. Serum folate concentrations are commonly used to assess folate status, while erythrocyte folate concentrations provide a longer-term measure of folate intake.^32–35^ Plasma homocysteine concentration serves as a functional indicator of folate status because elevated levels can occur when the body is unable to convert homocysteine to methionine due to a 5-MTHF deficiency. However, homocysteine levels are not highly specific indicators of folate status because they can be influenced by other factors, including renal dysfunction and deficiencies of vitamin B12 and other micronutrients.^32–34,36^ Folate is naturally found in a variety of foods, including vegetables (especially dark green leafy vegetables), fruits, nuts, beans, peas, seafood, eggs, dairy products, meat, poultry, and cereals. Spinach, liver, asparagus, and Brussels sprouts are particularly rich in folate. Homocysteine levels and certain polymorphisms in the methylenetetrahydrofolate reductase (MTHFR) gene, which can lead to reduced enzyme activity and elevated homocysteine levels, have been studied extensively. These factors have been also studied in relation to psychiatric disorders. There is increasing evidence that MTHFR C677T and A1298C polymorphisms are associated with psychiatric disorders, suggesting potential targets for personalized treatment management.^37^ In addition, several studies have investigated the efficacy of FA and its derivative, L-methylfolate, which can cross the blood-brain barrier. These have been considered as monotherapy or adjunctive therapies for depression, particularly in individuals with low serum and red blood cell folate levels.^38^

**Vitamin B12**

Commonly known as cobalamin, vitamin B12 occurs naturally in some foods and is available as a dietary supplement and prescription medication. Humans are unable to synthesize vitamin B12 and rely entirely on dietary sources. In non-vegetarian diets, the main sources are animal proteins such as meat, fish, eggs, and milk and dairy products.^39^ After absorption, significant amounts of vitamin B12 are stored in the liver, and once saturated, it takes six to 12 years to become deficient.^39,40^ Vitamin B12 status is typically assessed by measuring serum or plasma levels. The cut-off between normal and deficient levels varies by method and laboratory, but most define subnormal serum or plasma levels as less than 200 or 250 pg/mL (148 or 185 pmol/L). Vitamin B12 is essential for the development, myelination, and function of the central nervous system, healthy red blood cell formation, and DNA synthesis. It also acts as a cofactor for two enzymes that are essential for DNA, RNA, proteins, and lipids. A recent systematic review reported lower vitamin B12 intake in participants with ASD, but not in patients with MDD, compared with a control group. In addition, higher vitamin B12 intake was associated with a lower risk of depression and conduct problems in children and adolescents.^41^

**Vitamin E**

Alpha-tocopherol, the orally bioavailable alpha form of the fat-soluble vitamin E, serves as a fat-soluble antioxidant that inhibits the production of ROS generated during fat oxidation. In addition to its role as an antioxidant, vitamin E plays a critical role in immune function, cell signalling, regulation of gene expression, and various metabolic processes.^42^ It is found in vegetable oils, vegetable oil-based fat spreads, nuts and seeds, some fatty fish, egg yolks, and whole grains. The natural form of alpha-tocopherol, known as RRR-α-tocopherol (commonly referred to as d-alpha-tocopherol), is found in plants. In contrast, the synthetically produced form is a mixture of all eight possible stereoisomers (RRR, RSR, RRS, RSS, SSS, SRS, SSR, and SRR) with the same antioxidant activity (commonly referred to as dl-alpha-tocopherol).^43^ According to the European Food Safety Authority (EFSA), the average intake of alpha-tocopherol in adults (≥18 years) in EU countries is 7.8 to 12.5 mg/day for women and 8.2 to 16 mg/day for men. Alpha-tocopherol helps maintain the integrity of cell membranes and protects cells from damage by preventing the oxidation of proteins and polyunsaturated fatty acids (PUFAs) in membrane phospholipids and plasma lipoproteins. It also inhibits lipid peroxidation. Interestingly, alpha-tocopherol treatment has been shown to reverse increased lipid peroxidation, iron accumulation, and altered mitochondrial membrane potential in neuronal cell lines.^44^ Given the association between inflammation and neuropsychiatric disorders, the therapeutic potential of anti-inflammatory treatments, including vitamin E, has received increasing attention. However, clear data from RCTs are still lacking, and indications remain mainly limited to preclinical research.^45^

**Vitamin A**

Vitamin A includes the fat-soluble retinoids, primary retinol and retinyl esters. All-trans retinol and its esters, along with provitamin A (β-carotene), are important dietary sources of retinol. Foods rich in retinol include organ meats, butter, retinol-enriched margarine, dairy products, and eggs. On the other hand, β-carotene-rich foods include fruits and vegetables such as sweet potatoes, carrots, squash, dark green leafy vegetables, sweet red peppers, mangoes, and melons.^46^ Vitamin A plays a critical role in vision as an essential component of rhodopsin, which is central to the mechanisms of phototransduction. It is also essential for the systemic maintenance of cell growth and integrity in body tissues through the action of retinoic acid, which serves as a regulator of genomic expression. Vitamin A is also involved in immune function, cellular communication, growth and development, and male and female reproduction.^47,48^ Assessment of vitamin A status is most effectively expressed in terms of total body stores of retinol (free retinol and retinyl esters) or, alternatively, liver concentration of the vitamin. A concentration of 20 μg retinol/g liver (0.07 μmol/g) is considered sufficient for adults to maintain adequate plasma retinol concentrations. The reference intakes (PRIs) established by EFSA round to 750 μg RE/day for men and 650 μg RE/day for women.^49^ Several studies have examined vitamin A, showing reduced serum retinoic acid levels as a risk factor for developing post-stroke depression, reduced retinol levels in children with ASD, reduced retinoic acid and retinol in patients with SCZ, and higher retinol in MDD.^50^

**Vitamin C**

Also known as L-ascorbic acid, serves as an enzyme cofactor for biochemical reactions catalysed by monooxygenases, dioxygenases, and mixed-function oxygenases. It plays a key role in the biosynthesis of collagen, the synthesis of carnitine and catecholamines, and is essential for the metabolism of cholesterol to bile acids. In addition, vitamin C is essential for the prevention of scurvy in humans and plays a role in the primary prevention of common and complex diseases such as coronary heart disease, stroke, and cancer. Because humans, unlike most animals, are unable to synthesize vitamin C endogenously, it is an essential component of the diet.^51^ The primary sources of vitamin C for adults are fruits, vegetables, and potatoes. Epidemiological evidence suggests a 5% prevalence of vitamin C deficiency and a 13% prevalence of suboptimal status, even in developed countries.^52^ Recently, vitamin C deficiency was found to be as highly prevalent in an inpatient psychiatric setting and persistent even in the presence of a favourable risk factor profile.^53^

**Vitamin B3**

Commonly known as niacin, this term refers specifically to nicotinic acid (NA, pyridine-3-carboxylic acid), but it is often used to refer to a group of chemicals related to niacin, such as nicotinamide (pyridine-3-carboxamide). Synthesized by plants, fungi, and bacteria, rich sources of vitamin B3 include peanuts, fish, mushrooms, yeast, legumes, nuts, and milk, cheese, and eggs, which contain proteins with tryptophan.^54^ Humans can partially convert the essential amino acid tryptophan to nicotinamide in the liver, making tryptophan a dietary source of vitamin B3.^54,55^ In cells, vitamin B3 is essential for the synthesis of nicotinamide adenine dinucleotide (NAD+), a widely distributed metabolite that is essential for mitochondrial metabolism, redox homeostasis, DNA repair, RNA processing, and epigenetic pathways.^56,57^ For the past 50 years, high doses of vitamin B3 have been used to treat people with hypercholesterolemia. It has also been shown to be effective in improving the symptoms of patients with mitochondrial myopathy by restoring muscle and blood NAD+ levels, thereby increasing muscle strength.^57^ Nicotinamide has been shown to increase the intracellular pool of NAD+ while enhancing the expression of mitochondrial proteins and mitochondrial respiration-dependent genes in the retinal pigment epithelial cell line.^56^ Vitamin B3 has long been recognized as a key mediator of neuronal development and survival; however, conflicting results have been reported regarding vitamin B3 dysmetabolism in neuropsychiatric disorders.^58^

**Vitamin B1**

Also known as thiamine (or thiamin), is a water-soluble B vitamin that plays a critical role in energy metabolism and influences cell growth, development, and function. Absorbed from the small intestine by active transport at the dietary level and by passive diffusion at higher pharmacological doses, it is obtained from several sources, including whole grains, meat, and fish.^59^ Vitamin B1 is fortified in breads, cereals, and infant formulas in the United States and many other countries. However, heating and throwing away foods containing vitamin B1 can reduce its content, and because it is soluble in water, a significant amount is lost when cooking water is discarded. Humans store small amounts of vitamin B1 primarily in the liver, and because it has a short half-life, a continuous supply from the diet is necessary.^60^ Approximately 80% of vitamin B1 in the adult human body (about 25-30 mg) is in the form of thiamine diphosphate (TDP; also called thiamine pyrophosphate), the primary metabolically active form. Bacteria present in the large intestine can synthesize free vitamin B1 and TDP, but it is currently unknown whether their contribution plays a role in vitamin B1 nutrition.^61^ TDP serves as an essential cofactor for five enzymes involved in glucose, amino acid, and lipid metabolism. Vitamin B1 status is often measured indirectly by assaying the activity of the TDP-dependent enzyme transketolase in erythrocyte hemolysates in the presence and absence of added TDP.^60^ Another commonly used measure is urinary excretion, which provides information on dietary intake but not tissue reserves.^62^ The primary symptoms of vitamin B1 deficiency are classified as neuronal dysfunction. It is used in preventive and therapeutic interventions for Wernicke-Korsakoff syndrome in alcohol abusers, particularly in neurology and psychiatry.^63^

**Vitamin B2**

Also known as riboflavin, is a critical component of two important coenzymes: flavin adenine dinucleotide (FAD) and flavin mononucleotide (FMN), also known as riboflavin 5’-phosphate. These coenzymes play key roles in several bodily processes, including energy production; cell function and growth; metabolism of fats, drugs, and steroids; and the conversion of the amino acid tryptophan to niacin (sometimes called vitamin B3), a process that requires FAD.^18,64,65^ Vitamin B2 also helps maintain normal homocysteine levels. Foods rich in vitamin B2 include eggs, organ meats (liver and kidney), milk, and lean meats. In the United States and many other countries, some vegetables, grains, and cereals are fortified with vitamin B2.^66^ Because of its solubility in water, about half of the vitamin B2 content of foods is lost during cooking, while steaming or microwaving results in less loss. Most vitamin B2 is absorbed in the proximal small intestine, and small amounts are stored in the liver, heart, and kidneys.^67^ Excess amounts are either not absorbed or are excreted in the urine.^18^ Bacteria in the large intestine produce vitamin B2, which is absorbed in varying amounts depending on the diet. Research suggests that the production of vitamin B2 is higher after eating plant-based foods compared to meat-based foods ^65^. The relationship between vitamin B2 and mental health has been studied along with other B vitamins, but data on its potential benefits are limited.^68–70^

**Vitamin B5**

Commonly known as pantothenic acid, vitamin B5 is found in a variety of foods, including meat, liver and kidney, eggs, milk, cheese, nuts, mushrooms, yeast, whole grains, legumes, cruciferous vegetables, avocado, potatoes, and tomatoes.^54^. This essential vitamin serves as a precursor for the synthesis of both coenzyme A (CoA) and the acyl carrier protein (ACP).^71^ CoA is critical for the synthesis of fatty acids, cholesterol, acetylcholine, and plays an essential role in the metabolism of major organic molecules such as lipids, carbohydrates, and proteins. At the same time, ACP is essential for the synthesis of fatty acids.^54^ Vitamin B5 deficiency is a rare condition associated with malnutrition and presents with symptoms such as fatigue, headache, malaise, personality changes, numbness, muscle cramps, paraesthesia, abdominal/muscle cramps, nausea, and poor muscle coordination.^72^ Recently, vitamin B5 has been proposed as a potential biomarker for BD to distinguish it from MDD.^73^

References

1. Ross AC, Manson JAE, Abrams SA, Aloia JF, Brannon PM, Clinton SK, *et al*. The 2011 report on dietary reference intakes for calcium and vitamin D from the Institute of Medicine: what clinicians need to know. *J Clin Endocrinol Metab*. 2011;96(1):53-58.

2. Copper. PJR. Present Knowledge in Nutrition. 10th ed: John Wiley & Sons, Inc.; Published online 2012:434-446. Accessed April 8, 2024. https://www.wiley.com/en-us/Present+Knowledge+in+Nutrition%2C+10th+Edition-p-9781119946045

3. Guirgis H, Duchemin AM, Vargo S, Youssef NA. Vitamin D levels among adult psychiatric inpatients and the association with psychosis. *Ann Clin Psychiatry*. 2023;35(4):238-245.

4. Mokhtari V, Afsharian P, Shahhoseini M, Kalantar SM, Moini A. A review on various uses of N-acetyl cysteine. *Cell J*. 2017;19(1):11-17.

5. Anton R, Barlow S, Boskou D, Castle L, Crebelli R, Dekant W, et al. Opinion of the Scientific Panel on Food Additives, Flavourings, Processing Aids and Materials in Contact with Food (AFC) on a request from the Commission related to N-Acetyl-L-cysteine for use in foods for particular nutritional uses and in foods for special medical purposes. *EFSA Journal*. 2003;21:1-8.

6. Bradlow RCJ, Berk M, Kalivas PW, Back SE, Kanaan RA. The Potential of N-Acetyl-L-Cysteine (NAC) in the Treatment of Psychiatric Disorders. *CNS Drugs*. 2022;36(5):451-482.

7. Ferreira GC, McKenna MC. L-Carnitine and Acetyl-L-carnitine Roles and Neuroprotection in Developing Brain. *Neurochem Res*. 2017;42(6):1661-1675.

8. Veronese N, Stubbs B, Solmi M, Ajnakina O, Carvalho AF, Maggi S. Acetyl-L-Carnitine Supplementation and the Treatment of Depressive Symptoms: A Systematic Review and Meta-Analysis. *Psychosom Med*. 2018;80(2):154-159.

9. Sangsefidi ZS, Yaghoubi F, Hajiahmadi S, Hosseinzadeh M. The effect of coenzyme Q10 supplementation on oxidative stress: A systematic review and meta-analysis of randomized controlled clinical trials. *Food Sci Nutr*. 2020;8(4):1766-1776.

10. Mousavinejad E, Ghaffari MA, Riahi F, Hajmohammadi M, Tiznobeyk Z, Mousavinejad M. Coenzyme Q10 supplementation reduces oxidative stress and decreases antioxidant enzyme activity in children with autism spectrum disorders. *Psychiatry Res*. 2018;265:62-69.

11. Maguire Á, Hargreaves A, Gill M. Coenzyme Q10 and neuropsychiatric and neurological disorders: relevance for schizophrenia. *Nutr Neurosci*. 2020;23(10):756-769.

12. Najafi N, Mehri S, Ghasemzadeh Rahbardar M, Hosseinzadeh H. Effects of alpha lipoic acid on metabolic syndrome: A comprehensive review. *Phytother Res*. 2022;36(6):2300-2323.

13. Vidović B, Milovanović S, Stefanović A, Kotur-Stevuljević J, Takić M, Debeljak-Martačić J, *et al*. Effects of Alpha-Lipoic Acid Supplementation on Plasma Adiponectin Levels and Some Metabolic Risk Factors in Patients with Schizophrenia. *J Med Food*. 2017;20(1):79-85.

14. Ratliff JC, Palmese LB, Reutenauer EL, Tek C. An open-label pilot trial of alpha-lipoic acid for weight loss in patients with schizophrenia without diabetes. *Clin Schizophr Relat Psychoses*. 2015;8(4):196-200.

15. Sanders LLO, De Souza Menezes CE, Chaves Filho AJM, De Almeida Viana G, Fechine FV, *et al*. α-Lipoic Acid as Adjunctive Treatment for Schizophrenia: An Open-Label Trial. *J Clin Psychopharmacol*. 2017;37(6):697-701.

16. Botturi A, Ciappolino V, Delvecchio G, Boscutti A, Viscardi B, Brambilla P. The role and the effect of magnesium in mental disorders: A systematic review. *Nutrients*. 2020;12(6):1661.

17. Jahnen-Dechent W, Ketteler M. Magnesium basics. *Clin Kidney J*. 2012;5(Suppl 1):i3-i14.

18. Institute of Medicine (US) Standing Committee on the Scientific Evaluation of Dietary Reference Intakes and its Panel on Folate, Other B Vitamins, and Choline. *Dietary Reference Intakes for Thiamin, Riboflavin, Niacin, Vitamin B6, Folate, Vitamin B12, Pantothenic Acid, Biotin, and Choline.* (The National Academies Press, ed.). 1998.

19. McCormick D. Vitamin B6. In: Bowman BA, Russell RM, eds. *Present Knowledge in Nutrition*. Vol 1. International Life Sciences Institute; 2006:269-277.

20. Mackey AD, David SR, Gregory JF. Vitamin B6. In: Shils M, Shike M, Ross A, Caballero B, Cousins R, eds. *Modern Nutrition in Health and Disease*. 10th ed. Williams & Wilkins; 2006.

21. McCormick DB. Riboflavin. In: Erdman JW, Macdonald IA, Zeisel SH, eds. *Present Knowledge in Nutrition*. 10th ed. Wiley-Blackwell; 2012:280-292.

22. Durrani D, Idrees R, Idrees H, Ellahi A. Vitamin B6: A new approach to lowering anxiety, and depression? *Ann Med Surg (Lond)*. 2022;82: 104663.

23. Pacheco-Alvarez D, Solórzano-Vargas RS, Del Río AL. Biotin in metabolism and its relationship to human disease. *Arch Med Res*. 2002;33(5):439-447.

24. Staggs CG, Sealey WM, McCabe BJ, Teague AM, Mock DM. Determination of the biotin content of select foods using accurate and sensitive HPLC/avidin binding. *J Food Compost Anal*. 2004;17(6):767-776.

25. Zempleni J. Biotin. In: Erdman JW, Macdonald IA, Zeisel SH, eds. *Present Knowledge in Nutrition*. 10th ed. Wiley-Blackwell; 2012:359-374.

26. Mock DM. Biotin. In: Ross AC, Caballero B, Cousins RJ, Tucker KL, Ziegler TR, eds. *Modern Nutrition in Health and Disease*. 11th ed. Lippincott Williams & Wilkins; 2014:390-398.

27. Zempleni J, Wijeratne SSK, Hassan YI. Biotin. *Biofactors*. 2009;35(1):36-46.

28. Said HM. Biotin: biochemical, physiological and clinical aspects. *Subcell Biochem*. 2012;56:1-19.

29. Mock DM, Stratton SL, Horvath TD, Bogusiewicz A, Matthews NI, Henrich CL, *et al*. Urinary excretion of 3-hydroxyisovaleric acid and 3-hydroxyisovaleryl carnitine increases in response to a leucine challenge in marginally biotin-deficient humans. *J Nutr*. 2011;141(11):1925-1930.

30. Eng WK, Giraud D, Schlegel VL, Wang D, Lee BH, Zempleni J. Identification and assessment of markers of biotin status in healthy adults. *Br J Nutr*. 2013;110(2):321-329.

31. de la Bâtie CD, Barbier V, Roda C, Brassier A, Arnoux JB, Valayannopoulos V, *et al*. Autism spectrum disorders in propionic acidemia patients. *J Inherit Metab Dis*. 2018;41(4):623-629.

32. Bailey LB CM. Folate. In: Erdman JW, Macdonald IA, Zeisel SH, eds. *Present Knowledge in Nutrition*. 10th ed. Wiley-Blackwell; 2012:321-342.

33. Bailey LB, Stover PJ, McNulty H, Fenech MF, Gregory JF, Mills JL, *et al*. Biomarkers of Nutrition for Development-Folate Review. *J Nutr*. 2015;145(7):1636S-1680S.

34. Stover PJ. Folic acid. In: Ross AC, Caballero B, Cousins RJ, Tucker KL, Ziegler TR, eds. *Modern Nutrition in Health and Disease*. 11th ed. Lippincott Williams & Wilkins; 2012:358-368.

35. Yetley EA, Pfeiffer CM, Phinney KW, Fazili Z, Lacher DA, Bailey RL, *et al*. Biomarkers of folate status in NHANES: a roundtable summary. *Am J Clin Nutr*. 2011;94(1):303S-312S.

36. Green R. Indicators for assessing folate and vitamin B-12 status and for monitoring the efficacy of intervention strategies. *Am J Clin Nutr*. 2011;94(2): 666S-72S.

37. Zhang YX, Yang LP, Gai C, Cheng CC, Guo ZY, Sun HM, *et al*. Association between variants of MTHFR genes and psychiatric disorders: A meta-analysis. *Front Psychiatry*. 2022;13: 976428.

38. Liwinski T, Lang UE. Folate and Its Significance in Depressive Disorders and Suicidality: A Comprehensive Narrative Review. *Nutrients*. 2023;15(17): 3859.

39. Romain M, Sviri S, Linton DM, Stav I, Van Heerden P V. The role of Vitamin B12 in the critically ill - A review. *Anaesth Intensive Care*. 2016;44(4):447-452.

40. Shipton MJ, Thachil J. Vitamin B12 deficiency - A 21st century perspective. *Clin Med (Lond)*. 2015;15(2):145-150.

41. Tan Y, Zhou L, Gu K, Xie C, Wang Y, Cha L, *et al*. Correlation between Vitamin B12 and Mental Health in Children and Adolescents: A Systematic Review and Meta-analysis. *Clin Psychopharmacol Neurosci*. 2023;21(4):617-633.

42. Traber MG. Vitamin E. In: Shils ME, SM, RAC, CB and CRJ, ed. *Modern Nutrition in Health and Disease*. Lippincott, Williams and Wilkins; 2006:396-411.

43. Lauridsen C, Jensen SK. α-Tocopherol incorporation in mitochondria and microsomes upon supranutritional vitamin E supplementation. *Genes Nutr*. 2012;7(4):475-482.

44. Villalón-García I, Álvarez-Córdoba M, Povea-Cabello S, Talaverón-Rey M, Villanueva-Paz M, Luzón-Hidalgo R, *et al*. Vitamin E prevents lipid peroxidation and iron accumulation in PLA2G6-Associated Neurodegeneration. *Neurobiol Dis*. 2022;165:105649.

45. Muscaritoli M. The Impact of Nutrients on Mental Health and Well-Being: Insights From the Literature. *Front Nutr*. 2021;8:656290.

46. Dawson MI. The importance of vitamin A in nutrition. *Curr Pharm Des*. 2000;6(3):311-325.

47. Blaner WS. Vitamin A and provitamin A carotenoids. *Present Knowledge in Nutrition*. Published online January 1, 2020:73-91. doi:10.1016/B978-0-323-66162-1.00005-6

48. Webb G. Modern Nutrition in Health and Disease, 9th edition. M. E. Shils, J. A. Olsen, M. Shike and A. C. Ross (editors). 1999. Baltimore: Williams &amp; Wilkins.

49. Agostoni C, Canani RB, Fairweather-Tait S, Heinonen M, Korhonen H, La Vieille S, et al. Scientific opinion on dietary reference values for Vitamin A. *EFSA Journal*. 13(3):4028.

50. Otto LR, Clemens V, Üsekes B, Cosma NC, Regen F, Hellmann-Regen J. Retinoid homeostasis in major depressive disorder. *Transl Psychiatry*. 2023;13(1):67.

51. Li Y, Schellhorn HE. New developments and novel therapeutic perspectives for vitamin C. *J Nutr*. 2007;137(10):2171-2184.

52. Granger M, Eck P. Dietary Vitamin C in Human Health. *Adv Food Nutr Res*. 2018;83:281-310.

53. Bari BA, Ivkovic A, Wininger BA. Mild Vitamin C Deficiency Is Common in the Inpatient Psychiatric Setting. *J Clin Psychiatry*. 2023;84(4):22m14616.

54. Hrubša M, Siatka T, Nejmanová I, Vopršalová M, Krčmová LK, Matoušová K, *et al*. Biological Properties of Vitamins of the B-Complex, Part 1: Vitamins B1, B2, B3, and B5. *Nutrients*. 2022;14(3):484.

55. Xu XJ, Jiang GS. Niacin-respondent subset of schizophrenia – a therapeutic review. *Eur Rev Med Pharmacol Sci*. 2015;19(6):988-997.

56. Hazim RA, Paniagua AE, Tang L, Yang K, Kim KKO, Stiles L, *et al*. Vitamin B3, nicotinamide, enhances mitochondrial metabolism to promote differentiation of the retinal pigment epithelium. *J Biol Chem*. 2022;298(9):102286.

57. Pirinen E, Auranen M, Khan NA, Brilhante V, Urho N, Pessia A, *et al*. Niacin Cures Systemic NAD+ Deficiency and Improves Muscle Performance in Adult-Onset Mitochondrial Myopathy. *Cell Metab*. 2020;32(1):144.

58. Gasperi V, Sibilano M, Savini I, Catani MV. Niacin in the Central Nervous System: An Update of Biological Aspects and Clinical Applications. *Int J Mol Sci*. 2019;20(4):974.

59. Said HM. Thiamin. In: Coates PM, Betz JM, Blackman MR, *et al*., eds. *Encyclopedia of Dietary Supplements*. 2nd ed. Informa Healthcare; 2010:748-753.

60. Bemeur C BR. Thiamin. In: Ross AC, Caballero B, Cousins RJ, Tucker KL, Ziegler TR, eds. *Modern Nutrition in Health and Disease*. 11th ed. Lippincott Williams & Wilkins; 2014:317-324.

61. Nabokina SM, Said HM. A high-affinity and specific carrier-mediated mechanism for uptake of thiamine pyrophosphate by human colonic epithelial cells. *Am J Physiol Gastrointest Liver Physiol*. 2012;303(3):G389-395.

62. Allen L, Benoist B de, Dary O, Hurrell R. Guidelines on Food Fortification With Micronutrients. *WHO, FAO*. https://cdn.who.int/media/docs/default-source/micronutrients/gff-contents-en.pdf?sfvrsn=f964afe1_2

63. Aquilina G, Bories G, Chesson A, Sandro Cocconcelli P, de Knecht J, Dierick N, *et al*. Scientific Opinion on the safety and efficacy of vitamin B1 (thiamine mononitrate and thiamine hydrochloride) as a feed additive for all animal species based on a dossier submitted by Lohmann Animal Health. *EFSA Journal*. 2011;9(11):2411.

64. Rivlin RS. Riboflavin. In: Coates PM, Betz JM, Blackman MR, eds. *Encyclopedia of Dietary Supplements*. 2nd ed. Informa Healthcare; 2010:691-699.

65. Said HM RA. Riboflavin. In: Ross AC, Caballero B, Cousins RJ, Tucker KL, Ziegler TR, eds. *Modern Nutrition in Health and Disease*. 11th ed. Lippincott Williams & Wilkins; 2014:325-330.

66. World Health Organization. Essential medicines for mental disorders. In: *Pharmacological Treatment of Mental Disorders in Primary Health Care*. Chapter 1. 2009.

67. McCormick DB. Vitamin/mineral supplements: of questionable benefit for the general population. *Nutr Rev*. 2010;68(4):207-213.

68. Rouhani P, Amoushahi M, Keshteli AH, Saneei P, Afshar H, Esmaillzadeh A, *et al*. Dietary riboflavin intake in relation to psychological disorders in Iranian adults: an observational study. *Sci Rep*. 2023;13(1):5152.

69. Murakami K, Miyake Y, Sasaki S, Tanaka K, Arakawa M. Dietary folate, riboflavin, vitamin B-6, and vitamin B-12 and depressive symptoms in early adolescence: the Ryukyus Child Health Study. *Psychosom Med*. 2010;72(8):763-768.

70. Murakami K, Mizoue T, Sasaki S, Ohta M, Sato M, Matsushita Y, *et al*. Dietary intake of folate, other B vitamins, and omega-3 polyunsaturated fatty acids in relation to depressive symptoms in Japanese adults. *Nutrition*. 2008;24(2):140-147.

71. Gheita AA, Gheita TA, Kenawy SA. The potential role of B5: A stitch in time and switch in cytokine. *Phytother Res*. 2020;34(2):306-314.

72. Sanvictores T, Chauhan S. *Vitamin B5 (Pantothenic Acid)*. In: StatPearls [Internet]. Treasure Island (FL): StatPearls Publishing; 2026.

73. Sun XL, Ma LN, Chen ZZ, Xiong YB, Jia J, Wang Y, *et al*. Search for serum biomarkers in patients with bipolar disorder and major depressive disorder using metabolome analysis. *Front Psychiatry*. 2023;14:1251955.

**Supplementary figure 1: Effect of nutraceutical supplements on key biological processes in mitochondria.**


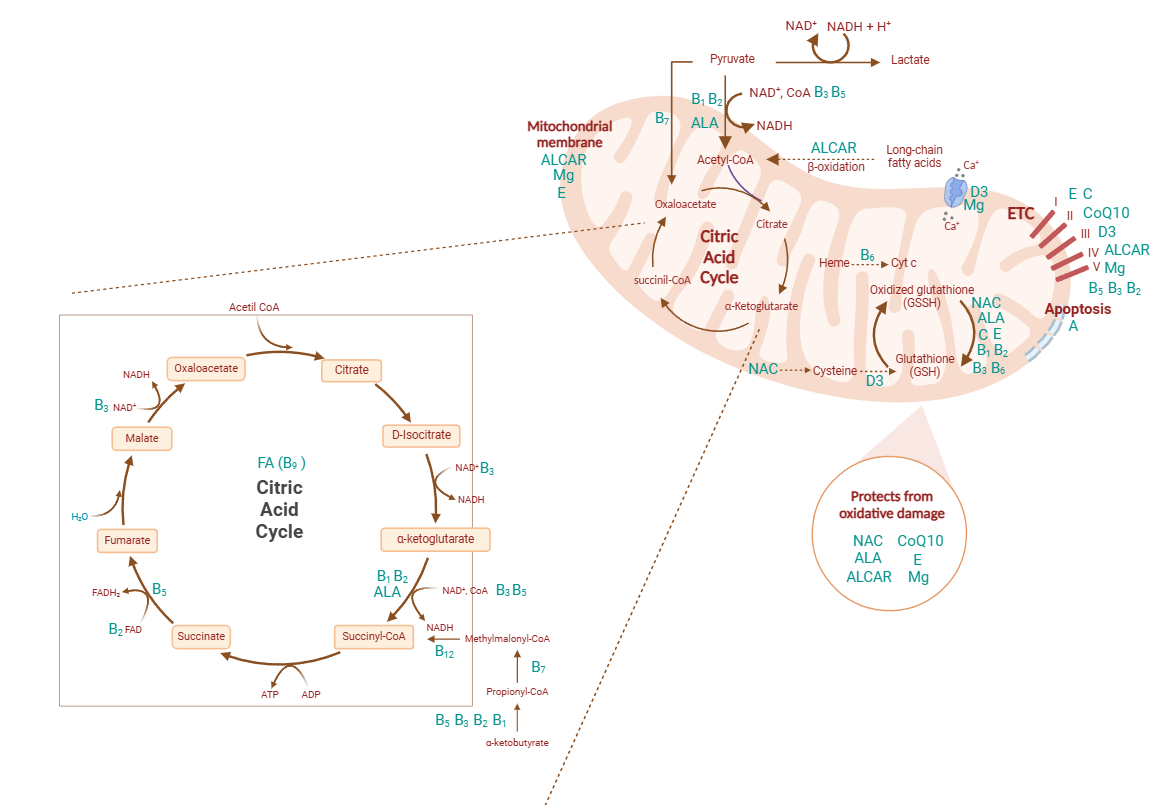


**Supplementary Figure 1.** **Schematic representation of mitochondria and the effect of nutraceutical supplements on key biochemical processes.**

This diagram shows how various compounds, including B-complex vitamins (B₁, B₂, B₃, B₅, B₆, B₇, B₉, B₁₂), antioxidants (NAC, ALA, ALCAR, CoQ10, Vitamin E, Vitamin C), and the mineral magnesium (Mg) interact with and support mitochondrial metabolic pathways. These pathways include the citric acid cycle, β-oxidation, and the electron transport chain (ETC). Additionally, these compounds contribute to the regulation of apoptosis, stabilization of the mitochondrial membrane, and protection against oxidative stress, thereby preventing mitochondrial dysfunction and cellular damage.

**2. References of the 122 reports that met the inclusion criteria and are included in Tables 1–4.**

1. Vyas CM, Mischoulon D, Chang G, Cook NR, Weinberg A, Copeland T, et al. Effects of Vitamin D3 and Marine Omega-3 Fatty Acids Supplementation on Indicated and Selective Prevention of Depression in Older Adults: Results From the Clinical Center Sub-Cohort of the VITamin D and OmegA-3 TriaL (VITAL). ***J Clin Psychiatry***. 2023;84(4): 22m14629.

2. Rahman ST, Waterhouse M, Romero BD, Baxter C, English DR, Almeida OP, et al. Effect of vitamin D supplementation on depression in older Australian adults. ***Int J Geriatr Psychiatry***. 2023;38(1):e5847.

3. Samadi M, Gholami F, Seyedi M, Jalali M, Effatpanah M, Yekaninejad MS, et al. Effect of Vitamin D Supplementation on Inflammatory Biomarkers in School-Aged Children with Attention Deficit Hyperactivity Disorder. ***Int J Clin Pract***. 2022;2022:1256408.

4. Kumar PNS, Menon V, Andrade C. A randomized, double-blind, placebo-controlled, 12-week trial of vitamin D augmentation in major depressive disorder associated with vitamin D deficiency. ***J Affect Disord***. 2022;314:143-149.

5. Mohammadzadeh Honarvar N, Samadi M, Seyedi Chimeh M, Gholami F, Bahrampour N, Jalali M, et al. Effect of Vitamin D on Paraxonase-1, Total Antioxidant Capacity, and 8-Isoprostan in Children with Attention Deficit Hyperactivity Disorder. ***Int J Clin Pract***. 2022;2022:4836731.

6. Amini S, Amani R, Jafarirad S, Cheraghian B, Sayyah M, Hemmati AA. The effect of vitamin D and calcium supplementation on inflammatory biomarkers, estradiol levels and severity of symptoms in women with postpartum depression: a randomized double-blind clinical trial. ***Nutr Neurosci***. 2022;25(1):22-32.

7. Gaughran F, Stringer D, Wojewodka G, Landau S, Smith S, Gardner-Sood P, et al. Effect of Vitamin D Supplementation on Outcomes in People with Early Psychosis: The DFEND Randomized Clinical Trial. ***JAMA Netw Open***. 2021;4(12): e2140858.

8. Okereke OI, Reynolds 3rd CF, Mischoulon D, Chang G, Vyas CM, Cook NR, et al. Effect of Long-term Vitamin D3 Supplementation vs Placebo on Risk of Depression or Clinically Relevant Depressive Symptoms and on Change in Mood Scores: A Randomized Clinical Trial. ***JAMA***. 2020;324(5):471-480.

9. Libuda L, Timmesfeld N, Antel J, Hirtz R, Bauer J, Führer D, et al. Effect of vitamin D deficiency on depressive symptoms in child and adolescent psychiatric patients: results of a randomized controlled trial. ***Eur J Nutr***. 2020;59(8):3415-3424.

10. Feng JY, Li HH, Wang B, Shan L, Jia FY. Successive clinical application of vitamin D and bumetanide in children with autism spectrum disorder: A case report. ***Medicine (Baltimore)***. 2020;99(2):e18661.

11. Gaughran F, Stringer D, Berk M, Smith S, Taylor D, Whiskey E, et al. Vitamin D supplementation compared to placebo in people with First Episode psychosis - Neuroprotection Design (DFEND): a protocol for a randomised, double-blind, placebo-controlled, parallel-group trial. ***Trials***. 2020;21(1):14.

12. Alghamdi S, Alsulami N, Khoja S, Alsufiani H, Tayeb HO, Tarazi FI. Vitamin D Supplementation Ameliorates Severity of Major Depressive Disorder. ***J Mol Neurosci***. 2020;70(2):230-235.

13. Infante M, Sears B, Rizzo AM, Mariani Cerati D, Caprio M, Ricordi C, et al. Omega-3 PUFAs and vitamin D co-supplementation as a safe-effective therapeutic approach for core symptoms of autism spectrum disorder: case report and literature review. ***Nutr Neurosci***. 2020;23(10):779-790.

14. Kaviani M, Nikooyeh B, Zand H, Yaghmaei P, Neyestani TR. Effects of vitamin D supplementation on depression and some involved neurotransmitters. ***J Affect Disord***. 2020;269:28-35.

15. Hansen JP, Pareek M, Hvolby A, Schmedes A, Toft T, Dahls E, et al. Vitamin D3 supplementation and treatment outcomes in patients with depression (D3-vit-dep). ***BMC Res Notes***. 2019;12(1):203.

16. Ghaderi A, Banafshe HR, Mirhosseini N, Moradi M, Karimi MA, Mehrzad F, et al. Clinical and metabolic response to vitamin D plus probiotic in schizophrenia patients. ***BMC Psychiatry***. 2019;19(1):77.

17. Kerley CP, Elnazir B, Greally P, Coghlan D. Blunted serum 25(OH)D response to vitamin D3supplementation in children with autism. ***Nutr Neurosci***. 2020;23(7):537-542.

18. Bahrami A, Mazloum SR, Maghsoudi S, Soleimani D, Khayyatzadeh SS, Arekhi S, et al. High Dose Vitamin D Supplementation Is Associated With a Reduction in Depression Score Among Adolescent Girls: A Nine-Week Follow-Up Study. ***J Diet Suppl***. 2018;15(2):173-182.

19. Mazahery H, Conlon CA, Beck KL, Mugridge O, Kruger MC, Stonehouse W, et al. A Randomised-Controlled Trial of Vitamin D and Omega-3 Long Chain Polyunsaturated Fatty Acids in the Treatment of Core Symptoms of Autism Spectrum Disorder in Children. ***J Autism Dev Disord***. 2019;49(5):1778-1794.

20. Mohammadpour N, Jazayeri S, Tehrani-Doost M, Djalali M, Hosseini M, Effatpanah M, et al. Effect of vitamin D supplementation as adjunctive therapy to methylphenidate on ADHD symptoms: A randomized, double blind, placebo-controlled trial. ***Nutr Neurosci***. 2018;21(3):202-209.

21. Mousa A, Naderpoor N, de Courten MPJ, de Courten B. Vitamin D and symptoms of depression in overweight or obese adults: A cross-sectional study and randomized placebo-controlled trial. ***J Steroid Biochem Mol Biol***. 2018;177:200-208.

22. Föcker M, Antel J, Grasemann C, Führer D, Timmesfeld N, Öztürk D, et al. Effect of an vitamin D deficiency on depressive symptoms in child and adolescent psychiatric patients - a randomized controlled trial: study protocol. ***BMC Psychiatry***. 2018;18(1):57. doi:10.1186/s12888-018-1637-7

23. Krivoy A, Onn R, Vilner Y, Hochman E, Weizman S, Paz A, et al. Vitamin D Supplementation in Chronic Schizophrenia Patients Treated with Clozapine: A Randomized, Double-Blind, Placebo-controlled Clinical Trial. ***EBioMedicine***. 2017;26:138-145.

24. Kerley CP, Power C, Gallagher L, Coghlan D. Lack of effect of vitamin D3supplementation in autism: a 20-week, placebo-controlled RCT. ***Arch Dis Child***. 2017;102(11):1030-1036.

25. Marsh WK, Penny JL, Rothschild AJ. Vitamin D supplementation in bipolar depression: A double blind placebo controlled trial. ***J Psychiatr Res***. 2017;95:48-53.

26. Feng J, Shan L, Du L, Wang B, Li H, Wang W, et al. Clinical improvement following vitamin D3 supplementation in Autism Spectrum Disorder. ***Nutr Neurosci***. 2017;20(5):284-290.

27. Vaziri F, Nasiri S, Tavana Z, Dabbaghmanesh MH, Sharif F, Jafari P. A randomized controlled trial of vitamin D supplementation on perinatal depression: in Iranian pregnant mothers. ***BMC Pregnancy Childbirth***. 2016;16:239.

28. Wang Y, Liu Y, Lian Y, Li N, Liu H, Li G. Efficacy of High-Dose Supplementation With Oral Vitamin D3 on Depressive Symptoms in Dialysis Patients With Vitamin D3 Insufficiency. ***J Clin Psychopharmacol***. 2016;36(3):229-235.

29. Saad K, Abdel-rahman AA, Elserogy YM, Al-Atram AA, Cannell JJ, Bjørklund G, et al. Vitamin D status in autism spectrum disorders and the efficacy of vitamin D supplementation in autistic children. ***Nutr Neurosci***. 2016;19(8):346-351.

30. Sikoglu EM, Navarro AAL, Starr D, Dvir Y, Nwosu BU, Czerniak SM, et al. Vitamin D3 Supplemental Treatment for Mania in Youth with Bipolar Spectrum Disorders. ***J Child Adolesc Psychopharmacol***. 2015;25(5):415-424.

31. Jia F, Wang B, Shan L, Xu Z, Staal WG, Du L. Core symptoms of autism improved after vitamin D supplementation. ***Pediatrics***. 2015;135(1):e196-e198.

32. Snoeijen-Schouwenaars FM, Van Deursen KC, Tan IY, Verschuure P, Majoie MH. Vitamin D supplementation in children with epilepsy and intellectual disability. ***Pediatr Neurol***. 2015;52(2):160-164.

33. Kilpinen-Loisa P, Arvio M, Ilvesmäki V, Mäkitie O. Vitamin D status and optimal supplementation in institutionalized adults with intellectual disability. ***J Intellect Disabil Res***. 2009;53(12):1014-1023.

34. Kanaan RA, Oliver G, Dharan A, Sendi S, Maier A, Mohebbi M, et al. A multi-centre, double-blind, 12-week, randomized, placebo-controlled trial of adjunctive N-Acetylcysteine for treatment-resistant PTSD. ***Psychiatry Res***. 2023;327:115398.

35. Neill E, Rossell SL, Yolland C, Meyer D, Galletly C, Harris A, et al. N-Acetylcysteine (NAC) in Schizophrenia Resistant to Clozapine: A Double-Blind, Randomized, Placebo-Controlled Trial Targeting Negative Symptoms. ***Schizophr Bull***. 2022;48(6):1263-1272.

36. Bortolasci CC, Turner A, Mohebbi M, Liu ZS, Ashton M, Gray L, et al. Baseline serum amino acid levels predict treatment response to augmentation with N-acetylcysteine (NAC) in a bipolar disorder randomised trial. ***J Psychiatr Res***. 2021;142:376-383.

37. Pesko MJ, Burbige EM, Sannar EM, Beresford C, Rogers C, Ariefdjohan M, et al. The use of N-acetylcysteine supplementation to decrease irritability in four youths with autism spectrum disorders. ***J Pediatr Pharmacol Ther***. 2020;25(2):149-154.

38. Ashton MM, Dean OM, Marx W, Mohebbi M, Berk M, Malhi GS, et al. Diet quality, dietary inflammatory index and body mass index as predictors of response to adjunctive N-acetylcysteine and mitochondrial agents in adults with bipolar disorder: A sub-study of a randomised placebo-controlled trial. Aust N Z J Psychiatry. 2020;54(2):159-172.

39. Mullier E, Roine T, Griffa A, Xin L, Baumann PS, Klauser P, et al. N-Acetyl-Cysteine Supplementation Improves Functional Connectivity Within the Cingulate Cortex in Early Psychosis: A Pilot Study. ***Int J Neuropsychopharmacol***. 2019;22(8):478-487.

40. Yang YS, Davis MC, Wynn JK, Hellemann G, Green MF, Marder SR. N-acetylcysteine improves EEG measures of auditory deviance detection and neural synchronization in schizophrenia: A randomized, controlled pilot study. ***Schizophr Res***. 2019;208:479-480.

41. Yang C, Bosker FJ, Li J, Schoevers RA. N-acetylcysteine as add-on to antidepressant medication in therapy refractory major depressive disorder patients with increased inflammatory activity: Study protocol of a double-blind randomized placebo-controlled trial. ***BMC Psychiatry***. 2018;18(1):279.

42. Breier A, Liffick E, Hummer TA, Vohs JL, Yang Z, Mehdiyoun NF, et al. Effects of 12-month, double-blind N-acetyl cysteine on symptoms, cognition and brain morphology in early phase schizophrenia spectrum disorders. Schizophr Res. 2018;199:395-402.

43. Conus P, Seidman LJ, Fournier M, Xin L, Cleusix M, Baumann PS, et al. N-acetylcysteine in a double-blind randomized placebo-controlled trial: Toward biomarker-guided treatment in early psychosis. ***Schizophr Bull***. 2018;44(2):317-327.

44. Sepehrmanesh Z, Heidary M, Akasheh N, Akbari H, Heidary M. Therapeutic effect of adjunctive N-acetyl cysteine (NAC) on symptoms of chronic schizophrenia: A double-blind, randomized clinical trial. ***Prog Neuropsychopharmacol Biol Psychiatry***. 2018;82:289-296.

45. Dean OM, Gray KM, Villagonzalo KA, Dodd S, Mohebbi M, Vick T, et al. A randomised, double blind, placebo-controlled trial of a fixed dose of N-acetyl cysteine in children with autistic disorder. ***Aust N Z J Psychiatry***. 2016;51(3):241-249. doi:10.1177/0004867416652735

46. Wink LK, Adams R, Wang Z, Klaunig JE, Plawacki MH, Posey DJ, et al. A randomized placebo-controlled pilot study of N-acetylcysteine in youth with autism spectrum disorder. ***Mol Autism***. 2016;7:26.

47. Monsivais SR, Wisdom N, Kruse JL, Davis MC. N-Acetylcysteine Supplementation in an Individual With Glucose-6-Phosphate Dehydrogenase Deficiency–Associated Psychosis. ***Biol Psychiatry***. 2016;80(8):e71-e72.

48. Marler S, Sanders KB, Veenstra-Vanderweele J. N-acetylcysteine as treatment for self-injurious behavior in a child with autism. ***J Child Adolesc Psychopharmacol***. 2014;24(4):231-234.

49. Ghanizadeh A, Moghimi-Sarani E. A randomized double blind placebo controlled clinical trial of N-Acetylcysteine added to risperidone for treating autistic disorders. ***BMC Psychiatry***. 2013;13:196.

50. Hardan AY, Fung LK, Libove RA, Obukhanych TV, Nair S, Herzenberg LA, et al. A randomized controlled pilot trial of oral N-acetylcysteine in children with autism. ***Biol Psychiatry***. 2012;71(11):956-961.

51. Rapado-Castro M, Dodd S, Bush AI, Malhi GS, Skvarc DR, On ZX, et al. Cognitive effects of adjunctive N-acetyl cysteine in psychosis. ***Psychol Med***. 2017;47(5):866-876.

52. Berk M, Copolov D, Dean O, Lu K, Jeavons S, Schapkaitz I, et al. N-Acetyl Cysteine as a Glutathione Precursor for Schizophrenia-A Double-Blind, Randomized, Placebo-Controlled Trial. ***Biol Psychiatry***. 2008;64(5):361-368.

53. Ziats MN, Comeaux MS, Yang Y, Scaglia F, Elsea SH, Sun Q, et al. Improvement of regressive autism symptoms in a child with TMLHE deficiency following carnitine supplementation. ***Am J Med Genet A***. 2015;167A(9):2162-2167.

54. Maguire Á, Hargreaves A, Gill M. Coenzyme Q10 and neuropsychiatric and neurological disorders: relevance for schizophrenia. ***Nutr Neurosci***. 2021;23(10):756-769.

55. Jahangard L, Yasrebifar F, Haghighi M, Ranjbar A, Mehrpooya M. Influence of adjuvant Coenzyme Q10 on inflammatory and oxidative stress biomarkers in patients with bipolar disorders during the depressive episode. ***Mol Biol Rep***. 2019;46(5):5333-5343.

56. Mousavinejad E, Ghaffari MA, Riahi F, Hajmohammadi M, Tiznobeyk Z, Mousavinejad M. Coenzyme Q10 supplementation reduces oxidative stress and decreases antioxidant enzyme activity in children with autism spectrum disorders. ***Psychiatry Res***. 2018;265:62-69.

57. Mishra A, Reeta KH, Sarangi SC, Maiti R, Sood M. Effect of add-on alpha lipoic acid on psychopathology in patients with treatment-resistant schizophrenia: a pilot randomized double-blind placebo-controlled trial. ***Psychopharmacology (Berl)***. 2022;239(11):3525-3535.

58. Sanders LLO, de Souza Menezes CE, Chaves Filho AJM, de Almeida Viana G, Fechine FV, Rodrigues de Queiroz MG, et al. α-Lipoic Acid as Adjunctive Treatment for Schizophrenia. ***J Clin Psychopharmacol***. 2017;37(6):697-701.

59. De Lima DN, Filho CWLC, Frota IJ, de Oliveira ALB, de Souza Menezes CE, Filho AJMC, et al. α-Lipoic Acid as Adjunctive Treatment for Schizophrenia: A Randomized Double-Blind Study. ***J Clin Psychopharmacol***. 2023;43(1):39-45.

60. Vidović B, Milovanović S, Stefanović A, Kotur-Stevuljević J, Takić M, Debeljak-Martačić J,et al. Effects of Alpha-Lipoic Acid Supplementation on Plasma Adiponectin Levels and Some Metabolic Risk Factors in Patients with Schizophrenia. ***J Med Food. 2017***;20(1):79-85.

61. Vidović B, Milovanović S, Dordević B, Kotur-Stevuljević J, Stefanović A, Ivanišević J, et al. Effect of alpha-lipoic acid supplementation on oxidative stress markers and antioxidative defense in patients with schizophrenia. ***Psychiatr Danub***. 2014;26(3):205-213.

62. Emsley R, Chiliza B, Asmal L, du Plessis S, Phahladira L, van Niekerk E, et al. A randomized, controlled trial of omega-3 fatty acids plus an antioxidant for relapse prevention after antipsychotic discontinuation in first-episode schizophrenia. ***Schizophr Res***. 2014;158(1-3):230-235.

63. Hemamy M, Pahlavani N, Amanollahi A, Islam SMS, McVicar J, Askari G, et al. The effect of vitamin D and magnesium supplementation on the mental health status of attention-deficit hyperactive children: a randomized controlled trial. ***BMC Pediatr***. 2021;21(1):178.

64. Afsharfar M, Shahraki M, Shakiba M, Asbaghi O, Dashipour A. The effects of magnesium supplementation on serum level of brain derived neurotrophic factor (BDNF) and depression status in patients with depression. ***Clin Nutr ESPEN***. 2021;42:381-386.

65. Debi Ann A, Udayakumar N, Senta C, Rajanandh MG. Pyridoxine and Magnesium Administration–Induced Hyperactivity in Two Children With Autism Spectrum Disorder: Case Reports From a Clinical Trial. ***Clin Ther***. 2020;42(11):e250-e258.

66. Tarleton EK, Littenberg B, MacLean CD, Kennedy AG, Daley C. Role of magnesium supplementation in the treatment of depression: A randomized clinical trial. ***PLoS One***. 2017;12(6):e0180067.

67. Rajizadeh A, Mozaffari-Khosravi H, Yassini-Ardakani M, Dehghani A. Effect of magnesium supplementation on depression status in depressed patients with magnesium deficiency: A randomized, double-blind, placebo-controlled trial. ***Nutrition***. 2017;35:56-60.

68. Rizzo R, Prato A, Scerbo M, Saia F, Barone R, Curatolo P. Use of Nutritional Supplements Based on L-Theanine and Vitamin B6 in Children with Tourette Syndrome, with Anxiety Disorders: A Pilot Study. ***Nutrients***. 2022;14(4):852.

69. Badrfam R, Mostafavi SA, Khaleghi A, Akhondzadeh S, Zandifar A, Farid M, et al. The efficacy of vitamin B6 as an adjunctive therapy to lithium in improving the symptoms of acute mania in patients with bipolar disorder, type 1; a double-blind, randomized, placebo-controlled, clinical trial. ***Brain Behav***. 2021;11(11):e2394.

70. Itokawa M, Miyashita M, Arai M, Dan T, Takahashi K, Tokunaga T, et al. Pyridoxamine: A novel treatment for schizophrenia with enhanced carbonyl stress. ***Psychiatry Clin Neurosci***. 2018;72(1):35-44.

71. Kałużna-Czaplińska J, Michalska M, Rynkowski J. Vitamin supplementation reduces the level of homocysteine in the urine of autistic children. ***Nut Res***. 2011;31(4):318-321.

72. Reininghaus EZ, Platzer M, Kohlhammer-Dohr A, Hamm C, Mörkl S, Bengesser S, et al. PROVIT: Supplementary Probiotic Treatment and Vitamin B7 in Depression-A Randomized Controlled Trial. ***Nutrients***. 2020;12(11):3422.

73. Hosseini SA, Sepehrmanesh Z, Gilasi H, Ghoraishi FS. The Effect of Memantine Versus Folic Acid on Cognitive Impairment in Patients with Schizophrenia: A Randomized Clinical Trial. ***Iran J Psychiatry***. 2023;18(3):258-265.

74. Dartois LL, Stutzman DL, Morrow M. L-methylfolate Augmentation to Antidepressants for Adolescents with Treatment-Resistant Depression: A Case Series. ***J Child Adolesc Psychopharmacol***. 2019;29(5):386-391.

75. Roffman JL, Petruzzi LJ, Tanner AS, Brown HE, Erylmaz H, Ho NF, et al. Biochemical, physiological and clinical effects of l-methylfolate in schizophrenia: a randomized controlled trial. ***Mol Psychiatry***. 2018;23(2):316-322.

76. Siscoe KS, Lohr WD. L-Methylfolate supplementation in a child with autism and methyltetrahydrofolate reductase, enzyme gene C677TT allele. ***Psychiatr Genet***. 2017;27(3):116-119.

77. Sun C, Zou M, Zhao D, Xia W, Wu L. Efficacy of Folic Acid Supplementation in Autistic Children Participating in Structured Teaching: An Open-Label Trial. ***Nutrients***. 2016;8(6):337.

78. Bedson E, Bell D, Carr D, Carter B, Hughes D, Jorgensen A, et al. Folate Augmentation of Treatment--Evaluation for Depression (FolATED): randomised trial and economic evaluation. ***Health Technol Assess***. 2014;18(48):vii-viii,1-159.

79. Loria-Kohen V, Gómez-Candela C, Palma-Milla S, Amador-Sastre B, Hernanz A, Bermejo LM. A pilot study of folic acid supplementation for improving homocysteine levels, cognitive and depressive status in eating disorders. ***Nutr Hosp***. 2013;28(3):807-815.

80. Roffman JL, Lamberti JS, Achtyes E, Macklin EA, Galendez GC, Raeke LH, et al. Randomized multicenter investigation of folate plus vitamin B12 supplementation in schizophrenia. ***JAMA Psychiatry***. 2013;70(5):481-489.

81. Hill M, Shannahan K, Jasinski S, Macklin EA, Raeke L, Roffman JL, et al. Folate supplementation in schizophrenia: A possible role for MTHFR genotype. ***Schizophr Res***. 2011;127(1-3):41-45.

82. Christensen H, Aiken A, Batterham PJ, Walker J, MacKinnon AJ, Fenech M, et al. No clear potentiation of antidepressant medication effects by folic acid + vitamin B12 in a large community sample. ***J Affect Disord***. 2011;130(1-2):37-45.

83. Surman C, Ceranoglu A, Vaudreuil C, Albright B, Uchida M, Yule A, et al. Does L-Methylfolate Supplement Methylphenidate Pharmacotherapy in Attention-Deficit/Hyperactivity Disorder?: Evidence of Lack of Benefit From a Double-Blind, Placebo-Controlled, Randomized Clinical Trial. ***J Clin Psychopharmacol***. 2019;39(1):28-38.

84. Kuo SC, Yeh CB, Yeh YW, Tzeng NS. Schizophrenia-like psychotic episode precipitated by cobalamin deficiency. ***Gen Hosp Psychiatry***. 2009;31(6):586-588.

85. Pawełczyk T, Grancow-Grabka M, Żurner N, Pawełczyk A. Omega-3 fatty acids reduce cardiometabolic risk in first-episode schizophrenia patients treated with antipsychotics: Findings from the OFFER randomized controlled study. ***Schizophr Res***. 2021;230:61-68.

86. Szeszko PR, McNamara RK, Gallego JA, Malhotra AK, Govindarajulu U, Peters BD, et al. Longitudinal investigation of the relationship between omega-3 polyunsaturated fatty acids and neuropsychological functioning in recent-onset psychosis: A randomized clinical trial. ***Schizophr Res***. 2021;228:180-187.

87. Robinson DG, Gallego JA, John M, Hanna LA, Zhang JP, Birnbaum ML, et al. A potential role for adjunctive omega-3 polyunsaturated fatty acids for depression and anxiety symptoms in recent onset psychosis: Results from a 16 week randomized placebo-controlled trial for participants concurrently treated with risperidone. ***Schizophr Res***. 2019;204:295-303.

88. Qiao Y, Mei Y, Han H, Liu FJ, Yang XM, Shao Y, et al. Effects of Omega-3 in the treatment of violent schizophrenia patients. ***Schizophr Res***. 2018;195:283-285.

89. Kean JD, Sarris J, Scholey A, Silberstein R, Downey LA, Stough C. Reduced inattention and hyperactivity and improved cognition after marine oil extract (PCSO-524®) supplementation in children and adolescents with clinical and subclinical symptoms of attention-deficit hyperactivity disorder (ADHD): a randomised, double-b. ***Psychopharmacology (Berl)***. 2017;234(3):403-420.

90. Chhetry BT, Hezghia A, Miller JM, Lee S, Rubin-Falcone H, Cooper TB, et al. Omega-3 polyunsaturated fatty acid supplementation and white matter changes in major depression. ***J Psychiatr Res***. 2016;75:65-74.

91. Pawełczyk T, Grancow-Grabka M, Kotlicka-Antczak M, Trafalska E, Pawełczyk A. A randomized controlled study of the efficacy of six-month supplementation with concentrated fish oil rich in omega-3 polyunsaturated fatty acids in first episode schizophrenia. ***J Psychiatr Res***. 2016;73:34-44.

92. Bošković M, Vovk T, Koprivšek J, Plesničar BK, Grabnar I. Vitamin E and essential polyunsaturated fatty acids supplementation in schizophrenia patients treated with haloperidol. ***Nutr Neurosci***. 2016;19(4):156-161.

93. Pawełczyk T, Grancow M, Kotlicka-Antczak M, Trafalska E, Gȩbski P, Szemraj J, et al. Omega-3 fatty acids in first-episode schizophrenia - A randomized controlled study of efficacy and relapse prevention (OFFER): Rationale, design, and methods. ***BMC Psychiatry***. 2015;15:97.

94. Amminger GP, Mechelli A, Rice S, Kim SW, Kiler CM, McNamara RK, et al. Predictors of treatment response in young people at ultra-high risk for psychosis who received long-chain omega-3 fatty acids. ***Transl Psychiatry***. 2015;5(1):e495.

95. Smesny S, Milleit B, Hipler UC, Schäfer MR, Klier CM, Holub M, et al. Omega-3 fatty acid supplementation changes intracellular phospholipase A 2 activity and membrane fatty acid profiles in individuals at ultra-high risk for psychosis. ***Mol Psychiatry***. 2014;19(3):317-324.

96. Bentsen H, Osnes K, Refsum H, Solberg DK, Bøhmer T. A randomized placebo-controlled trial of an omega-3 fatty acid and vitamins E+C in schizophrenia. ***Transl Psychiatry***. 2013;3(12):e335.

97. Amminger GP, Chanen AM, Ohmann S, Kiler CM, Mossaheb N, Bechdolf A, et al. Omega-3 fatty acid supplementation in adolescents with borderline personality disorder and ultra-high risk criteria for psychosis: A post hoc subgroup analysis of a double-blind, randomized controlled trial. ***Can J Psychiatry***. 2013;58(7):402-408.

98. Meyer BJ, Grenyer BFS, Crowe T, Owen AJ, Grigonis-Deane EM, Howe PRC. Improvement of Major Depression is Associated with Increased Erythrocyte DHA. ***Lipids***. 2013;48(9):863-868.

99. Politi P, Cena H, Comelli M, Marrone G, Allegir C, Emanuele E, et al. Behavioral Effects of Omega-3 Fatty Acid Supplementation in Young Adults with Severe Autism: An Open Label Study. ***Arch Med Res***. 2008;39(7):682-685.

100. Germano M, Meleleo D, Montorfano G, Adorni L, Negroni M, Berra B, et al. Plasma, red blood cells phospholipids and clinical evaluation after long chain omega-3 supplementation in children with attention defict hyperactivity disorder (ADHD). ***Nutr Neurosci***. 2007;10(1-2):1-9.

101. Lai X, Zhang Q, Zhu J, Yang T, Guo M, Li Q, et al. A weekly vitamin A supplementary program alleviates social impairment in Chinese children with autism spectrum disorders and vitamin A deficiency. ***Eur J Clin Nutr***. 2021;75(7):1118-1125.

102. Guo M, Zhu J, Yang T, Lai X, Liu X, Liu J, et al. Vitamin A improves the symptoms of autism spectrum disorders and decreases 5-hydroxytryptamine (5-HT): A pilot study. ***Brain Res Bull***. 2018;137:35-40.

103. Liu J, Liu X, Xiong XQ, Yang T, Cui T, Hou NL, et al. Effect of vitamin A supplementation on gut microbiota in children with autism spectrum disorders - a pilot study. ***BMC Microbiol***. 2017;17(1):204.

104. Zhang J, Chen S, Chen J, Zhang H, Rao WW. Comparison of olanzapine-induced weight gain and metabolism abnormalities between topiramate and vitamin C in patients with schizophrenia: a preliminary study. ***Front Psychiatry***. 2023;14(May):1152953.

105. Wang Y, Liu XJ, Robitaille L, Eintracht S, MacNamara E, John Hoffer L. Effects of vitamin C and vitamin D administration on mood and distress in acutely hospitalized patients. ***Am J Clin Nutr***. 2013;98(3):705-711.

106. Raz R, Carasso RL, Yehuda S. The Influence of Short-Chain Essential Fatty Acids on Children with Attention-Deficit/Hyperactivity Disorder: A Double-Blind Placebo-Controlled Study. ***J Child Adolesc Psychopharmacol***. 2009;19(2):167-177.

107. Raison CL, Sanacora G, Woolley J, Heinzerling K, Dunlop BW, Brown RT, et al. Single-Dose Psilocybin Treatment for Major Depressive Disorder: A Randomized Clinical Trial. ***JAMA***. 2023;330(9):843-853.

108. Russell SE, Wrobel AL, Ashton MM, Turner A, Mohebbi M, Berk M, et al. Does Post-traumatic Stress Disorder Impact Treatment Outcomes within a Randomised Controlled Trial of Mitochondrial Agents for Bipolar Depression? ***Clin Psychopharmacol Neurosci***. 2023;21(3):457-465.

109. Weggen JB, Darling AM, Autler AS, Hogwood AC, Decker KP, Imthrun B, et al. Impact of acute antioxidant supplementation on vascular function and autonomic nervous system modulation in young adults with PTSD. ***Am J Physiol Regul Integr Comp Physiol***. 2021;321(1):R49-R61.

110. van der Burg KP, Cribb L, Firth J, Karmacoska D, Mischoulon D, Byrne GJ, et al. EPA and DHA as markers of nutraceutical treatment response in major depressive disorder. ***Eur J Nutr***. 2020;59(6):2439-2447.

111. Bot M, Brouwer IA, Roca M, Kohls E, Penninx BWJH, Watkins E, et al. Effect of Multinutrient Supplementation and Food-Related Behavioral Activation Therapy on Prevention of Major Depressive Disorder among Overweight or Obese Adults with Subsyndromal Depressive Symptoms: The MooDFOOD Randomized Clinical Trial. ***JAMA***. 2019;321(9):858-868.

112. Allott K, McGorry PD, Yuen HP, Firth J, Proffitt TM, Berger G, et al. The Vitamins in Psychosis Study: A Randomized, Double-Blind, Placebo-Controlled Trial of the Effects of Vitamins B12, B6, and Folic Acid on Symptoms and Neurocognition in First-Episode Psychosis. ***Biol Psychiatry***. 2019;86(1):35-44.

113. Adams JB, Audhya T, Geis E, Gehn E, Fimbres V, Pollard EL, et al. Comprehensive nutritional and dietary intervention for autism spectrum disorder—a randomized, controlled 12-month trial. ***Nutrients***. 2018;10(3):369.

114. Kałużna-Czaplińska J, Jóźwik-Pruska J, Chirumbolo S, Bjørklund G. Tryptophan status in autism spectrum disorder and the influence of supplementation on its level. ***Metab Brain Dis***. 2017;32(5):1585-1593.

115. Dean OM, Turner A, Malhi GS, Ng C, Cotton SM, Dodd S, et al. Design and rationale of a 16-week adjunctive randomized placebo-controlled trial of mitochondrial agents for the treatment of bipolar depression. ***Braz J Psychiatry***. 2015;37(1):3-12.

116. Adams JB, Audhya T, McDonough-Means S, Rubin RA, Quig D, Geis E, et al. Effect of a vitamin/mineral supplement on children and adults with autism. ***BMC Pediatr***. 2011;11:111.

117. Kałużna-Czaplińska J, Socha E, Rynkowski J. B vitamin supplementation reduces excretion of urinary dicarboxylic acids in autistic children. ***Nutr Res***. 2011;31(7):497-502.

118. Xia RR. Effectiveness of nutritional supplements for reducing symptoms in autism-spectrum disorder: A case report. ***J Altern Complement Med***. 2011;17(3):271-274.

119. Almeida OP, Marsh K, Alfonso H, Flicker L, Davis TME, Hankey GJ. B‐vitamins reduce the long‐term risk of depression after stroke: The VITATOPS‐DEP trial. ***Ann Neurol***. 2010;68(4):503-510.

120. Huss M, Völp A, Stauss-Grabo M. Supplementation of polyunsaturated fatty acids, magnesium and zinc in children seeking medical advice for attention-deficit/hyperactivity problems - an observational cohort study. ***Lipids Health Dis***. 2010;9:105.

121. Ford AH, Flicker L, McCaul K, van Bockxmeer F, Hegarty S, Hirani V, et al. The B-VITAGE trial: a randomized trial of homocysteine lowering treatment of depression in later life. ***Trials***. 2010;11:8.

122. Sivrioglu EY, Kirli S, Sipahioglu D, Gursoy B, Sarandöl E. The impact of ω-3 fatty acids, vitamins E and C supplementation on treatment outcome and side effects in schizophrenia patients treated with haloperidol: An open-label pilot study. ***Prog Neuropsychopharmacol Biol Psychiatry***. 2007;31(7):1493-1499.
