# Supplementary material for: Clinical outcomes of mitochondrial‐enhancing nutraceutical supplementation in psychiatric disorders: A systematic review
Source: Gen Psychiatr. 2026 Jun 1;39(3):e70023. doi: 10.1002/gps3.70023 (PMC13239229; doi:10.1002/gps3.70023)
Supplement: Supplementary file 2 — Supporting Information S2 [file GPS3-39-e70023-s002.docx]

**Supplementary table 2**. Studies reporting results of Vitamin D3 (Vit D3) supplementation in psychiatric conditions.

| **First author,**  **publication year**  **(PMID)** | **Patient/Control Characteristics** | | | **Phenotype**  **/Disease** | **Study group and**  **Nutraceutical supplements** | **Nutraceutical information** | | **Measures** | **Findings** | **Additional information** |
| --- | --- | --- | --- | --- | --- | --- | --- | --- | --- | --- |
|  | N  P/C | Age (y)  in P/C | Sex  M/F |  |  | Doses in IU/d* | Treatment time |  |  |  |
| Vyas,  2023  (37378490) | 0/720 | - | - | Older subjects at risk of depression | Intervention group 1:  Vit D3 + Omega-3  Intervention group 2:  Vit D3 + placebo  Intervention group 3:  placebo + Omega-3  Intervention group 4:  Placebo + placebo | Vit D3 (oral),  2000  Omega-3 (EPA/DHA),  0.465/0.375g/d | 2 y | PHQ-9 | Neither Vit D3 nor omega-3 fatty acids showed benefit for indicated and selective prevention of late-life depression | CTI: NCT01696435  This study uses data from the VITAL-DEP trial to evaluate preventive treatment for late-life depression |
| Rahman,  2023  (36462182) | 0/20487 | 69.3 | 11063/  9424 | Older adults | Intervention group:  Vit D3  Placebo group:  NA | Vit D3 (oral),  60000 IU/m | 5 y | PHQ-9 | Monthly supplementation with high-dose Vit D3 was not beneficial for measures of depression overall, but there was some evidence of benefit in subgroup analyses: Vit D3 supplementation improved PHQ-9 scores in those taking antidepressants at baseline (*p*=0.02), reduced the risk of antidepressant use in participants with a predicted 25(OH)D concentration <50 nmol/L and increased the risk in those with >50 nmol/L | CTI: ACTRN12613000743763.  This study uses the data from the D-Health study to assess depressive symptoms in its participants |
| Samadi,  2022  (36052304) | 75/0 | 8.6 (0.3) | 52/23 | ADHD | Intervention group:  Vit D3  Placebo group:  NA | Vit D3 (oral),  2000 | 12 w | Serum 25(OH)D, IL-6, TNF-α | Serum 25(OH)D levels increased significantly in the Vit D group (*p*=0.01). IL-6 and TNF-α levels did not show significant differences between the study groups (*p*=0.91 and *p*=0.76, respectively) | CTI: IRCT2016102324081N2 |
| Kumar,  2022  (35843459) | 59/0 | 37 (11) | 16/43 | MDD with severe  Vit D3 deficiency | Intervention group:  Vit D3  Placebo group:  starch | Vit D3 (oral),  60000 IU/5d | 12 w | HAM-D, MADRS, BDI, CGI-S, CGI-I, serum 25(OH)D | HAM-D scores decreased from 25.7 to 5.7 and from 25.8 to 5.0 in the Vit D3 and placebo groups, respectively, with no significant difference between study groups. No significant correlations were observed between 25(OH)D levels and depression rating scores | CTI: CTRI/2020/08/027428.  Participants were antidepressant-naive depressed adults who were also receiving escitalopram (10-20 mg/d) |
| Mohammadzadeh,  2022  (35685610) | 75/0 | 8.6 (0.3) | 52/23 | ADHD | Intervention group:  Vit D3  Placebo group:  NA | Vit D3 (oral),  2000 | 12 w | Serum 25(OH)D, PON-1 activity, TAC, 8-Isoprostane | A significant increase in 25(OH)D levels was observed in the Vit D3 group. Serum PON-1 and TAC concentrations decreased in both groups, with non-significant differences. 8-Isoprostane levels did not decrease | CTI: IRCT2016102324081N2 |
| Amini,  2022  (31900080) | 81/0 | 18-45  28 (1.3) | 0/81 | Postpartum depression | Intervention group 1:  Vit D3 + Ca  Intervention group 2:  Vit D3 + Placebo  Placebo group:  NA | Vit D3 (oral),  50000  Ca Carbonate, 500 mg/d | 8 w | EPDS, serum 25(OH)D, Ca, TNF-α, IL-6, oestradiol | The severity of depression was significantly reduced in the Vit D3 + Ca and Vit D3 + placebo groups than in the placebo alone group (-1.7 (3.4), -4.2 (6), and 0.25 (2.8), respectively; *p*=0.008). The effect of Vit D3 alone was larger than that of Ca (*p*=0.042 and *p*=0.004, respectively). No significant difference was observed for the other serum parameters | CTI: IRCT2016091416123N9 |
| Gaughran,  2021  (34962559) | 149/0 | 18-65  28.1 (8.5) | 89/60 | Early psychosis | Intervention group:  Vit D3  Placebo group:  Miglyol 812 oil | Vit D3 (oral),  120000 IU/m | 24 w | PANSS, GAF, CDS, WC, BMI, glycated HbA1c, T-c, CRP, Vit D3 | No differences were observed between the study groups on the PANSS, GAF, or CDS. Vit D3 supplementation did not improve mental or physical health outcomes in the study; however, patients showed a high prevalence of Vit D3 deficiency even in the early psychosis patients | CTI: ISRCTN12424842 |
| Okereke,  2020  (32749491) | 0/18353 | 67.4 (7) | 9330/  9023 | Adults aged 50 or older at risk of depression or recurrent depression | Intervention group:  Vit D3 + EPA/DHA  Placebo group:  NA | Vit D3 (oral),  2000  EPA/DHA, 0.465/0.375g/d | 5.3 y | PHQ-8, serum 25(OH)D | The risk of depression or clinically relevant depressive symptoms was not significantly different between the intervention and placebo groups; there were no significant between-group differences in the incidence or recurrence of depression or in changes in mood scores over time | CTI: NCT01169259.  CTI: NCT01696435.  Participants were free of clinically relevant depressive symptoms at baseline |
| Libuda,  2020  (32108263) | 113/0 | 11-18.9  16 (1.6) | 28/85 | Depressive symptoms | Intervention group:  Vit D3  Placebo group:  NA | VitD3 (oral),  2640 | 4 w | BDI-II, DISYPS-II, serum 25(OH)D | DISYPS parent ratings showed significant improvements in depressive symptoms in the intervention group (-1.23 to 0.13, *p*=0.016); however, BDI-II scores did not differ between groups (*p*=0.466) | CTI: DRKS00009758.  Effect measured in children and adolescents with depressive symptoms |
| Feng,  2020  (31914053) | 1/0 | 2.5 | 0/1 | ASD | Vit D3 | VitD3 (oral),  800 +  VitD3 (IM),  150000 IU/m | 24 w | Serum 25(OH)D, CARS | Although parents reported an improvement in the child's symptoms, the CARS score remained at baseline levels. Serum 25(OH)D increased significantly | Case report. Supplementation discontinued due to lack of efficacy |
| Gaughran,  2020  (31907006) | 192/0 | NA | NA | FEP | Intervention group:  Vit D3  Placebo group:  NA | Vit D3,  120000 IU/m | 24 w | PANSS, CDS, GAF, BMI, WC, CRP, cholesterol, HbA1c | Only the rationale for the study is reported | CTI: ISRCTN12424842 |
| Alghamdi,  2020  (31836995) | 62/0 | 41.5 (1.8) | NA | MDD | Intervention group:  Vit D3 + SOC  Control group:  None + SOC | Vit D3 (oral),  50000 IU/w | 12 w | BDI, serum Vit D3, serotonin | Women with moderate, severe, and extreme depression had significantly lower BDI scores after treatment. Women showed the greatest improvement in symptoms, and only men with severe depression showed a significant improvement in BDI scores | Serum serotonin levels were significantly increased after supplementation in both male and female patients |
| Infante,  2020  (30545280) | 1/0 | 23 | 1/0 | ASD | Intervention:  Vit D3 + EPA/DHA | Vit D3 (oral),  25000 IU/w  EPA/DHA, 2.4/1.2 g/d | 24 m | CGI-S, CGI-I, CARS, serum 25(OH)D, arachidonic acid, EPA, DHA | The patient showed remarkable improvement in symptoms of agitation, irritability, and self-injurious behavior | Case report. Risperidone dose was gradually reduced and discontinued after 18 months of follow-up |
| Kerley,  2020  (30301427) | 18/17 | 8 (3) (P)  9 (4) (C) | 26/9 | ASD | Intervention group:  Vit D3  Control group:  NA | Vit D3 (oral),  2000 | 20 w | Serum 25(OH)D levels | Children with ASD had a smaller increase in 25(OH)D levels (+26 vs +45 nmol/L) despite a significantly longer intervention period compared to C | CTI: NCT02508922 |
| Kaviani,  2020  (32217340) | 56/0 | 43 (1.2) | 6/50 | Depressive symptoms | Intervention group:  Vit D3  Placebo group:  Human oral paraffin | Vit D3 (oral),  50000 IU/2w  Placebo, NA | 8w | BDI-II, serum 25(OH)D, iPTH, oxytocin and platelet serotonin | Vit D3 supplementation significantly increased serum 25(OH)D levels (40.1 (29) nmol/L increase) and improved mood status (11.8 (6.4) decrease in BDI-II) in subjects with mild to moderate depression | CTI: NCT03766074 |
| Hansen,  2019  (30944021) | 62/0 | 16-65  39.2 (12) | 18/44 | Depressive symptoms | Intervention group:  Vit D3  Placebo group:  Lactose | Vit D3 (oral),  2800 | 12w | HAM-D, MDI, WHO-5, weight, WC, blood pressure, serum 25(OH)D, CRP, phosphate, ionized calcium, PTH | There was a decrease in depression scores from baseline to the end of the study in both groups, with no significant difference between groups. Patients with lower Vit D3 levels at baseline did not show significant differences in depression scores at the end of the study | CTI: NCT01390662 |
| Ghaderi,  2019  (30791895) | 60/0 | 44 (7) | 56/4 | SCZ | Intervention group:  Vit D3 + probiotics  Placebo group:  NA | Vit D3 (oral),  50000 IU/2w  Probiotic, 8x10^9^CFU/d | 12 w | PANSS, 25(OH)D, other metabolic parameters | Vit D3 plus probiotic supplementation had beneficial effects on PANSS score (-7.4 (9) vs -1.9 (7.5), *p*=0.01) and metabolic profiles (increased total antioxidant capacity, decreased malondialdehyde, fasting plasma glucose, insulin concentrations, and total cholesterol levels) | CTI: IRCT2017072333551N2 |
| Mazahery,  2019  (30607782) | 117/0 | 2.5-9  5.4 (1.4) | 100/17 | ASD | Intervention group 1:  Vit D3  Intervention group 2:  DHA  Intervention group 3:  VitD3 + DHA  Placebo group:  NA | Vit D3 (oral),  2000  DHA (oral),  0.722 g/d | 48 w | SRS-2, SPM, serum 25(OH)D, DHA | Treatment with Vit D3 alone did not result in significant improvement in core symptoms of ASD. On the other hand, DHA with or without Vit D3 may improve some core symptoms, but no significant conclusions can be drawn | CTI: ACTRN12615000144516 |
| Mousa,  2018  (28803880) | 0/48 | 32 (8.5) | 30/18 | Depressive symptoms in overweight or obese adults | Intervention group:  Vit D3  Placebo group:  NA | Vit D3 (oral), 100000 IU/once  + 4000 | 16 w | Serum 25(OH)D, anthropometric data, BDI | BDI scores were not different between the Vit D3 and placebo groups (-2 (4.5) vs -1.5 (2.9), *p*=0.7). There were also no differences in the BDI subscales. Serum 25(OH)D levels increased significantly in the intervention group compared to the placebo group (56 (20.8) vs. 2.7 (13.9) nmol/L, *p*<0.001) | CTI: NCT02112721.  The study was focused on obese adults with depressive symptoms but no clinically significant depression |
| Bahrami,  2018  (28759290) | 0/940 | 14.6 (1.5) | 0/940 | Adolescent girls | Intervention group:  Vit D3  Placebo group:  NA | Vit D3 (oral),  50000 IU/w | 9 w | BDI-II, Buss-Perry Aggression Questionnaire, serum 25(OH)D levels | Supplementation showed a beneficial reduction in mild, moderate, and severe depression scores (*p*=0.001), but no effect on aggression scores | CTI: IRCT201509047117N7.  The study was focused on depression and aggression scores in adolescent girls |
| Mohammadpour,  2018  (27924679) | 62/0 | 5-12  7.8 (1.6) | 46/16 | ADHD | Intervention group:  Vit D3 + methylphenidate  Placebo group:  Placebo (NA) + methylphenidate | Vit D3 (oral),  2000  Methylphenidate (Ritalin) | 8 w | CPRS, ADHD-RS, WPREMB, serum 25(OH)D | Evening symptoms and total score on the WPREMB scale were significantly different between the two groups at weeks 4 and 8 (*p*=0.013 and *p*=0.016, respectively), but no differences were found on the CPRS and ADHD-RS scales | CTI: IRCT201404222394N10 |
| Föcker,  2018  (29490621) | 200/0 | 11-18.9 | NA | ASD | Intervention group:  Vit D3  Placebo group:  NA | Vit D3 (oral),  2640 | 4 w | BDI-II, DISYPS-II, BIS-11, CBCL, YSR, IPAQ, FFQ, serum 25(OH)D, PTH, Ca, TSH, inflammatory markers, liver and renal parameters and alkaline phosphatase | Only the rationale for the study is reported | CTI: DRKS00009758 |
| Krivoy,  2017  (29226809) | 47/0 | 41 (10.5) | 32/15 | SCZ | Intervention group:  Vit D3 + Clozapine  Placebo group:  NA | Vit D3 (oral),  14000 IU/w | 8 w | PANSS, CDS, MoCA, serum 25(OH)D, metabolic parameters | There was no significant effect of Vit D3 on psychotic, depressive, or metabolic parameters; however, there was a trend toward improved cognition (effect size=0.17, significance lost after Bonferroni correction) | CTI: NCT01759485 |
| Kerley,  2017  (28626020) | 38/0 | 7.4 (13.6) | 33/5 | ASD | Intervention group:  Vit D3  Placebo group:  NA | Vit D3 (oral),  2000 | 20 w | ABC, DD-CGAS, SRS, serum 25(OH)D, immunity and systemic inflammatory markers | A significant improvement in self-care was observed on the DD-CGAS (*p*=0.02), but not on the primary endpoint of stereotypic behaviour. There was a greater improvement in total SRS and ABC scores in those with increased and decreased 25(OH)D, respectively. In contrast, there was also a trend toward less inappropriate speech in the placebo group (*p*=0.08) | CTI: NCT02508922 |
| Marsh,  2017  (28777983) | 33/0 | 18-70  44.3 | 17/16 | BD- depression and Vit D3 deficiency | Intervention group:  Vit D3  Placebo group:  NA | Vit D3 (oral),  5000 | 12 w | MADRS, HAM-A, YMRS | Vit D3 supplementation did not improve the reduction of mood or anxiety symptoms. After 12 weeks, Vit D3 levels increased to 28 ng/mL | CTI: NCT01884844 |
| Feng,  2017  (26783092) | 215/285 | 4.8 (1) (P)  5.1 (1.2) (C) | 173/42 (P)  225/60 (C) | ASD | Intervention group:  Vit D3  Control group:  none | Vit D3 (IM),  150000 IU/m  +  Vit D3 (oral),  400 | 12 w | ABC, CARS, and serum 25(OH)D | After supplementation, symptom scores on the CARS and ABC were significantly reduced. Regarding age, treatment effects were more pronounced in younger (≤3 years) children with ASD (*p*=0.038 for ABC, *p*=0.016 for CARS scores) | CTI: ChiCTR-CCC-13004498.  Only 37 of the total ASD children received Vit D3 supplementation |
| Vaziri,  2016  (27544544) | 0/153 | 26.3 (4.6) | 0/153 | Pregnant women | Intervention group:  Vit D3  Placebo group:  NA | Vit D3 (oral),  2000 | 10-12 w | EPDS and serum 25(OH)D | The intervention group had a greater reduction in depression scores than the control group at 38-40 weeks of gestation (*p*=0.01) and at 4 and 8 weeks postpartum (*p*<0.001). The intervention group had significantly higher 25(OH)D concentrations (*p*<0.001) | CTI: IRCT2015020310327N11 |
| Wang,  2016  (27022679) | 726/0 | 53.2 | 431/  295 | Dialysis patients with depression | Intervention group:  Vit D3  Placebo group:  NA | Vit D3 (oral),  50000 IU/w | 52 w | BDI-II, plasma Ca, phosphorous, iPTH, 25(OH)D, albumin and prealbumin | After stratification by depression type, the results support a significant association between supplementation and improvement in BDI-II scores in dialysis patients with vascular depression, with a nonsignificant effect on MDD | Dialysis patients with depression |
| Saad,  2016  (25876214) | 122/100 | 5.1 (1.4) (P)  4.9 (1.3) (C) | NA | ASD | Intervention group:  Vit D3  Control group:  NA | Vit D3 (oral),  300 IU/kg/d (max. 5000) | 12 w | Serum 25(OH)D levels, CARS | 25(OH)D levels are inversely correlated with autism rating scales, and high doses of vitD appear to improve ASD scores. Patients with 25(OH)D levels >40ng/ml showed a significant improvement in ASD rating scales (p≤0.05) | Only 83 subjects completed 3 months of treatment |
| Sikoglu,  2015  (26091195) | 35/0 | 12.2 (3.3) | 20/15 | BSD | Intervention group:  Vit D3  Placebo group:  NA | Vit D3 (oral),  2000 | 8 w | YMRS, CGI-S, CDRS, CSSR-S, MRI, serum Ca, phosphorous, PTH 25(OH)D, 1.25(OH)_2_D | After supplementation, BSD patients showed a significant decrease in YMRS (*p*=0.002) and CDRS scores (*p*=0.01); and a significant increase in ACC GABA (*p*=0.007) | This is an open-label study. BSD included both bipolar patients and patients with bipolar symptoms |
| Jia,  2015  (25511123) | 1/0 | 2.7 | 1/0 | ASD | Vit D3 | Vit D3 (IM),  150000 IU/m  +  Vit D3 (oral),  400 | 8 w | Serum 25(OH)D, ABC, CARS, CGI-S | Supplementation showed improvement in serum 25(OH)D levels (from 12.5ng/mL to 81.2ng/mL) and behavioural problems | Case report |
| Snoeijen-  Schouwenaars, 2015  (25497121) | 30/0 | 14.3 (4) | 17/13 | ID +  epilepsy | Intervention group 1:  Vit D3 200 UI/d  Intervention group 2:  Vit D 3 400 UI/d | Vit D3 (oral), 200 or 400 | 60 w | Serum 25(OH)D | The mean 25(OH)D concentration increased significantly from 57.40 (22.00) nmol/L at baseline to 89.47 (26.77) nmol/L at 15 months. By this time, 64% of the patients with a Vit D deficit at baseline had not achieved an adequate Vit D status | Supplementation doses varied from 200 IU/d to 400 IU/d depending on the Vit D3 status |
| Kilpinen-Loisa, 2009  (19845825) | 138/0 | 47.3 | 95/43 | ID | Intervention group 1:  Oral VitD3 + Ca  Intervention group 2:  IM VitD3 + Ca | Vit D3 (oral),  800  Vit D3 (IM)  150000 IU (single dose)  Ca, 1g | 24 w | Serum Ca, phosphate, alkaline phosphatase and 25(OH)D,  Plasma PTH | Serum 25(OH)D increased significantly at 6 months from 40 nmol/L to 82 nmol/L and from 41 nmol/L to 62 nmol/L in the oral and IM groups, respectively (*p*<0.001 for both). Plasma PTH decreased significantly in both groups (*p*<0.001). No supranormal Ca levels were observed in the IM group, while six participants in the oral group had mild to moderate hypercalcemia | The intervention was divided into two groups: oral and intramuscular, with no control group. The dosing in the study resulted in suboptimal correction of vitamin D status at 6 months |

C: control; CFU: colony forming units; CTI: Clinical Trial Identifier; d: day; F: female; g: gram; IM: intramuscular; IU: international units; m: month; M: male; N: number of subjects; NA: not available; P: patient; PMID: PubMed identifier; w: week; y: year.

Age is presented as mean (SD) or range.

* Doses are given in IU/d unless otherwise specified.

**Phenotype/Disease:**

ADHD: attention deficit hyperactivity disorder; ASD: autism spectrum disorder; BD: bipolar disorder; BSD: bipolar spectrum disorders; FEP: first-episode psychosis; ID: intellectual disability; MDD: major depressive disorder; SCZ: schizophrenia.

**Nutraceutical supplements:**

Ca: calcium; DHA: Docosahexaenoic Acid; EPA: Eicosapentaenoic Acid; SOC: standard of care; Vit: vitamin.

**Measures:**

1.25(OH)_2_D: 1-α-25-dihydroxyvitamin D3; 25(OH)D: 25-hydroxyvitamin D3; ABC: Aberrant Behaviour Checklist; ACC: Anterior cingulate cortex; ADHD-RS: Attention Deficit Hyperactivity Disorder Rating Scale-IV; BDI: Beck Depression Inventory; BDI-II: BDI Second edition; BIS-11: Barratt Impulsiveness Scale; BMI: Body Mass Index; Ca: calcium; CARS: Childhood Autism Rating Scale; CBCL: Child Behaviour Checklist; CDS: Calgary Depression Scale; CDRS: Children’s Depression Rating Scale; CGI: Clinical Global Impression; CGI-I: CGI for Improvement; CGI-S: CGI for severity of Illness; CPRS: Conner’s Parent Rating Scale; CRP: C-reactive protein; CSSR-S: Columbia-Suicide Severity rating Scale; DD-CGAS: Social Responsiveness Scale and rating on the Developmental Disabilities – Children’s Global Assessment Scale; DHA: docosahexaenoic acid; DISYPS-II: Diagnostic System for Mental disorders in childhood and Adolescence, Self- and Parent Rating; EPA: eicosapentaenoic acid; EPDS: Edinburgh Postnatal Depression Scale; FFQ: Food Frequency Questionnaire; GABA: Gamma-Aminobutyric Acid; GAF: Global Assessment of Functioning; HAM-A: Hamilton Anxiety Rating Scale; HAM-D: Hamilton Depression Rating Scale; HbA1c: Haemoglobin A1c or glycated haemoglobin; IL-6: interleukin 6; IPAQ: International Physical Activity Questionnaire; iPTH: intact-Parathyroid Hormone; MADRS: Montgomery-Asberg Depression Rating Scale; MDI: Major Depression Inventory; MoCA: Montreal Cognitive Assessment; MRI: Magnetic Resonance imaging; PANSS: Positive and Negative Syndrome Scale; PHQ-8: Patient Health Questionnaire-8; PHQ-9: Patient Health Questionnaire-9; PON-1: Paraxonase-1; PTH: Parathyroid Hormone; SPM: Sensory processing Measures; SRS: Social Responsiveness Scale; SRS-2: SRS Second edition; TAC: Total Antioxidant Capacity; T-c: total cholesterol; TNF-α: Tumour Necrosis Factor-Alpha; TSH: Thyroid-stimulating hormone; Vit D3: vitamin D3; WC: waist circumference; WHO-5: World Health Organization-Five Well-Being Index; WPREMB: Weekly Parent Ratings of Evening and Morning Behaviour; YMRS: Young Mania Rating Scale; YSR: Youth Self Report.

**Supplementary table 3**. Studies reporting results of supplementation of N-acetyl cysteine (NAC) in psychiatric conditions.

| **First author,**  **publication year**  **(PMID)** | **Patient/Control Characteristics** | | | **Phenotype**  **/Disease** | **Study group**  **Nutraceutical supplements** | **Nutraceutical information** | | **Measures** | **Findings** | **Additional information** |
| --- | --- | --- | --- | --- | --- | --- | --- | --- | --- | --- |
|  | N  P/C | Age (y)  in P/C | Sex  M/F |  |  | Doses in g/d | Treatment time |  |  |  |
| Kanaan,  2023  (37540942) | 104/0 | 20-72  42.98 | 44/60 | PTSD | Intervention group:  NAC  Placebo group:  NA | NAC, 2.7 | 12 w | CAPS-5, PCL-5, WHOQOL-BREF, HADS, HDRS, PHQ-15, AUDIT, substance craving questionnaire | After 12 w of treatment, CAPS-5 changes were not significant in either group (*p*=0.219). At the same time, PCL-5, HADS, HRDS, WHOQOL-BREF, AUDIT and craving item scores did not change significantly between groups (*p*=0.910) | Treatment-resistant PTSD |
| Neill,  2022  (35857752) | 75/0 | 39.74 | 61/14 | SCZ | Intervention group:  NAC  Placebo group:  NA | NAC, 2 | 52 w | PANSS, MCCB, MANSA, AQoL, CDS, SAFTEE | NAC did not significantly improve negative symptoms (*p*=0.62), overall cognition (*p*=0.71), or quality of life (*p*=0.11-0.57) at any time during the 1-year treatment period | CTI: ACTRN12615001273572. This study was designed to evaluate the efficacy of NAC on negative symptoms, quality of life, and cognition in treatment-resistant SCZ |
| Bortolasci,  2021  (34438354) | 60/0 | 46.3 (10.8) | 21/39 | BD | Intervention group:  NAC  Placebo group:  NA | NAC, 2 | 16 w | MADRS  Metabolite analyses, Glucagon, Amino acids | Participants with lower baseline amino acid levels responded better to NAC. A predictive model of MADRS improvement including 9 amino acids predicted 85% of the variance in MADRS score after treatment with NAC | CTI: ACTRN12612000830897 |
| Pesko,  2020  (32071590) | 4/0 | 14-17 | 4/0 | ASD | NAC | NAC, 1.2-2.4 | 4 w | ABC-I | There was a reduction in irritability symptoms and/or antipsychotic medication dosage in the four patients | The study was not a controlled trial |
| Ashton,  2020  (31661974) | 133/0 | >18 | NA | BD | Intervention group 1:  NAC  Intervention group2:  CT  Placebo group:  NA | NAC, 0.002  CT:  (NAC, 0.002,  ALCAR, 0.001,  CoQ, 0.2,  Vit E, 0.04032,  ALA, 0.15,  Mg, 0.064,  Vit B1, 0.1  Vit B2, 0.1  Vit B3, 0.2  Vit B5, 0.1  Vit B6, 0.1  Vit B9, 800 μg  Vit B12, 800 μg  Vit C, 0.242  Vit A, 900 μg  Vit D3, 12.5 μg  Vit H, 600 μg) | 16 w | MADRS, BDRS, HDRS, YMRS, LIFE-RIFT, SOFAS, Q-LES-Q-SF, CGI-BP, PGI | Participants with a more anti-inflammatory dietary inflammation index had less functional impairment (*p*=0.01). CT intervention may attenuate the adverse effects of a pro-inflammatory diet (*p*=0.03) on functioning. Participants with lower BMI who received CT (*p*=0.02) or NAC (*p*=0.02) showed greater clinician-rated improvement. Change in depression scores was not predicted by diet quality, dietary inflammatory index, or BMI scores in the CT intervention group compared to the placebo group. However, participants with better diet quality reported fewer symptoms of general and bipolar depression (*p*=0.01 and *p*=0.03, respectively) and greater clinician-rated improvement (*p*=0.02), regardless of treatment or time | CTI: ACTRN12612000830897 |
| Mullier,  2019  (31283822) | 20/74 | 25 (6) | 13/7 (P)  NA (C) | EP | Intervention group:  NAC  Control group:  NA | NAC, 2.7 | 6 m | fMRI | NAC supplementation increased functional connectivity along the cingulum, and more specifically between the caudal anterior part and the isthmus of the cingulate cortex. These functional changes can be partially explained by an increase in the centrality of these regions in the functional brain network | CTI: NCT01354132  The control group was of the same sex and age as the patient group |
| Yang,  2019  (30712814) | 19/0 | 49.1 (10.4) | 13/6 | SCZ | Intervention group:  NAC  Placebo group:  NA | NAC, 2.4 | 8 w | MMN, ASSR, negative symptoms | The study reported a NAC-associated increase in neural synchronization as assessed by ASSR in the SCZ. Total power at 40 Hz stimulation was improved with NAC, and intertrial coherence was improved at 20 Hz stimulation |  |
| Yang,  2018  (30176835) | 200/0 | 18-65 | NA | TRD | Intervention group:  NAC  Placebo group:  NA | NAC, 2 | 8 w  12 w | HDRS, BAI, IDS-SR, WHODAS-II, MoCA, fMRI, DTI,  blood biomarkers^Δ^, urine biomarkers^∇^ | Only the design and rationale of the study will be reported. Importantly, only patients with TRD and CRP levels between 0.85 and 10 mg/L will be included | CTI: NCT02972398  The study consisted of 12 w of treatment and 8 w of follow-up |
| Breier,  2018  (29588126) | 60/0 | 16-30  23.6 (4.7) | 47/13 | SCZ | Intervention group:  NAC  Placebo group:  NA | NAC, 0.6-3.6 | 52 w | PANSS, CGI-S, BACS, PSP, MRI | NAC significantly improved PANSS total (*p*<0.001), negative (*p*=0.024), and disorganized (*p*<0.001) symptom scores. NAC did not improve PANSS positive symptoms or BACS cognitive scores.  NAC did not significantly affect brain morphology compared to placebo during the study treatment period | Patients were in the early stages of schizophrenia |
| Conus,  2018  (29462456) | 63/0 | 18-40  25.4 (6.0) | 47/14 | EP | Intervention group:  NAC  Placebo group:  NA | NAC, 2.7 | 6 m | PANSS, GAF, SOFAS, UKU, MCCB (9 of 10 subtests), GSH_BC_, GSH_mPFC_, MRI, CYS_Pl_, GPx_BC_ | No changes in negative or positive symptoms or functional outcome were observed with NAC, but significant improvements in favour of NAC were found in neurocognition (processing speed). NAC also increased GSH_mPFC_ by 23% (*p*=0.005) and GSH_BC_ by 19% (*p*=0.05). In patients with high baseline GPx_BC_ compared to low baseline GPx_BC_, an association was observed between changes in positive symptoms and changes in redox status with NAC | Patients were included if they scored at the psychosis threshold subscale of the Comprehensive Assessment of At-Risk Mental States (CAARMS) scale and had been treated for psychosis for less than 12 m |
| Sepehrmanesh,  2018  (29126981) | 84/0 | 39.1 (2.1) | 38/41 | SCZ | Intervention group:  NAC  Placebo group:  NA | NAC, 1.2 | 12 w | PANSS, MMSE, neuropsychological test | NAC-treated patients showed significant improvement in positive (*p*=0.02) and negative symptoms. Also, the general and total PANSS scores of the NAC group decreased over time, while it increased for the placebo group. Regarding cognitive functions, improvement was observed in some of the investigated areas such as attention, short-term and working memory, executive function and processing speed |  |
| Rapado-Castro,  2017  (27894373) | 58/0 | 39.8 (12.3) | 34/24 | SCZ and BD | Intervention group:  NAC  Placebo group:  NA | NAC, 2g | 24 w | Attention, working memory and executive function | Participants treated with NAC had significantly better working memory performance at w 24 compared to placebo (*p*=0.027) | CTI: ACTRN12605000363684; 12605000362695 |
| Dean,  2017  (27316706) | 102/0 | 3.1-9.9 | 79/19 | ASD | Intervention group:  NAC  Placebo group:  NA | NAC, 0.5 | 6 m | SRS, CCC-2, RBS-R, DBC-P, PGI-I, CGI-I, CGI-S | There were no differences between the NAC and placebo- groups in any of the outcome measures for the primary or secondary endpoints. The number and severity of adverse events were not significantly different between groups | CTI: ACTRN12610000635066 |
| Wink,  2016  (27103982) | 31/0 | 4-12 | 24/7 | ASD | Intervention group:  NAC  Placebo group:  NA | NAC, 60mg/kg/d | 12 w | CGI-I, GSH | There was no statistically significant difference between the NAC and placebo groups on the CGI-I, but the glutathione (GSH) levels were significantly higher in the NAC group (*p*<0.05) | CTI: NCT00453180 |
| Monsivais,  2016  (27059873) | 1/0 | 38 | 1/0 | Unspecified psychotic disorder | NAC | NAC, 2.4  Fish oil, 2 | 1 y | BPRS, Neuroimaging, Neuropsychological testing | BPRS scores decreased from 59 at baseline to 37 at 1 y. Follow-up neuroimaging performed at 1 y showed stability of the white matter lesions. Neuropsychological testing also performed at 1 y showed statistically significant and reliable improvements in processing speed/attention and confrontation naming, as well as reductions in all measures of depression, anxiety, and PTSD symptoms | The study is a case report of a patient with G6PD (glucose-6-phosphate dehydrogenase) deficiency |
| Marler,  2014  (24815193) | 1/0 | 4 | M | ASD | NAC | NAC, 0.45-1.8 | 2 m | Self-injurious behaviour | The authors suggested an association between NAC supplementation and the patient’s cessation of severe self-injurious behaviour | The study is a case report |
| Ghanizadeh,  2013  (23886027) | 40/0 | 3.5-16 | - | ASD | Intervention group 1: NAC  Intervention group 2:  placebo | NAC, 1.2 | 8 w | ABC-I | After 8 w of treatment, risperidone plus NAC reduced irritability more than risperidone plus placebo, but no changes in core symptoms of ASD were observed | All patients were treated with risperidone |
| Hardan,  2012  (22342106) | 33/0 | 3.2-10.7 | 31/2 | ASD | Intervention group:  NAC  Placebo group:  NA | NAC, 0.9-2.7 | 12 w | ABC-I, CGI, SRS, RBS-R | Compared to placebo, NAC resulted in significant improvements on the ABC Irritability subscale (*p*<0.001; d=0.96) | CTI: NCT00627705 |
| Berk,  2008  (18436195) | 140/0 | 36.6 (10.9) | 98/42 | SCZ | Intervention group:  NAC  Placebo group:  NA | NAC, 2 | 4 w | PANSS, CGI, GAF, SOFAS, BAS, SAS, AIMS | NAC-treated subjects t improved more than placebo-treated subjects over the study  period in PANSS total (*p*=0.009), PANSS negative (*p*=0.018), PANSS general (*p*=0.035), CGI severity (*p*=0.004), and CGI improvement (*p*=0.025) scores. There was no significant change in the positive PANSS subscale. NAC treatment was also associated with an improvement in akathisia (*p*=0.022) | Patients were chronic |

C: control; CTI: Clinical Trial Identifier; d: day; F: female; g: gram; m: month; M: male; N: number of subjects; NA: not available; P: patient; PMID: PubMed identifier; w: week; y: year.

Age is presented as mean (SD) or range.

**Phenotype/Disease:**

ASD: autism spectrum disorder; BD: Bipolar disorder; EP: early psychosis; PTSD: posttraumatic stress disorder; SCZ: schizophrenia; TRD: treatment resistant depression.

**Nutraceutical supplements:**

ALA: alpha-lipoic acid; ALCAR: acetyl-L-carnitine; CoQ: coenzyme Q10; CT: combination treatment; Mg: magnesium; NAC: N-acetylcysteine; Vit: vitamin.

**Measures:**

ABC-I: Abusive Behaviour by Children-Indices; AIMS: Abnormal Involuntary Movements Scale; AQoL: Assessment of Quality of Life; ASSR: auditory steady-state response; AUDIT: Alcohol Use Disorders Identification Test; BACS: Brief Assessment of Cognition in Schizophrenia; BAI: Body Adiposity Index; BAS: Barnes Akathisia Scale; BDRS: Bipolar depression rating scale; BPRS: Brief Psychiatric Rating Scale; CAPS-5: Clinician-Administered PTSD Scale for DSM-5; CCC-2: Children’s Communication Checklist–Second Edition; CDS: Calgary Depression Scale; CGI: Clinical Global Impression; CGI-BP: CGI–Bipolar; CGI-I: CGI–Improvement scale; CGI-S: CGI–Severity scale; CYS_Pl_: plasmatic Cysteine; DBC-P: Developmental Behaviour Checklist–Primary Carer Version; DTI: Diffusion tensor imaging; fMRI: Functional MRI; GAF: Global Assessment of Functioning; GSH: glutathione; GSH_BC_: blood cells GSH; GSH_mPFC_: brain GSH; GPx_BC_: GSH peroxidase activity; HADS: Hospital Anxiety and Depression Rating Scale; HDRS: Hamilton Depression Rating Scale; IDS-SR: Inventory of Depressive Symptomatology Self Report; LIFE-RIFT: Range of Impaired Functioning Tool; MADRS: Montgomery-Asberg Depression Rating Scale; MANSA: Manchester Short assessment of quality of life; MCCB: MATRICS Consensus Cognitive Battery; MMN: mismatch negativity; MMSE: Mini-Mental State Examination; MoCA: Montreal Cognitive Assessment; MRI: Functional magnetic resonance imaging; PANSS: Positive and Negative syndrome Scale; PCL-5: PTSD Checklist for DSM-5; PGI: Parent Global Impression; PGI-I: PGI–Improvement scale; PHQ-15: Patient Health Questionnaire; PSP: Personal and Social Performance; Q-LES-Q-SF: Quality of Life Enjoyment and Satisfaction Questionnaire, Short Form; RBS-R: Repetitive Behaviour Scale–Revised; SAFTEE: Systematic Assessment for Treatment Emergent Events; SAS: Simpson-Angus Scale; SOFAS: Social and Occupational Functioning Assessment Scale; SRS: Social Responsiveness Scale; UKU: Udvalg for Kliniske Undersøgelser Side Effect Rating Scale; WHODAS-II: World Health Organization Disability Assessment Schedule-II; WHOQOL-BREF: World Health Organization Quality of Life Brief Version; YMRS: Young Mania Rating Scale.

^Δ^ Blood biomarkers measured in Yang et al 2018: CRP, NGAL, IL6, TNF-α, TNF-α-R2, IFN-γ, S100a (p11), NF-ĸB, S100b, insulin, leptin, vit D, BDNF, VEGF, EGF, cortisol, arginine-vasopressin, NPY, substance P, c-GMP, isoprostane, SOD, CAT, glutathione, NO, MDA2, calprotectin, endothelin, zonulin, aldosterone, thromboxane, cAMP.

∇ Urine biomarkers measured in Yang et al 2018: HVEM, LTB4, EGF, cortisol1, substance P1, midkine, c-GMP, isoprostane, calprotectin, aldosterone1; thromboxane1, leptin, m-hydroxyphenylacetate, formate, alanine, creatinine, malonate, N-methylnicotinamide.

**Supplementary table 4**. Studies reporting results of acetyl-L-carnitine (ALCAR) supplementation in psychiatric conditions.

| **First author,**  **publication year**  **(PMID)** | **Patient/Control Characteristics** | | | **Phenotype**  **/Disease** | **Study group**  **Nutraceutical supplements** | **Nutraceutical information** | | **Measures** | **Findings** | **Additional information** |
| --- | --- | --- | --- | --- | --- | --- | --- | --- | --- | --- |
|  | N  P/C | Age (y)  in P/C | Sex  M/F |  |  | Doses | Treatment time |  |  |  |
| Ziats,  2015  (25943046) | 1/0 | 4 | 1/0 | ASD | Intervention group: ALCAR | L-carnitine,  0.2 g/Kg/d (adjusted by bodyweight) | 4.5 m | Developmental milestones, Trimethyllysine, Gamma butyrobetaine, Free carnitine, Total acylcarnitines | Following carnitine supplementation, the patient’s regression ended, and the boy started gaining developmental milestones. At two months of treatment, plasma levels of carnitine were within the normal range | Case report; the patient carried a *TMLHE* (trimethyllysine hydroxylase, epsilon) gene mutation (c.961_962del; p.I321fs; ChrX: 154736591) predicted to be deleterious according to ACMG guidelines. At baseline, the patient had a low C0 component of acylcarnitine, but all other components of the acylcarnitine profile were within the normal range |

C: control; d: day; F: female; g: gram; m: month; M: male; N: number of subjects; P: patient; PMID: PubMed identifier; y: year.

ACMG: American College of Medical Genetics and Genomics.

**Phenotype/Disease:**

ASD: autism spectrum disorder.

**Nutraceutical supplements:**

ALCAR: acetyl-L-carnitine.

**Supplementary table 5**. Studies reporting results of Coenzyme Q10 (CoQ10) supplementation in psychiatric conditions.

| **First author,**  **publication year**  **(PMID)** | **Patient/Control Characteristics** | | | **Phenotype**  **/Disease** | **Study group**  **Nutraceutical supplements** | **Nutraceutical information** | | **Measures** | **Findings** | **Additional information** |
| --- | --- | --- | --- | --- | --- | --- | --- | --- | --- | --- |
|  | N  P/C | Age (y)  in P/C | Sex  M/F |  |  | Doses (g/d) | Treatment time |  |  |  |
| Maguire,  2021 (33347024) | 72/0 | 18-70 | 51/21 | SCZ, SCZA | Intervention group: CoQ10  Placebo group:  NA | CoQ10_,_ 0.3 | 3 m  6 m | CPT-IP, SWM, WMS, CoQ10, Lactate, Processing speed, Executive function, General cognitive function, Energy, Depression, Anxiety, Negative symptoms, Quality of life, Functional status, Physical activity, Blood pressure | Overall, there was no effect of CoQ10 supplementation on the primary outcome (CPT-IP, SWM, WMS). In addition, except for plasma levels, CoQ10 supplementation had no effect on the secondary outcomes | At 3 m, CoQ10 levels were significantly higher in the intervention group; however, this difference was not present at 6 m |
| Jahangard,  2019 (31346916) | 89/0 | 38.5 (10.8) | 11/58 | BD | Intervention group: CoQ10  Placebo group:  NA | CoQ10, 0.2 | 8 w | MADRS, TAC, TTG, CAT, NO, MDA, TNF-α, IL-6, IL-10 | The pattern of results suggests that compared to placebo and over a time course of 8 w, adjuvant CoQ10 had a beneficial effect on oxidative stress and inflammatory biomarkers in patients with BD during the depressive episode. MARDS scores were significantly lower in the CoQ10 group at w 8 of the study (*p*=0.01) | CTI: RCT2016103022965N5.  The participants were in a depressive phase. Serum levels of TTG, TAC, TNF-α and IL-10 increased significantly. No statistical changes were observed for CAT, MDA and IL-6 |
| Mousavinejad, 2018 (29684771) | 90/90 | 3-12  7.9 (2.6) (P)  7.6 (2.6) (C) | 66/24 (P)  66/24 (C) | ASD | Intervention group: CoQ10  Placebo group:  Starch  Control group | CoQ10, 0.03  CoQ10, 0.06  Starch, NA | 100 d | CARS, CoQ10, MDA, TAS, GR, GSSG, GPx, SOD, Gastrointestinal problems, Sleep disorders, Verbal communication, Playing with friend | Serum MDA (r^2^=0.668) and TAS (r^2^=0.007), as well as the activity of the antioxidant enzymes SOD (r^2^=0.01) and GPx (r^2^=0.001), correlated with the CARS score. Based on the results, high doses of CoQ10 can improve gastrointestinal problems (*p*=0.004) and sleep disturbances (*p*=0.005) in children with ASD by increasing serum CoQ10 | Oxidative stress factors were directly related to the severity of autism. CoQ10 and oxidative stress could be used as relevant biomarkers to help improve ASD |

C: control; CTI: Clinical Trial Identifier; d: day; F: female; g: gram; m: months; M: male; N: number of subjects; NA: not available; P: patient; PMID: PubMed identifier; w: week; y: year.

Age is presented as mean (SD) or range.

**Phenotype/Disease:**

ASD: autism spectrum disorders; BD: bipolar disorder; SCZ: schizophrenia; SCZA: schizoaffective disorder.

**Nutraceutical supplements:**

CoQ10: coenzyme Q10.

**Measures:**

CAT: catalase activity; CARS: Childhood Autism Rating Scale; CPT-IP: continuous performance task – identical pairs; GPx: glutathione peroxidase; GR: glutathione reductase; GSSG: glutathione disulfide; IL-10: interleukin-10; IL-6: interleukin-6; MADRS: Montgomery–Asberg Depression Rating Scale; MDA: malondialdehyde; NO: nitric oxide; SOD: superoxide dismutase; SWM: spatial working memory; TAC: total antioxidant capacity; TAS: total antioxidant status; TNF-α: tumour necrosis factor-alpha; TTG: total thiol groups WMS: Wechsler Memory Scale.

**Supplementary table 6**. Studies reporting results of alpha-lipoic acid (ALA) supplementation in psychiatric conditions.

| **First author,**  **publication year**  **(PMID)** | **Patient/Control Characteristics** | | | **Phenotype**  **/Disease** | **Study group**  **Nutraceutical supplements** | **Nutraceutical information** | | **Measures** | **Findings** | **Additional information** |
| --- | --- | --- | --- | --- | --- | --- | --- | --- | --- | --- |
|  | N  P/C | Age (y)  in P/C | Sex  M/F |  |  | Doses (g/d) | Treatment time |  |  |  |
| De Lima,  2023  (36584248) | 35/0 | 37.8 (8.0) | 23/12 | SCZ | Intervention group:  ALA  Placebo group:  NA | ALA, 0.1 | 16 w | Positive symptoms, cognitive function, extrapyramidal symptoms, BMI, oxidative/inflammatory parameters | No significant improvement in BMI, cognition, psychopathology, antipsychotic side effects, or oxidative stress and inflammation was observed in the experimental group compared to the placebo group in | CTI: NCT03788759.  There was a significant decrease in red blood cells, white blood cells, and platelets in the ALA-treated group that requires further investigation |
| Mishra,  2022 (36069950) | 20/0 | 18-65  36.0 (10.4) | 12/8 | TR-SCZ | Intervention group:  ALA  Placebo group:  NA | ALA, 0.3 | 8 w | SAPS, SANS, SCoRS, UKU  BDNF, MDA and GSH | ALA supplementation improved psychopathology and reduced oxidative stress in patients. Significant improvement was seen in the SANS score in the intervention group and in BDNF levels in the placebo group | CTI: CTRI/2020/03/023707.  There was no significant improvement in SAPS score |
| Sanders,  2017  (29053478) | 10/0 | 18-60  38.5 (7.3) | 6/4 | SCZ | Intervention group:  ALA | ALA, 0.1 | 4 m | BPRS, TMT, Block Corsi Test, Subtest Digit Span, Category (Animal) Fluency, COWAT-FAS, AVLT, BMI, ALT, AST, HbA1c, folic acid, Vit B12, hs-CRP, GSH, nitrite, TBARS, IL-1β, IL-4, Interferon γ, IDO | The intervention showed a 63.9% reduction in BPRS score. Robust improvement in measures of psychopathology,  neurocognitive parameters, extrapyramidal symptoms, and reduced lipid peroxidation.  No significant differences were found in anthropometric measures and biochemical parameters, except for TBARS and folic acid |  |
| Vidović,  2017  (28009525) | 18/0 | 25-60  39.7 (8.4) | 8/10 | SCZ | Intervention group:  ALA | ALA, 0.5 | 3 m | Glucose, HDL-c, LDL-c, T-c, TG, AST, ALT, GGT, BMI, WC, body fat, FLI, adiponectin; leptin, O2-, MDA, TAC, SFA, MUFA, PUFA, n-6 PUFA, n-3 PUFA, n6/n3 | After the intervention, a significant increase in the plasma adiponectin concentrations and a decrease in fasting glucose and AST were observed |  |
| Vidović,  2014  (25191766) | 18/38 | 39.7 (8.4) (P)  41.1 (10.6) (C) | 8/10 (P)  26/12 (C) | SCZ  C | Intervention group:  ALA  Control group | ALA 0.5 | 45 d  90 d | BMI, Fat %, WC, WHR, SBP, DBP, glucose, T-c, HDL-c, LDL-c, TG, uric acid, TBARS, AOPP, TAS, -SH, SOD | ALA supplementation significantly reduced TBARS, AOPP and improved TAS levels in C, while there were no significant differences in P |  |
| Emsley,  2014  (24996507) | 33/0 | 18-48 | NA | FE-SCZ,  FE-SCZA,  FE-SD | Intervention group:  PUFA, ALA  Placebo group:  Olive oil | PUFA, 3  ALA, 0.3  Olive oil, NA | 2 y  or until relapse | Mean times to relapse | No differences in mean time to relapse: 39.8 ± 25.4 and 38.3 ± 26.6 weeks for the ω-3 PUFAs + ALA and placebo groups, respectively (*p*=0.9) | CTI: DOH-27-0910-3386 |

C: control; CTI: clinical trial identifier; d: day; F: female; g: gram; m: month; M: male; N: number of subjects; NA: not available; P: patient; PMID: PubMed identifier; w: week; y: year.

Age is presented as mean (SD) or range.

**Phenotype/Disease:**

FE-SCZ: first-episode schizophrenia; FE-SCZA: FE-schizoaffective disorder; FE-SD: FE-schizophreniform disorder; TR-SCZ: treatment-resistant SCZ.

**Nutraceutical supplements:**

ALA: alpha-lipoic acid; PUFA: omega-3 polyunsaturated fatty acids.

**Measures:**

ALT: alanine aminotransferase; AOPP: Advanced oxidation protein products; AST: aspartate aminotransferase activity; AVLT: Rey Auditory Verbal Learning Test; BDNF: Brain-Derived Neurotrophic Factor; BMI: body mass index; BPRS: Brief Psychiatric Rating Scale; COWAT-FAS: Controlled Oral Word Association Test using F-A-S letters; DBP: diastolic blood pressure; FLI: fatty liver index; GGT: gamma-glutamyl transferase; GSH: reduced glutathione; HbA1c: glycohemoglobin; HDL-c: high-density lipoprotein cholesterol; hs-CRP: high-sensitive C-reactive protein; IDO: indoleamine 2,3-dioxygenase activity; IL-1β: interleukin 1β; IL-4: interleukin 4; LDL-c: low-density lipoprotein cholesterol; MDA: malondialdehyde; MUFA: monounsaturated fatty acid; O2-: oxygen; PUFA: polyunsaturated fatty acid; SANS: Scale for the Assessment of Negative Symptoms; SAPS: Scale for the Assessment of Positive Symptoms; SBP: systolic blood pressure; SCoRS: Schizophrenia Cognitive Rating Scale; SFA: saturated fatty acids; -SH: sulfhydryl groups; SOD: superoxide dismutase; TAC: total antioxidant capacity; TAS: total antioxidant status; TBARS: thiobarbituric acid-reactive substances; T-c: total cholesterol; TG: triglycerides; TMT: Trail Making Test; UKU: Udvalg for Kliniske Undersøgelser Side Effect Rating Scale; Vit: Vitamin; WC: waist circumference; WHR: waist to hip ratio.

**Supplementary table 7**. Studies reporting results of Magnesium (Mg) supplementation in psychiatric conditions.

| **First author,**  **publication year**  **(PMID)** | **Patient/Control Characteristics** | | | **Phenotype**  **/Disease** | **Study group**  **Nutraceutical supplements** | **Nutraceutical information** | | **Measures** | **Findings** | **Additional information** |
| --- | --- | --- | --- | --- | --- | --- | --- | --- | --- | --- |
|  | N  P/C | Age (y)  in P/C | Sex  M/F |  |  | Doses (g/d)* | Treatment time |  |  |  |
| Hemamy,  2021  (33865361) | 66/0 | 6-12  9.1 (1.6) | 46/20 | ADHD | Intervention group:  Vit D, Mg  Placebo group:  Paraffin oil, Microcrystalline cellulose, Stearic acid | Mg, 6 mg/Kg/d  Vit D, 50000IU/w  Paraffin oil, NA  Microcrystalline cellulose, NA  Stearic acid, NA | 8 w | SDQ,  25(OH)D, Mg | After 8 weeks, serum levels of 25(OH)D and Mg increased significantly in the intervention group compared to the placebo group. In addition, significant reductions were observed in emotional problems (*p*=0.001), conduct problems (*p*=0.002), peer problems (*p*=0.001), prosocial score (*p*=0.007), total difficulties (*p*=0.001), externalizing score (*p*=0.001), and internalizing score (*p*=0.001) | CTI: IRCT2016030326886N1.  Results were obtained in ADHD children with serum 25(OH)D levels of less than 30 ng/dL and serum Mg levels less than 2.3 mg/dL |
| Afsharfar,  2021  (33745609) | 46/21 | 54.5 (7.3) (P)  52.8 (8) (C) | NA | MDD | Intervention group:  Mg  Control group:  Starch powder | Mg, 0.5  Starch powder, NA | 8 w | BDI-II, BDNF, Mg | At the end of the intervention, Mg supplementation had a significant effect on Beck's test (*p*=0.01) and serum Mg (*p*=0.001), but had no significant effect on BDNF levels (*p*=0.507) between the two groups | CTI: IRCT20190317043084N1 |
| Debi Ann,  2020  (33036783) | 2/0 | 4 | 1/1 | ASD | Intervention group:  Vit B6, Mg | Vit B6, 0.03  Mg, 0.06 | 2 m | CARS2-ST, ATEC, BEARS, 6-GSI | The two patients showed hyperactive behaviour at the end of the treatment, while there were no changes in ATEC, BEARS, and 6-GSI scores | CTI: CTRI/2019/07/020102.  This study is a two case reports from a clinical trial |
| Tarleton, 2017  (28654669) | 126/0 | 52.7/0 | 44/68 | MDD | Intervention group: Mg | Mg, 0.248 | 6 w | PHQ-9, GAD-7 | After 6 w, there was a clinically significant improvement in depression scores by an average of 6 points and in anxiety by over 4 points | The intervention consisted of 6 w of active treatment and 6 w of control (no treatment) |
| Rajizadeh,  2017  (28241991) | 60/0 | 20-60  32.1 (8.6) | 14/39 | MDD with Mg deficiency | Intervention group:  Mg  Placebo group:  Starch powder | Mg, 0.5  Starch powder, NA | 8 w | BDI-II | After supplementation, the reduction in BDI score was more significant in the intervention group than in the placebo group (*p*=0.02), with mean changes of 15.7 (8.9) and 10.4 (7.9), respectively | The criterion for the diagnosis of depression was a score >11 on the BDI-II. The criterion for Mg deficiency was a serum Mg level <1.8 mg/dL in men and <1.9 mg/dL in women |

C: control; CTI: Clinical Trial Identifier; d: day; F: female; g: gram; IU: international units; m: months; M: male; N: number of subjects; NA: not available; P: patient; PMID: PubMed identifier; w: week; y: year.

Age is presented as mean (SD) or range.

**Phenotype/Disease:**

ADHD: Attention deficit hyperactivity disorder; ASD: Autism Spectrum Disorder; MDD: Major Depressive Disorder.

**Nutraceutical supplements:**

Mg: magnesium; Vit D: vitamin D; Vit B6: vitamin B6 (pyridoxine hydrochloride).

**Measures:**

25(OH)D: 25-hydroxyvitamin D; 6-GSI: 6-item Gastrointestinal Severity Index; ATEC: Autism Treatment Evaluation Checklist; BDNF: brain-derived neurotrophic factor; BDI-II: Beck Depression Inventory-II; BEARS: BEARS sleep screening tool; CARS2-ST: Childhood Autism Rating Scale, Second Edition – Standard Version; GAD-7: Generalized Anxiety Disorders-7; Mg: magnesium; PHQ-9: Patient Health Questionnaire-9; SDQ: Strengths and Difficulties Questionnaire.

**Supplementary table 8**. Studies reporting results of Vitamin B6 (Vit B6) supplementation in psychiatric conditions.

| **First author,**  **publication year**  **(PMID)** | **Patient/Control Characteristics** | | | **Phenotype**  **/Disease** | **Study group**  **Nutraceutical supplements** | **Nutraceutical information** | | **Measures** | **Findings** | **Additional information** |
| --- | --- | --- | --- | --- | --- | --- | --- | --- | --- | --- |
|  | N  P/C | Age (y)  in P/C | Sex  M/F |  |  | Doses (g/d)* | Treatment time |  |  |  |
| Rizzo,  2022  (35215501) | 34/0 | 4-17  10.4 (3.5) | 30/4 | CTD or TS with anxiety symptoms | Intervention Group 1:  L-Theanine, Vit B6  Intervention Group 2:  psychoeducation | L-Theanine, 0.2  Vit B6, 0.0028 | 2 m | YGTSS, MASC | The mean total decrease in YGTSS at 2 m was 8.85 (43.5%) in Intervention Group 1 versus 3.4 (18.3%) in Intervention Group 2 (*p*=0.046). No statistical differences were observed between the two study groups in the severity of anxiety symptoms as assessed by the MASC after 2 m of treatment.  Supplementation with L-theanine and Vit B6 was significantly more effective than psychoeducation in reducing tics and co-occurring disorders |  |
| Badrfam,  2021  (34662000) | 50/0 | 18-65 | 13/37 | BD type 1 | Intervention group:  Vit B6  Placebo group:  NA | Vit B6, 0.08 | 8 w | Homocysteine, Inflammatory markers, Fat profile, Lab tests,  YMQ, MMSE, PSQI, Appetite questionnaire, Anthropometric measures | A significant improvement in cognitive status was observed in the placebo group compared to the Vit B6 group (25.2 (2.0) vs. 24.4 (3.3), respectively, *p*=0.01) according to the MMSE. No significant differences were observed between the study groups on the YMQ and the PSQI. There were no significant differences between groups in the laboratory tests | The addition of Vit B6 to lithium was not associated with a significant improvement in mood status |
| Debi Ann,  2020  (33036783) | 2/0 | 4 | 1/1 | ASD | Vit B6  Mg | Vit B6, 0.03  Mg, 0.06 | 2 m | CARS2, ATEC, BEARS,  6-GSI, NADRPS | The two patients showed hyperactive behaviour at the end of the treatment, while there were no changes in ATEC, BEARS, and 6-GSI scores | CTI: CTRI/2019/07/020102.  This study is report of two cases from a clinical trial |
| Itokawa,  2018  (29064136) | 10/0 | 38-64 | 3/7 | SCZ, SCZA | Vit B6 | Vit B6, 1.2, 1.8 or 2.4 | 24 w | Pentosidine, PANSS | Decreased plasma pentosidine levels were observed in eight patients.  Two patients showed marked improvement in their psychological symptoms | CTI: UMIN000006398  Patients had elevated plasma pentosidine levels. The daily dose (1.2, 1.8 or 2.4 g/d) was determined by the treating physician |
| Kałużna-  Czaplińska,  2011  (21530806) | 30/21 | 4-11 | 27/3 (P)  20/1 (C) | ASD | Intervention group 1:  Folic acid, Vit B6,  Vit B12  Intervention group 2:  Vit B6, Vit B12  Control group | Folic acid, 400 μg  Vit B6, 0.2  Vit B12, 1.2 μg | 3 m | Urinary homocysteine | Urinary homocysteine levels were significantly higher in ASD children before the intervention (2.41 (1.10)) compared to both vitamin supplementation (1.13 (0.44)) and non-ASD control children (1.33 (0.39)). The intake of Vit B6 and Vit B12 together with folic acid was found to be more effective in reducing urinary homocysteine levels than the intake of vitamins B6 and B12 alone |  |

C: control; CTI: Clinical Trial Identifier; d: day; F: female; g: gram; m: month; M: male; N: number of subjects; NA: not available; P: patient; PMID: PubMed identifier; w: week; y: year.

* Doses are given in g/d unless otherwise specified.

**Phenotype/Disease:**

ASD: autism spectrum disorders; BD: bipolar disorder; CTD: chronic tic disorder; SCZ: schizophrenia; SCZA: schizoaffective disorder; TS: Tourette syndrome.

**Nutraceutical supplements:**

L-Theanine; Mg: magnesium; Vit B6: vitamin B6; Vit B12: vitamin B12.

**Measures:**

6-GSI: 6 items gastrointestinal severity index; ATEC: Autism Treatment Evaluation Checklist; BEARS: BEARS sleep screening tool; CARS2: Childhood Autism Rating Scale, Second Edition; MASC: Multidimensional Anxiety Scale for Children; MMSE: Mini-Mental State Examination; NADRPS: Naranjo Adverse Drug Reaction Probability Scale; PANSS: positive and negative syndrome scale; PSQI: Pittsburgh Sleep Quality Index; YGTSS: Yale Global Tic Severity Scale; YMQ; Young Mania Questionnaire.

**Supplementary table 9**. Studies reporting results of Vitamin B7 (Vit B7) supplementation in psychiatric conditions.

| **First author,**  **publication year**  **(PMID)** | **Patient/Control Characteristics** | | | **Phenotype**  **/Disease** | **Study group and**  **Nutraceutical supplements** | **Nutraceutical information** | | **Measures** | **Findings** | **Additional information** |
| --- | --- | --- | --- | --- | --- | --- | --- | --- | --- | --- |
|  | N  P/C | Age (y)  in P/C | Sex  M/F |  |  | Doses (g/d) | Treatment time |  |  |  |
| Reininghaus,  2020  (33171595) | 82/0 | 20-55  41.6 (12.9) | 18/64 | MDD | Intervention group:  Probiotics^§^  Vit B7, Common horsetail, Fish collagen, Keratin plus matrix  Placebo group:  Vit B7, Common horsetail, Fish collagen, Keratin plus matrix | Probiotics, ≥7.5 billion organisms  Vit B7, 0.125  Horsetail, 0.03  Collagen, 0.03  Keratin, 0.03 | 28 d | HDRS, BDI-II, SCL-90, GSI, PSDI, PST, MSS, GLQI, BMI, Zonulin, Microbiome analysis (16S), WHR | Vit B7 was not the target of the study | CTI: NCT03300440  Probiotic intervention did not differ from placebo in clinical outcome measures. *Ruminococcus gauvreauii* and *Coprococcus 3* were more abundant, and β-diversity was higher in the probiotic group after 28 d of treatment. KEGG analysis showed increased inflammation-regulating and metabolic pathways in the intervention group |

C: control; CTI: Clinical Trial Identifier; d: day; F: female; g: gram; M: male; N: number of subjects; P: patient; PMID: PubMed identifier; y: year.

Age is presented as mean (SD) or range.

**Phenotype/Disease:**

MDD: major depressive disorder.

**Nutraceutical supplements:**

Vit B7: vitamin B7.

**Measures:**

BDI-II: Beck Depression Inventory-II; BMI: Body Mass Index; GLQI: gastrointestinal quality of life; GSI: Global Symptom Index; HDRS: Hamilton Depression Rating Scale; KEGG: Kyoto Encyclopaedia of Genes and Genomes; MSS: Mania Self Rating Scale; PSDI: Positive Symptom Distress Index; PST: Positive Symptom Total; SCL-90: Symptom Checklist-90-Revised; WHR: Waist to hip ratio.

^§^ *B. bifidum* W23, *B. lactis* W51, *B. lactis* W52, *L. acidophilus* W22, *L. casei* W56, *L. paracasei* W20, *L. plantarum* W62, *L. salivarius* W24, *L. lactis* W19.

**Supplementary table 10**. Studies reporting results of folic acid (FA) supplementation in psychiatric conditions.

| **First author,**  **publication year**  **(PMID)** | **Patient/Control Characteristics** | | | **Phenotype**  **/Disease** | **Study group**  **Nutraceutical supplements** | **Nutraceutical information** | | **Measures** | **Findings** | **Additional information** |
| --- | --- | --- | --- | --- | --- | --- | --- | --- | --- | --- |
|  | N  P/C | Age (y)  in P/C | Sex  M/F |  |  | Doses (g/d)* | Treatment time |  |  |  |
| Hosseini, 2023 (37575608) | 50/0 | 32.8 (9.2) | 34/16 | SCZ | Intervention group:  Memantine  Placebo group:  FA | Memantine, 0.005 - 0.020  FA, 0.001 | 12 w | WAIS-III | FA was not the target of the study. It was used as a placebo.  After 12 w, the mean WAIS-III score was higher in the memantine group (100.4 (27.7)) compared to placebo (84.7 (20)). | CTI: IRCT20190606043827N1.  The aim of the study was to compare the effect of memantine versus FA on cognitive impairment in SCZ |
| Dartois,  2019  (31058543) | 10/0 | 14.4 (2.8) | 2/8 | Treatment- resistant depression | Intervention group:  FA (L-methylfolate) | FA, between 0.002 and 0.015 | 16-73 w | No objective reports | Efficacy was assessed by subjective patient, family, and provider reports. In general, FA supplementation was found to be modestly effective, with 8 out of 10 patients reporting a subjective improvement in symptoms of depression, anxiety, and irritability | Approximately 80% of patients were heterozygotes for the two *MTHFR* gene variants studied (50% 1298A>C; 30% 677C>T and 20% both), indicating reduced MTHFR activity |
| Surman,  2019  (30566416) | 41/0 | 39.5 (10.1) | 15/26 | ADHD | Intervention group:  FA (L-methylfolate)  Placebo group:  NA | FA, 0.015 | 12 w | CGI, AISRS, GAF, HAM-A, HAM-D, CANTAB, BRIEF-A, ASR, SAS-SR, CBS-SR | FA had no significant effect over placebo except for improvement from abnormal measures on the mean adaptive dimension of the ASR scale (*p*=0.04). Methylphenidate dosage was significantly higher over time in individuals with FA (*p*=0.007) |  |
| Roffman,  2018  (28289280) | 55/0 | 45.5 (11.1) | 43/12 | SCZ | Intervention group:  FA (L-methylfolate)  Placebo group:  NA | FA, 0.015 | 12 w | FA, PANSS-T, PANSS-N, PANSS-G, SANS, CDSS, MCCB, MRI | Compared to placebo, FA increased plasma FA levels (*p*<0.001, d=1.00) and improved PANSS-T (*p*=0.03, d=0.61) as well as PANSS-N and PANSS-G subscales. Changes in PANSS-T and PANSS-G were influenced by genotype, but changes in PANSS-N were independent of genotype. Patients receiving FA showed convergent changes in ventromedial prefrontal physiology, including increased task-induced deactivation, altered limbic connectivity, and increased cortical thickness | CTI: NCT01091506.  Six variants within folate-related genes: *MTHFR* (rs1801133), *MTR* (rs1805087), *FOLH1* (rs202676), *COMT* (rs4680), *DHFR* (rs2618372), and *GCH1* (rs8007267) were considered |
| Siscoe,  2017  (28272116) | 1/0 | 8 | 1/0 | ASD | Intervention group:  FA (L-methylfolate), Behavioural analysis | FA, 0.015 | 36 w | ABA, MOAS | Reduced aggression was noted with less impulsive behaviour, with an 8-point decrease in the MOAS between baseline and 9 months of follow-up | The patient is a child with ASD who was homozygous (TT) for the *MTHFR* 677C>T variant |
| Sun,  2016  (27338456) | 66/0 | 4.5 (1.2) | 54/12 | ASD | Intervention group:  FA, Structured teaching  Placebo group: Structured teaching | FA, 800 µg/d | 3 m | ATEC, PEP-3, ABC, CARS, Vit B12, FA, Homocysteine, GSH, GSSG | FA intervention improved ASD symptoms in sociability, cognitive verbal/preverbal, receptive, language, and affective expression and communication. In addition, FA treatment improved FA and homocysteine concentrations and normalized glutathione redox metabolism | The study was open-label and non-randomized |
| Bedson,  2014  (25052890) | 440/0 | 19-81  45 (13) | 160/280 | MDD | Intervention group:  FA  Placebo group:  NA | FA, 0.005 | 12 w | BDI-II, CGI, MADRS, UKU, MINI (ss), SF-12, EQ-5D, FA, Vit B12, Homocysteine | FA did not significantly improve any of these measures. This study did not provide evidence that FA is clinically effective in improving antidepressant treatment | CTI: ISRCTN37558856.  It has been suggested that methylfolate may be a better candidate instead of FA |
| Loria-Kohen,  2013  (23848107) | 24/0 | 24.2 (8.8) | NA | ED | Intervention group:  FA  Placebo group:  Crystalline cellulose, Lactose, Food colouring | FA, 0.01  Placebo, NA. | 6 m | Homocysteine, Serum folate, RBC folate, Vit B12, BMI, Haematocrit, Haemoglobin, ST, TMT, BDI-II | The intervention group significantly increased their serum and RBC folate levels and decreased their homocysteine levels (9.4 (2.4) μmol/l vs. 7.5 (1.7) μmol/l, *p*<0.01). In addition, the intervention group significantly improved most of their cognitive and depressive status test scores |  |
| Roffman,  2013  (23467813) | 140/0 | 18-68  45.6 (1.4) | 99/41 | SCZ | Intervention group:  FA, Vit B12  Placebo group:  NA | FA, 0.002  Vit B12, 400 μg/d | 16 w | PANSS, SANS, CDSS, RBC, folate levels, Homocysteine, Vit B12 | FA plus Vit B12 significantly improved negative symptoms significantly compared to placebo (group difference: -0.33 change in SANS per week; 95% CI, -0.62 to -0.05) when genotype was considered, but not when genotype was excluded. An interaction of the 484C>T variant of *FOLH1* (rs202676) with treatment was observed (*p*=0.02), with only patients homozygous for the 484T allele showing a significantly greater benefit with active treatment (-0.59 change in SANS per week, 95% CI, -0.99 to -0.18) | CTI: NCT00611806.  Patients were selected if their PANSS score was >60.  The study considered the influence of genetic variants of the *FOLH1*, *MTHFR*, *MTR*, and *COMT* genes |
| Hill,  2011  (21334854) | 32/0 | 46 | 26/6 | SCZ | Intervention group:  FA  Placebo group:  NA | FA, 0.002 | 12 w | SANS modified, PANSS, CDSS, GAF, QOL, NAART, CVLT, FAS letters, FAS animals, ST, Cognitive composite, Serum folate, RBC folate, Serum Vit B12, Plasma homocysteine | FA supplementation did not significantly affect negative symptoms compared to placebo; however, there was a significant *MTHFR* genotype × treatment effect on negative symptoms (*p*=0.01). In addition, negative symptoms were more likely to improve with increased serum folate in patients with at least one T allele (*p*=0.03) | The study analysed the *MTHFR* 677C>T variant |
| Christensen,  2011  (20805005) | 900/0 | 60-74  65.9 (4.2) | 358/  542 | Depressive symptoms | Intervention group:  FA, Vit B12  Placebo group:  NA | FA, 400 μg/d  Vit B12, 100 μg/d | 24 m | K-10, PHQ-9, Folate, Homocysteine | Intervention and placebo were applied to participants on antidepressant and no antidepressant treatment; and a mixed model repeated measures analysis of variance for reduction in depressive symptoms found no significant three-way interaction between supplement group and antidepressant use over time on the PHQ-9 (*p*=0.87) | Participants were selected if they scored >15 and <30 on the Kessler Distress 10 scale |

C: control; *COMT*: catechol-O-methyltransferase; CTI: Clinical Trial Identifier; d: day; *DHFR*: dihydrofolate reductase; F: female; *FOLH1*: folate hydrolase 1: g: gram; *GCH1*: GTP cyclohydrolase 1; m: months; M: male; *MTHFR*: methylenetetrahydrofolate reductase; *MTR*: 5-methyltetrahydrofolate-homocysteine methyltransferase; N: number of subjects; NA: not available; P: patient; PMID: PubMed identifier; w: week; y: year.

Age is presented as mean (SD) or range.

* Doses are given in g/d unless otherwise is specified.

**Phenotype/Disease:**

ADHD: attention deficit and hyperactivity disorder; ASD: autism spectrum disorders; ED: eating disorders; MDD: major depressive disorder; SCZ: schizophrenia.

**Nutraceutical supplements:**

FA: folic acid; Vit B12: vitamin B12.

**Measures:**

ABA: Applied Behavioural Analysis; ABC: Autism Behaviour Checklist; AISRS: Adult ADHD Investigator Symptom Report Scale; ASR: Adult Self-report; ATEC: Autism Treatment Evaluation Checklist; BDI: Beck Depression Inventory-II; BMI: body mass index; BRIEF-A: Behaviour Rating Inventory of Executive Function–Adult Version; CANTAB: Cambridge Neuropsychological Test Automated Battery; CARS: Childhood Autism Rating Scale; CBS-SR: Current Behaviour Scale–Self-report; CDSS: Calgary Depression Scale for Schizophrenia; CGI: Clinical Global Impression; CVLT: California Verbal Learning Test; EQ-5D: European Quality of Life – 5 dimensions; FA: folic acid; FAS: Verbal fluency tests; GAF: Global assessment of Function; GSH: reduced glutathione; GSSG: oxidized glutathione disulfide; HAM-A: Hamilton Anxiety Rating Scale; HAM-D: Hamilton Depression Rating Scale; K-10: Kessler Distress 10-Scale; MADRS: Montgomery-Asberg Depression Rating Scale; MCCB: MATRICS (Measurement and Treatment Research to Improve Cognition in Schizophrenia) Consensus Cognitive Battery; MINI: Mini International Neuropsychiatric Interview, suicidality subscale; MOAS: Modified Overt Aggression Scale; MRI: magnetic resonance imaging; NAART: North American Adult Reading Test; PANSS: Positive and Negative Syndrome Scale; PANSS-G: PANSS general score; PANSS-N: PANSS negative score; PANSS-T: PANSS total score; PANSS-P: PANSS positive score; PEP-3: Psychoeducational Profile-third edition; PHQ-9: Patient Health Questionnaire-9; QOL: quality of life; RBC: red blood cells; SANS: Scale for Assessment of Negative Symptoms; SANS modified: SANS minus the Attention subscale; SAS-SR: Social Adjustment Scale-Self Report; SF-12: General health – UK 12-item Short Form Health Survey; ST: Stroop colour-word interference Test; TMT: Trail Making Test; UKU: Udvalg for Kliniske Undersøgelser Side Effect Rating Scale; Vit B12: vitamin B12; WAIS-III: Wechsler Adult Intelligence Scale.

**Supplementary table 11**. Studies reporting results of Vitamin B12 (Vit B12) supplementation in psychiatric conditions.

| **First author,**  **publication year**  **(PMID)** | **Patient/Control Characteristics** | | | **Phenotype**  **/Disease** | **Study group**  **Nutraceutical supplementation** | **Nutraceutical information** | | **Measures** | **Findings** | **Additional information** |
| --- | --- | --- | --- | --- | --- | --- | --- | --- | --- | --- |
|  | N  P/C | Age (y)  in P/C | Sex  M/F |  |  | Doses (g/d)* | Treatment time |  |  |  |
| Roffman,  2013  (23467813) | 140/0 | 18-68  45.6 (1.4) | 99/41 | SCZ | Intervention group:  FA, Vit B12  Placebo group:  NA | FA, 0.002  Vit B12, 400 μg/d | 16 w | PANSS, SANS, CDSS, RBC folate, Homocysteine, Vit B12 | FA plus Vit B12 significantly improved negative symptoms compared to placebo (group difference: -0.33 change in SANS per week; 95% CI, -0.62 to -0.05) when genotype was considered, but not when genotype was not considered. An interaction of the 484C>T variant of *FOLH1* (rs202676) with treatment was observed (*p*=0.02), with only patients homozygous for the 484T allele showing a significantly greater benefit with active treatment (-0.59 change in SANS per week, 95% CI, -0.99 to -0.18) | CTI: NCT00611806.  Patients were selected if their PANSS score was >60. The study considered the influence of genetic variants of the *FOLH1*, *MTHFR*, *MTR*, and *COMT* genes |
| Kałużna-  Czaplińska,  2011  (21530806) | 30/21 | 4-11 | 27/3 (P)  20/1 (C) | ASD | Intervention group 1:  FA, Vit B6, Vit B12  Intervention group 2:  Vit B6, Vit B12  Control group | FA, 400 μg  Vit B6, 0.2  Vit B12, 1.2 μg | 3 m | Urinary homocysteine | Urinary homocysteine levels were significantly higher in ASD children before the intervention (2.41 (1.10)) compared to both vitamin supplementation (1.13 (0.44)) and non-ASD control children (1.33 (0.39)). The intake of vitamins B6 and B12 together with folic acid was found to be more effective in reducing urinary homocysteine levels than the intake of vitamins B6 and B12 alone |  |
| Christensen,  2011  (20805005) | 900/0 | 60-74  65.9 (4.2) | 358/542 | Depressive symptoms | Intervention group:  FA, Vit B12  Placebo group:  NA | FA, 400 μg/d  Vit B12, 100 μg/d | 24 m | K-10, PHQ-9, Folate, Homocysteine | Intervention and placebo were applied to participants on antidepressant and no antidepressant treatment; a mixed model repeated measures analysis of variance for reduction in depressive symptoms found no significant three-way interaction between supplement group and antidepressant use over time on the PHQ-9 (*p*=0.87) | Participants were selected if they scored >15 and <30 on the Kessler Distress 10 scale |
| Kuo,  2009  (19892219) | 1/0 | 31 | 1/0 | SCZ-like psychotic episode precipitated by cobalamin deficiency | Intervention:  Vit B12 (mecobalamin) | Vit B12, 0.001 | 2 w | Vit B12 | After 2 w of treatment, his confusion resolved and he was discharged to outpatient follow-up. After 2 m of treatment, his cobalamin level returned to 277 pg/ml. Psychotic symptoms did not recur within 1 year of outpatient follow-up | This study is a case report. Prior to treatment, cobalamin levels were of 136 pg/ml (reference range: 211-911 pg/ml) |

C: control; *COMT*: catechol-O-methyltransferase; CTI: Clinical Trial Identifier; d: day; F: female; *FOLH1*: folate hydrolase 1; g: gram; m: months; M: male; *MTHFR*: methylenetetrahydrofolate reductase; *MTR*: 5-methyltetrahydrofolate-homocysteine methyltransferase; N: number of subjects; NA: not available; P: patient; PMID: PubMed identifier; w: week; y: year.

Age is presented as mean (SD) or range.

* Doses are given in gram/day unless otherwise specified.

**Phenotype/Disease:**

ASD: autism spectrum disorders; SCZ: schizophrenia.

**Nutraceutical supplements:**

FA: folic acid; Vit B12: vitamin B12; Vit B6: vitamin B6.

**Measures:**

CDSS: Calgary Depression Scale for Schizophrenia; K-10: Kessler Distress 10-Scale; PANSS: Positive and Negative Syndrome Scale; PHQ-9: Patient Health Questionnaire-9; RBC: red blood cells; SANS: Scale for Assessment of Negative Symptoms; Vit B12: vitamin B12 (cobalamin).

**Supplementary Table 12**. Studies reporting results of Vitamin E (Vit E) supplementation in psychiatric conditions.

| **First author,**  **publication year**  **(PMID)** | **Patient/Control Characteristics** | | | **Phenotype**  **/ Disease** | **Study group**  **Nutraceutical supplements** | **Nutraceutical information** | | **Measures** | **Findings** | **Additional information** |
| --- | --- | --- | --- | --- | --- | --- | --- | --- | --- | --- |
|  | N  P/C | Age (y)  in P/C | Sex  M/F |  |  | Doses in g/d* | Treatment time |  |  |  |
| Pawełczyk,  2021  (33684737) | 71/0 | 23.2 (4.8) | 42/29 | FES | İntervention group:  PUFA  Vit E  Placebo group:  Olive oil  Vit E | PUFA, 2.2  Vit E, 0.2%  Olive oil, NA | 26 w | MetS frequency, MetS parameters, PANSS | Vit E was not the nutraceutical target of the study | Vit E was included in both study groups to prevent oxidation of fatty acids |
| Szeszko,  2021  (33444934) | 46/0 | 21.5 (5.3) | 35/11 | ROP | Intervention group:  PUFA  Vit E  Placebo group:  soybean/corn blend | PUFA, 1.14  Vit E, 0.004  Soybean/corn blend, 2 | 16 w | BPRS, MCCB, AA | Vit E was not the nutraceutical target of the study | CTI: NCT01786239.  At baseline, both higher DHA and EPA correlated significantly with better social cognition, and higher AA correlated significantly with hostility/ uncooperativeness, after controlling for several covariates. After 16 w of treatment, the intervention group showed a significant increase in social cognition compared to the placebo group. DHA correlated significantly with social cognition at 16 w |
| Robinson,  2019  (30241990) | 50/0 | 15-40 | Mostly M | ROP | Intervention group:  PUFA  Vit E  Placebo group:  soybean/corn blend | PUFA, 1.14  Vit E, 0.002  Soybean/corn blend, 2 | 16 w | BPRS, SANS, CGI, weight, BMI, HDL-c, LDL-c, T-c, TG, HbA1c, fasting glucose | Vit E was not the nutraceutical target of the study | CTI: NCT01786239.  Longitudinal analysis of the BPRS total score showed a trend level (*p*=0.083) treatment effect in favour of PUFA treatment. In the subgroup that did not receive lorazepam, the treatment effect on the total BPRS score was significant (*p*=0.041), and factor score analyses revealed a significant reduction in depression-anxiety with PUFA but no change with placebo (treatment by time interaction, p = 0.018) |
| Qiao,  2018  (28830741) | 50/0 | 18-60 | 30/20 | SCZ | Intervention group:  PUFA  Placebo group:  Vit E | PUFA, 0.9  Vit E, 0.01 | 12 w | PANSS, CGI, MOAS | Vit E was not the nutraceutical target of the study | PANSS and CGI scores decreased at w 4, 8, and 12, but no differences were found between the two groups. MOAS scores decreased significantly at w 4, 8, and 12. At w 12, MOAS scores in the PUFA group were significantly lower than in the placebo group (t=−2.40, *p*<0.05) |
| Kean,  2017  (27921139) | 144/0 | 8.7 | 123/21 | Children and adolescents with clinical and  subclinical symptoms of ADHD | Intervention group:  PUFA  Vit E  Placebo group:  Olive oil  Lecithin  Coconut oil  β-carotene | Treatment:  ETA, 0.05 (including PUFA 0.0128  Olive oil, 0.1)  Vit E, 0.00225  Placebo:  Olive oil, 0.0355  Lecithin, 0.112  Coconut oil, 0.012  30% β-carotene, 0.0005 | 14 w | CPRS, TOVA, COMPASS, BRUMS, EEG | Vit E was not the nutraceutical target of the study | CTI: ANZCTRN12610000978066.  Parental reports of hyperactivity, inattention, and impulsivity did not differ between the intervention and placebo groups.  Post hoc analyses showed that children who did not meet criteria for combined hyperactivity and inattention showed significant improvements in hyperactivity (*p*=0.04), attention (*p*=0.02), learning (*p*=0.05), and likelihood of ADHD (*p*=0.04). Significant improvements in recognition memory were observed between baseline and 8 w in the intervention group versus placebo (*p*=0.02, d=0.56); this difference was not maintained at 14 w |
| Chhetry,  2016  (26802812) | 16/12 | 22-50 | 5/11 (P)  5/7 (C) | MDD  C | Intervention group:  PUFA  Vit E  Control group | PUFA, 2.4  Vit E, 20 IU/d | 6 w | MRI (FA), HDRS, Gas chromatography | Vit E was not the nutraceutical target of the study | There was no placebo group.  Increases in plasma DHA% (but not EPA% or AA%) after intervention predicted increases in FA in MDD but not in C (in a clustered range *p*<0.001, peak t-score=8.10, *p*=0.002). There was a trend for greater change in FA in MDD responders than non-responders (t=-1.874, *p*=0.08). Decreased depression severity predicted increased FA in the left corticospinal tract and superior longitudinal fasciculus (cluster level *p*<0.001, peak t-score=5.04, *p*<0.001). Increased FA% correlated with increased DHA% and decreased depression severity after PUFA supplementation |
| Pawełczyk,  2016  (26679763) | 71/0 | 23.3 (4.8) | 42/29 | FEP | Intervention group: PUFA  Vit E  Placebo group:  Olive oil  Vit E | PUFA, 2.2  Vit E, 0.2% | 26 w | PANSS, CDSS | Vit E was not the nutraceutical target of the study | Significant improvements in symptom severity were achieved more frequently in the PUFA group than in the placebo group (69.4% vs 40.0%; *p*=0.017). The PUFA intervention was associated with improvements in general psychopathology (*p*=0.009), depressive symptoms (*p*=0.006), functioning (*p*=0.01), and clinical global impression (*p*=0.047) |
| Bošković,  2016  (25056532) | 34/0 | 50.2 (9.2) | 17/17 | SCZ | Intervention Group1:  Vit E  Intervention Group2:  PUFA  Intervention Group3: Vit E + PUFA  Placebo group:  Lactose | Vit E, 1 200 IU/d  PUFA, 1.098  Lactose, 0.5 | 4 m | SOD, GpX, GR, CAT, GSH, GSSG, PC, MDA, NO_3_, NO_2_, NAD, AD, DA, 5-HT, PANSS, AIMS, SAS, BARS | Vit E was not the nutraceutical target of the study | Patients receiving PUFA showed a significant increase in blood GSH levels compared to placebo (*p*=0.044). Patients receiving Vit E showed a significant decrease in GSSG and less motor retardation. No significant effect on symptom severity was observed |
| Pawełczyk,  2015  (25934131) | 82/0 | 16-35 | NA | FES | Intervention Group:  PUFA  Vit E  Placebo group:  Olive oil  Vit E | PUFA, 3.08  Vit E, 0.2% | 26 w | PANSS, CDSS, GAF,  CGI, My thoughts and feelings questionnaire (insight) | Vit E was not the nutraceutical target of the study | CTI: NCT02210962.  The study only shows the rationale, design, and methods of the clinical trial |
| Amminger,  2015  (25585167) | 81/0 | 16.4 (2.1) | 27/54 | UHR | Intervention Group:  PUFA  Vit E  Placebo group:  Coconut oil  Vit E | PUFA, 1.18  Vit E, 0.0076  Coconut oil, NA | 12 w | GAF, PANSS, MADRS, RBC membrane fatty acid composition | Vit E was not the nutraceutical target of the study | Higher RBC membrane alpha-lipoic acid levels and more severe negative symptoms at baseline predicted subsequent functional improvement in the treatment group, whereas less severe positive symptoms and lower baseline functioning were predictive of the placebo group |
| Smesny,  2014  (23478748) | Cross-section study (CSS)  80/0  Longitudinal  Study (LS)  65/0 | 13-25  16.5 (2.2) | CSS 26/54  LS 20/45 | UHR | Intervention group 1:  PUFA  Vit E  Placebo group:  Coconut oil  Fish oil  Vit E | PUFA, 1.18  Vit E, 0.0076  Coconut oil, NA  Fish oil, 1% | 12 w | PANSS, GAF, Transition to psychosis, RBC-PUFA, inPLA2 activity, GSHt, GSH, GSSG, GSH/GSSG | Vit E was not the nutraceutical target of the study | CTI: NCT00396643.  The levels of membrane ω-3 and ω-6 PUFAs and intracellular PLA_2_ were significantly associated. Some of the significant associations (i.e., long-chain ω-6 PUFAs, arachidonic acid) with intracellular PLA_2_ activity were in opposite directions in individuals who did (a positive correlation) and who did not (a negative correlation) transition to psychosis.  Supplementation with ω-3 PUFA resulted in a significant decrease in in PLA2 activity |
| Bentsen,  2013  (24346133) | Group 1: 25/0  Group 2: 28/0  Group 3: 33/0  Group 4: 18/0 | 18-39  Group 1: 28.3 (5.8)  Group 2: 28.6 (6.3)  Group 3:  25.7 (5.4)  Group 4: 27.6 (7.1) | Group 1: 17/8  Group 2: 14/14  Group 3: 20/13  Group 4: 12/6 | SCZ, SCZA, SCZP | Group 1:  Placebo PUFA & Placebo Vitamin  Group 2:  Placebo PUFA & Active Vit A + Vit E  Group 3:  Active PUFA & Placebo Vit  Group 4:  Active PUFA &  Active Vit A + Vit E | PUFA, 2  Vit E, 0.364  Vit C, 1  Placebo PUFA: paraffin, 0.5  Placebo Vit: dicalcium phosphate | 16 w | PANSS, USERS, RBC-PUFA, ARA, Lipid-adjusted s-alpha-tocopherol | When given separately, PUFA and vitamins  increased dropout rates, whereas when combined they did not differ from placebo.  Vitamins alone worsened the course of psychotic symptoms (*p*=0.005, d=0.37). PUFA & vitamins neutralized the adverse effect on psychosis (interaction *p*=0.02, d=0.31) | The PUFA capsules also contained Vit E at a total dose of 0.004 g/d.  In patients with low PUFA levels, PUFA supplementation alone impaired the total PANSS score (*p*=0.03, d=0.29) and psychotic symptoms (*p*=0.003, d=0.40). No significant effects of treatment on PANSS scales were observed in patients with high PUFA levels |
| Amminger,  2013  (23870722) | 15/0 | 13-25  16.2 (2.1) | 1/14 | BPD | Intervention Group:  PUFA  Vit E  Placebo group:  Coconut oil | PUFA, 1.2  Vit E, 0.0076  Coconut oil, NA | 12 w | PANSS, MADRS, GAF, USERS, PUFA | Vit E was not the nutraceutical target of the study | At baseline, RBC-PUFA correlated with the GAF score (r=0.54, *p*=0.04), the  PANSS negative score (r=-0.74, *p*=0.002), and the  PANSS total score (r=-0.55, *p*=0.04). Intervention group significantly improved functioning and reduced psychiatric symptoms |
| Meyer,  2013  (23733443) | 95/0 | 18-75 | NA | MDD | Intervention Group:  PUFA  Vit E  Placebo group:  Olive oil | PUFA, 2.6  Vit E, 0.010  olive oil (placebo; matched for Vit E content) | 16 w | HDRS, DHA, RBC-PUFA | Vit E was not the nutraceutical target of the study | No significant change in the mean depression scores was reported between the intervention and placebo groups; however, there was a significant correlation between change in RBC-PUFA and change in depression scores (r=-0.51, *p*<0.05).  RBC-PUFA content increased in the intervention group but not in the placebo group. (*p*<0.05) |
| Politi,  2008  (18760197) | 19/0 | 18-40  28.9 (4.9) | 15/4 | Severe ASD | Intervention Group:  PUFA  Vit E | PUFA, 0.93  Vit E 0.005 | 6 w | Rossago Behavioural Checklist | Vit E was not the nutraceutical target of the study | No significant improvements in the severity and the frequency of problem behaviours were observed either during the treatment or in the post-treatment period. No effect on the number of episodes and severity of behavioural abnormalities was observed |
| Germano,  2007  (17539477) | 31/36 | 8.4 (2.8) | 28/3 | ADHD | Intervention group:  PUFA  Vit E  Control group | PUFA, 2.247/10 kg  Vit E, NA | 8 w | AA/EPA, Haematological parameters, Conners scale short version (by the parents and teaching) | Vit E was not the nutraceutical target of the study | Inattention score decreased after the treatment period in ADHD patients (from 19 (4.2) to 13.9 (4.0), *p*=0.002) as well as the hyperactivity score (from 20 (4.2) to 15.5 (4.4), *p*=0.007). After the treatment period, the AA/EPA ratio also decreased (from 41.1 (21.05) to 4.1 (3.26), *p*=0.001).  No information is provided on the control group |

C: control; CTI: Clinical Trial Identifier; d: day; ES: Effect size; F: female; g: gram; IU: international units; m: month; M: male; N: number of subjects; NA: not available; P: patient; PMID: PubMed identifier; w: week; y: year.

Age is presented as mean (SD) or range.

* Doses are given in gram/day unless otherwise specified.

**Phenotype/Disease:**

ADHD: attention deficit hyperactivity disorder; ASD: autism spectrum disorder; BPD: borderline personality disorder; FEP: first-episode psychosis; FES: First-Episode Schizophrenia; MDD: major depressive disorder; ROP: recent onset psychosis; SCZ: schizophrenia; SCZA: schizoaffective disorder; SCZP: schizophreniform disorder; UHR: Ultra-high risk for psychosis.

**Nutraceutical supplements:**

ETA: eicosatetraenoic acid; PUFA: PolyUnsaturated Fatty Acid; Vit A: vitamin A; Vit C: vitamin C; Vit E: vitamin E.

**Measures:**

5-HT: serotonin; AA: arachidonic acid; AIMS: abnormal involuntary movement scale; AD: adrenaline; ARA: arachidonic acid; BARS: Barnes akathisia rating scale; BMI: Body Mass Index; BPRS: Brief Psychiatric Rating Scale; BRUMS: Brunel Mood Scale for adolescents; CAT: catalase; CDSS: Calgary Depression Scale for Schizophrenia; CGI: Clinical Global Impression Scale; COMPASS: Computerized Mental Performance Assessment System; CPRS: Conners Parent Rating Scales; DA: dopamine; DHA: Docosahexaenoic Acid; EEG: electroencephalography; EPA: Eicosapentaenoic Acid; FA: Fractional anisotropy; GAF: Global Assessment of Functioning; GpX: glutathione peroxidase; GR: glutathione reductase; GSH: reduced glutathione; GSHt: total glutathione; GSSG: oxidized glutathione; HDL-c: High Density Lipoprotein-cholesterol; HDRS: Hamilton Depression Rating Scale; Hb1ac: haemoglobin 1ac; inPLA2: intracellular phospholipase A2; LDL-c: Low Density Lipoprotein-cholesterol; MADRS: Montgomery-Asberg Depression Rating Scale; MCCB: MATRICS (Measurement and Treatment Research to Improve Cognition in Schizophrenia) Consensus Cognitive Battery; MDA: malondialdehyde; MetS: Metabolic Syndrome; MOAS: Modified Overt Aggression Scale; MRI: Magnetic Resonance Imaging; NAD: noradrenaline; NO2: nitrites; NO3: nitrates; PANSS: Positive and Negative Syndrome Scale; PC: protein carbonyls; PUFA: PolyUnsaturated Fatty Acid; RBC-PUFA: red blood cells - PUFA; SANS: Schedule for Assessment of Negative Symptoms; SAS: Simpson Angus scale; SOD: superoxide dismutase; T-c: Total-cholesterol; TG: triglycerides; TOVA: Test of Variables of Attention; USERS: Udvalg for Kliniske Undersøgelser Side Effect Rating Scale.

**Supplementary table 13**. Studies reporting results of Vitamin A (Vit A) supplementation in psychiatric conditions.

| **First author,**  **publication year**  **(PMID)** | **Patient/Control Characteristics** | | | **Phenotype**  **/Disease** | **Study group**  **Nutraceutical supplements** | **Nutraceutical information** | | **Measures** | **Findings** | **Additional information** |
| --- | --- | --- | --- | --- | --- | --- | --- | --- | --- | --- |
|  | N  P/C | Age (y)  in P/C | Sex  M/F |  |  | Doses in IU | Treatment time |  |  |  |
| Lai,  2021  (33328600) | 138/0 | 4.2 (1.2) | 118/20 | ASD | Intervention group 1:  Behavioural therapy + Vit A recommended intake  Intervention group 2:  Behavioural therapy + Vit A weekly intake  Intervention group 3:  Behavioural therapy | Group 1:  Vit A, single supplement of  200000 IU  Group 2:  Vit A,  50000 IU per week for 11w + 3 000 IU/d for 13w | 24 w | Anthropometric measures, SRS score  Serum retinol/OXT,  RARs and CD38 gene expression in PBMC | The weekly program increased Vit A levels better than the standardized program and significantly decreased SRS scores.  There was also a significant correlation between changes in Vit A levels, changes in the RARβ-CD38-OXT axis, and social functioning in children with ASD | CTI: ChiCTR-ROC-14005442.  This study compared ASD patients with reduced and normal Vit A levels. Those with Vit A deficiency were divided into two groups, Group 1 treated with a standardized dosing regimen and Group 2 treated with weekly supplementation |
| Guo,  2018  (29122693) | 33/32 | 5.1 (1.3) (P)  5.2 (0.9) (C) | 28/5 (P)  26/6 (C) | ASD | Intervention group:  Vit A  Control group:  NA | Vit A, 200000 IU/once | 24 w | ABC, CARS, GDS,  serum retinol, 5-HT, mitRNA expression, levels of retinoic acid receptors and tryptophan hydroxylase 1 | 5-HT levels were significantly higher, and retinol levels were significantly lower in children with severe ASD compared with those with mild to moderate ASD. Serum retinol levels in children with ASD increased significantly after vitamin supplementation (from 0.54 (0.17 μmol/L to 0.79 (0.16) μmol/L). Supplementation significantly improved patient’s symptoms as measured by CARS scores before and after intervention | CTI: ChiCTR-ROC-14005442.  Patients received only 1 dose of treatment, but were followed for over a period of 6 months |
| Liu,  2017  (28938872) | 64/0 | 1-8 | 55/9 | ASD | Intervention group:  Vit A  No placebo/control  group was formed | Vit A, 200000 IU/once | 24 w | ABC, CARS, SRS, plasma retinol test, CD38 mRNA, RORA mRNA | Both CD38 and RORA mRNA levels were significantly increased in ASD  after supplementation. No significant differences were found in ASD  symptoms (ABC, CARS, and SRS scales) before and after supplementation | CTI: ChiCTR-ROC-14005442.  As all participants were Vit A deficient, no placebo group was created |

C: control; CTI: Clinical Trial Identifier; d: day; F: female; g: gram; IU: international units; M: male; N: number of subjects; NA: not available; P: patient; PMID: PubMed identifier; w: week; y: year.

Age is presented as mean (SD) or range.

**Phenotype/Disease:**

ASD: autism spectrum disorder.

**Nutraceutical supplements:**

Vit A: vitamin A.

**Measures:**

5-HT: 5-hydroxytryptamine (serotonin); ABC: Autism Behaviour Checklist; CARS: Childhood Autism Rating Scale; GDS: Gesell Developmental Scale; mitRNA: mitochondrial RNA; OXT: oxytocin; PBMC: peripheral blood mononuclear cells; RAR: retinoic acid receptors; RORA: acid-related orphan receptor alpha; SRS: Social Responsiveness Scale.

**Supplementary table 14**. Studies reporting results of Vitamin C (Vit C) supplementation in psychiatric conditions.

| **First author,**  **publication year**  **(PMID)** | **Patient/Control Characteristics** | | | **Phenotype**  **/Disease** | **Study group**  **Nutraceutical supplements** | **Nutraceutical information** | | **Measures** | **Findings** | **Additional information** |
| --- | --- | --- | --- | --- | --- | --- | --- | --- | --- | --- |
|  | N  P/C | Age (y)  in P/C | Sex  M/F |  |  | Doses in g/d* | Treatment time |  |  |  |
| Zhang,  2023  (37252140) | 44/0 | 28.3 (4.4) | 23/21 | SCZ | Intervention group 1:  Vit C  Intervention group 2:  Topiramate | Vit C,  0.1 - 0.2  Topiramate,  0.1 - 0.2 | 12 w | PANSS, BMI, WHR, cholesterol, TG, HDL-c, LDL-c | Although BMI levels were not significantly different between the two groups, the change in BMI from week 4 to the end of the study was significantly lower in the topiramate group than in the vitamin C group | This study was conducted in patients with schizophrenia treated with olanzapine.  The results observed that the TG levels had a significantly higher level in the Vit C group than in the topiramate group over time |
| Wang,  2013  (23885048) | 52/0 | 66.2 (15) | 28/24 | Acutely hospitalized patients with a high prevalence of hypovitaminosis C and D | Intervention group 1:  Vi C  Intervention group 2:  Vit D | Vit C, 1  Vit D, 5000 IU/d | 10 d | POMS-B, DT, plasma total Vit C, plasma 25-hydroxyvitamin D, PTH, CRP | Vit C was associated with a 71% and 51% reduction in mood disturbance and psychological distress, respectively. Vit D showed insignificant effects on mood and distress | CTI: NCT01630720.  Baseline plasma PTH concentrations decreased after Vit C but not Vit D supplementation. Correlation between improvement in total mood disturbance score and increase in total plasma Vit C concentration was significant |
| Raz,  2009  (19364294) | 63/0 | 7-13  10.5 (1.5) | 38/25 | ADHD | Intervention group:  LA, α-LA, mineral oil,  α-tocopherol  Placebo group:  Vit C | LA, 0.48  α-LA, 0.12  Mineral oil,  0.095  α-tocopherol,  0.005  Vit C, 1 | 7 w | TOVA, CPRS, T-c,  HDL-c, LDL-c, triglycerides, iron, ferritin,  Vit B12, haemoglobin | Vit C was not the target  of the current trial | Both treatments improved some of the symptoms, but no significant differences were found between the groups in any of the treatment effects |

C: control; d: day; F: female; g: gram; IU: international units; M: male; N: number of subjects; P: patient; PMID: PubMed identifier; w: week; y: year.

Age is presented as mean (SD) or range.

* Doses are given in g/d unless otherwise is specified

**Phenotype/Disease:**

ADHD: attention deficit hyperactivity disorder; SCZ: schizophrenia

**Nutraceutical supplements:**

α-LA: α-linoleic acid; LA: Linoleic acid; Vit: vitamin

**Measures:**

BMI: body mass index; CPRS: Conner’s Parent Rating Scale; CRP: C-reactive protein; DT: Distress Thermometer; HDL-c: high-density lipoprotein cholesterol; LDL-c: low-density lipoprotein cholesterol; PANSS: positive and negative syndrome scale; POMS-B: Profile of Mood States; PTH: Parathyroid Hormone; T-c: total cholesterol; TG: triglycerides; TOVA: Test of Variables of Attention; WHR: waist hip ratio.

**Supplementary table 15**. Studies reporting results of Vitamin B3 (Vit B3) supplementation in psychiatric conditions.

| **First author,**  **publication year**  **(PMID)** | **Patient/Control Characteristics** | | | **Phenotype**  **/Disease** | **Study group**  **Nutraceutical supplements** | **Nutraceutical information** | | **Measures** | **Findings** | **Additional information** |
| --- | --- | --- | --- | --- | --- | --- | --- | --- | --- | --- |
|  | N  P/C | Age (y)  in P/C | Sex  M/F |  |  | Doses (g/d) | Treatment time |  |  |  |
| Raison,  2023 (37651119) | 104/0 | (21-65)  41.1 (11.3) | 52/52 | MDD | Intervention group: Psilocybin  Placebo group:  Vit B3 | Psilocybin, 0.025  Vit B3, 0.1 | 6 w | MADRS, Sheehan Disability Scale | Vit B3 was not the target of the study | CTI: NCT03866174.  Vit B3 was used as an active placebo, which produces an acute physiological response (flushing) thought to aid in blinding.  Psilocybin treatment was associated with clinically significant and sustained reductions in depressive symptoms and functional disability, with no serious adverse events |

C: control; CTI: Clinical Trial Identifier; d: day; F: female; g: gram; M: male; N: number of subjects; P: patient; PMID: PubMed identifier; w: week; y: year.

Age is presented as mean (SD) or range.

**Phenotype/Disease:**

MDD: major depressive disorder.

**Nutraceutical supplements:**

Vit B3: vitamin B3, also known as niacin.

**Measures:**

MADRS: Montgomery-Asberg Depression Rating Scale.

**Supplementary table 16**. Studies reporting results of supplementation of two or more nutraceutical components in psychiatric conditions.

| **First author,**  **publication year**  **(PMID)** | **Patient/Control Characteristics** | | | **Phenotype**  **/Disease** | **Study group and**  **Nutraceutical supplements** | **Nutraceutical information** | | **Measures** | **Findings** | **Additional information** |
| --- | --- | --- | --- | --- | --- | --- | --- | --- | --- | --- |
|  | N  P/C | Age (y)  in P/C | Sex  M/F |  |  | Doses in g/d, %/d, or IU/d * | Treatment time |  |  |  |
| Russell,  2023  (37424414) | 148/0 | 45.4 (11.1) | 52/96 | BD,  BD + PTSD | Intervention group 1:  NAC + placebo  Intervention group 2:  NAC, ALA, ALCAR, ubidecarenone, Mg, Vit E, Vit B1, Vit B2, Vit B3, Vit B5, Vit B6, FA, Vit B12, Ca, Vit A, Vit D3, Vit B7  Placebo group:  NA | NAC, 1  ALA, 0.075  Ubidecarenone, 0.075  Mg, 0.032  Vit E, 0.020  ALCAR, 0.5  Vit B1, 0.05  Vit B2, 0.05  Vit B3, 0.1  Vit B5, 0.045  Vit B6, 0.0411  FA, 400 μg/d  Vit B12, 400 μg/d  Ca, 0.121  Vit A, 450 μg/d  Vit D3, 6.25 μg/d  Vit B7, 300 μg/d | 16 w | MADRS, BDRS, YMRS, SOFAS | There were no differences between BD and BD + PTSD on measures of depression, mania, and functioning over time | CTI: ACTRN12612000830897 |
| Weggen,  2021  (34075811) | 13/35 | 18-35  23 (4) (P)  23 (4) (C) | 4/9 (P)  11/24 (C) | PTSD | Intervention group:  ALA, Vit C, Vit E  Control group:  microcrystalline cellulose | ALA, 0.4  Vit C, 0.5  Vit E, 0.2  Microcrystalline cellulose, NA | 1.5 h  2 h | Upper and lower limb vascular functions  Heart rate | After acute supplementation, arm, but not leg, microvascular function was improved and sympathetic predominance was reduced to the point that the previous difference between the PTSD group and C was no longer significant |  |
| Reininghaus,  2020  (33171595) | 82/0 | 20-55  41.6 (12.9) | 18/64 | MDD | Intervention group:  Probiotics^§^  Vit B7, Common horsetail, Fish collagen, Keratin plus matrix  Placebo group:  Vit B7, Common horsetail, Fish collagen, Keratin plus matrix | Probiotics, ≥7.5 billion organisms  Vit B7, 0.125  Horsetail, 0.03  Collagen, 0.03  Keratin, 0.03 | 28 d | HDRS, BDI-II, SCL-90, GSI, PSDI, PST, MSS, GLQI, BMI, Zonulin, Microbiome analysis (16S), Waist to hip ratio. | Vit B7 was not the target of the study | CTI: NCT03300440.  Probiotic intervention did not differ from placebo in clinical outcome measures. *Ruminococcus gauvreauii* and *Coprococcus* 3 were more abundant, and β-diversity was higher in the probiotic group after 28 days of treatment. KEGG analysis showed increased inflammation-regulating and metabolic pathways in the intervention group |
| van der Burg,  2020  (31555976) | 96/0 | 18-70  43.7 (12.4) | 24/72 | MDD | Intervention group:  SAMe, Folinic Acid, Vit B12, PUFA, 5-HTP, Zn, Vit B6, Vit C, Mg  Placebo group:  NA | SAMe: 0.8  Folinic Acid: 0.5  Vit B12: 0.2  PUFA, 1.656  5-HTP: 0.2  Zinc: 0.03  Vit B6: 0.1  Vit C: 0.06  Mg: 0.04 | 8 w | RBC-PUFA, folate, Vit B12, Zn, Homocysteine, Brain-derived neurotrophic factor | RBC membrane concentrations of PUFA increased in response to treatment and were significantly correlated with a reduction in depressive symptoms during active treatment (*p*<0.05). Higher baseline omega-6 fatty acid levels correlated with a reduction in depression in the active treatment group (*p*=0.011) | No other biomarkers were associated with a reduction in depressive symptoms |
| [Bot](https://pubmed.ncbi.nlm.nih.gov/?term=Bot+M&cauthor_id=30835307),  2019  (30835307) | 1 025/0 | 46.5 | 772/253 | Elevated depressive symptoms and no MDD episode in the past 6 months | Intervention group 1:  PUFA, Se, Folic acid, Vit D3, Ca, Behavioural therapy  Intervention group 2:  PUFA, Se, Folic acid, Vit D3, Ca  Intervention group 3:  Placebo, Behavioural therapy  Intervention group 4:  Placebo | PUFA, 1.412  Se, 30 μg  Folic acid, 400 μg  Vit D3, 20-μg  Ca 0.1 | 12 m | 12-month cumulative onset of an episode of MDD,  PHQ-9, IDS30-SR, GAD-7, EQ-5D-5L, TEFQ-R18, GA^2^LEN-FFQ, SQUASH, Body weight perception | This study showed that none of the treatment strategies affected the incidence of MDD during the 12 months of follow-up. The odds ratio (OR) was 1.06 (95%CI, 0.87-1.29) for supplements, 0.93 (95%CI, 0.76-1.13) for therapy, and 0.93 (95%CI, 0.76-1.14) for their combination; *p*=0.48 for interaction | CTI: NCT02529423 |
| Allot,  2019  (30771856) | 120/0 | 19.9 (2.7) | 68/52 | FEP | Intervention group:  Folic acid, Vit B6, Vit B12  Placebo group  NA | Folic acid, 0.005  Vit B6, 0.05  Vit B12, 0.0004 | 12 w | Homocysteine, PANSS, BPRS-E, SANS, CDSS, YMRS, CGI, GAF, Composite neurocognition (11 test battery) | B-vitamin supplementation reduced homocysteine levels (*p*=0.003, effect size=-0.65). B-vitamin supplementation had no significant effect on PANSS score (*p*=0.749) or composite neurocognition (*p*=0.785). Being female and having affective psychosis were associated with improved neurocognition in selected domains after B-vitamin supplementation | CTI: NCT00202280 |
| Adams,  2018  (29562612) | 67/50 | 3-58  11.6 (8.6) (P)  12.2 (7.5) (C) | 96/21 | ASD | Intervention group 1:  32 supplements^❡^  Intervention group 2:  Non-treatment  Control group | Vitamin/mineral, Essential fatty acids, Epsom salt baths, Carnitine and Digestive enzymes (Check study tables) | 12 m | ADOS, RIAS, CARS-2, SAS-Pro, VABS-II, PDD-BI, ATEC, ABC, SRS, SSP, PGI-2, 6-GSI, Handgrip Strength, Vit/mineral, PUFA, Homocysteine, Carnitine, Digestive enzymes | Increase in non-verbal IQ and a significant 18-month increase in developmental ability in communication, daily living skills, and social skills in the treatment group compared to the non-treated group. The treatment group had significantly greater increases in PUFA, carnitine and Vit A, B2, B5, B6, B12, folic acid and CoQ | CTI: NCT02059577.  Based on semi-blinded assessment, the treatment group had significantly greater improvement in ASD symptoms and developmental age compared to the non-treatment group.  Parents reported that the vitamin/mineral supplements, essential fatty acids, and healthy gluten-free, casein-free, soy-free diet were most beneficial |
| Kałużna-  Czaplińska,  2017  (28608247) | 236/0 | 3-16 | 189/47 | ASD | Intervention group 1:  (ASD) Vitamins B & Mg  Placebo group 1:  (ASD) PUFA  Intervention group 2:  (AS) Vitamins B & Mg  Placebo group 2:  (AS) PUFA  Intervention group 3:  (ASD with psych. ret.) Vitamins B & Mg  Placebo group 3: (ASD with psych. ret.) PUFA | Vitamins B, NA  Mg, NA  PUFA, NA | NA | Tryptophan, BMI, Specific gravity, pH, Leukocytes, Nitrite, Protein, Glucose, Ketone, Urobilinogen, Bilirubin | Supplementation with B vitamins and Mg influenced tryptophan levels. No correlation was found between BMI and tryptophan levels | No information is provided on the types of B vitamins |
| Dean,  2015  (25295681) | 225/0 | >18 | NA | BD | Intervention group 1:  NAC  Intervention group2:  CT  Placebo group:  NA | NAC, 0.002  CT:  (NAC, 0.002,  ALCAR, 0.001,  CoQ, 0.2,  Vit E, 0.04032,  ALA, 0.15,  Mg, 0.064,  Vit B1, 0.1  Vit B2, 0.1  Vit B3, 0.2  Vit B5, 0.1  Vit B6, 0.1  Vit B9, 800 μg  Vit B12, 800 μg  Vit C, 0.242  Vit A, 900 μg  Vit D3, 12.5 μg  Vit H, 600 μg) | 16 w | MADRS, BDRS, HDRS, YMRS, LIFE-RIFT, SOFAS, Q-LES-Q (sf), CGI-BP, PGI | Only the design and the rationale of the study are reported | CTI: ACTRN12612000830897 |
| Adams,  2011  (22151477) | 141/44 | 10.6 (5.5) | 125/16 | ASD | Intervention group 1:  Vit A, Vit C, Vit D3, Vit E, MT, Vit B1, Vit B2, Vit B3, Vit B5, Vit B6, Vit B12, Folic acid, Folinic acid, Biotin, Choline, Inositol, MC, CoQ, NAC, Ca, Cr, Iron, Li, Mg, Mn, Mo, K, Se, S, Zn  Control group:  beta-carotene, citric acid, and a proprietary blend of natural plant-based flavors to create a vitamin-like after-taste, xanthum | Vit A, 1 000 IU  Vit C, 0.6  Vit D3, 300 IU  Vit E, 150 IU  MT, 0.07  Vit B1, 0.02  Vit B2, 0.02  Vit B3, 0.015/0.010  Vit B5, 0.015  Vit B6, 0.040  Vit B12, 500 μg  Folic acid, 100 μg  Folinic acid, 550 μg  Biotin, 150 μg  Choline, 0.25  Inositol, 0.1  MC, 0.0036  CoQ, 0.05  NAC, 0.05  Ca, 0.1  Cr, 70 μg  I, 100 μg  Li, 500 μg  Mg, 0.1  Mn, 0.003  Mo, 150 μg  K, 0.05  Se, 22 μg  S, 0.5  Zn, 0.012 | 3 m | PDD-BI,  ATEC, SAS,  PGI-R  Metabolic parameters | Levels of many vitamins, minerals and biomarkers improved/increased, indicating good compliance and absorption. Statistically significant improvements in metabolic status were numerous, including total sulphate (+17%, *p*=0.001), SAME; (+6%, *p*=0.003), GSH (+17%, *p*<0. 001), GSSG/GSH (-27%, *p*=0.002), nitrotyrosine (-29%, *p*=0.004), ATP (+25%, *p*<0.001), NADH (+28%, *p*<0.001), and NADPH (+30%, *p*=0.001). Most of these metabolic biomarkers improved to normal or near normal levels. The intervention group had significantly greater improvements than the placebo group on the PGI-R (mean change, *p*=0.008) and the subscores for hyperactivity (*p*=0.003), temper tantrums (*p*=0.009), total (*p*=0.02), and receptive language (*p*=0.03). For the other three assessment tools, the difference between the treatment and placebo groups was not statistically significant. The degree of improvement in the mean change in the PGI-R was strongly associated with several biomarkers (adj. r^2^=0.61, *p*<0.001), with baseline levels of biotin and vitamin K being the most significant (*p*<0.05); both biotin and vitamin K are produced by beneficial gut flora | CTI: NCT01225198.  Vit/Supplement formulation is given for a 60 lb child; however, the dosage was adjusted according to the body weight of the participants |
| Kałużna-  Czaplińska,  2011  (21840465) | 20/10 | 4-7 | NA | ASD | Vit B2  Vit B6  Mg | Vit B2, 0.02  Vit B6, 0.5  Mg, 0.2 | 3 m | Dicarboxylic acids, Creatinine | The intervention reduced the level of dicarboxylic acids in the urine of ASD patients | Parents observed improvement in some autistic symptoms, such as the ability to concentrate and ability to make eye contact |
| Kałużna-  Czaplińska,  2011  (21530806) | 30/21 | 4-11 | 27/3 (P)  20/1 (C) | ASD | Intervention group 1:  Folic acid, Vit B6, Vit B12  Intervention group 2:  Vit B6, Vit B12  Control group | Folic acid, 400 μg  Vit B6, 0.2  Vit B12, 1.2 μg | 3 m | Urinary homocysteine | Urinary homocysteine levels were significantly higher in ASD children before the intervention (2.41 (1.10)) compared to both Vit supplementation (1.13 (0.44)) and non-ASD control children (1.33 (0.39)). The intake of vitamins B6 and B12 together with folic acid was found to be more effective in reducing urinary homocysteine levels than the intake of vitamins B6 and B12 alone |  |
| Xia,  2011  (21417812) | 1/0 | 9 | 1/0 | ASD | Intervention:  DMG, Vit B6, Mg | DMG, 0.375  Vit B6, 0.0375  Mg, 0.180 | 5 m | ATEC | The ATEC scores performed by 2 independent raters showed that the changes in total ATEC were from 63 at pretest to 33 at posttest and from 64 at pretest to 30 at posttest, respectively. These changes represented reductions of 47.6% and 53.1%, respectively | Case report |
| Almeida,  2010  (20976769) | 563/0 | 63 (11.4) | 86/187 | MDD | Intervention group:  Folic acid, Vit B6, Vit B12  Placebo group:  NA | Folic acid, 0.002  Vit B6, 0.025  Vit B12, 0.0005 | 1-10.5 y | Onset of DSM-IV major depression, Prevalence of major/minor depression | B vitamins allocation was associated with a lower hazard of MDD compared with placebo (18.4% vs. 23.3%, adjusted hazard ratio=0.48; 95% confidence interval [CI]=0.31-0.76) and a trend toward a lower incidence of major or minor depression at the end of the study compared with placebo (19.1% vs. 27.7%, adjusted odds ratio=0.58; 95%CI=0.31-1.09) | Participants were people who had recently had a stroke or transient ischemic attack |
| Huss,  2010  (20868469) | 810/0 | 5-12  8.6 | 579/231 | ADHD | Intervention group:  PUFA, Mg, Zinc | PUFA, 0.5  Mg, 0.080  Zn, 0.005 | 3 m | SNAP-IV, SDQ | The intervention showed a significant reduction in symptoms of attention deficit and hyperactivity/impulsivity as measured by the SNAP-IV, as well as fewer emotional problems and sleep disturbances | A total of 16 adverse events with a possible causal relationship to the study drug were reported by 14 children (1.7%), and only 5.2% of the children discontinued the study due to tolerability issues |
| Ford,  2010  (20096138) | 300/0 | ≥ 60 | NA | MDD | Intervention group:  B6, B12, folic acid | B12 0.004  B6, 0.025  Folic acid, 0.002 | 12 m | MADRS | Only the design and the rationale of the study are reported | CTI: ACTRN12609000256279 |
| Raz,  2009  (19364294) | 63/0 | 7-13  10.5 (1.5) | 38/25 | ADHD | Intervention group:  LA, α-LA, mineral oil,  α-tocopherol  Placebo group:  Vitamin C | LA, 0.48  α-LA, 0.12  Mineral oil,  0.095  α-tocopherol,  0.005  Vit C, 1 | 7 w | TOVA, CPRS, T-c,  HDL-C,  LDL-C, triglycerides, iron, ferritin,  vit B12, and haemoglobin | In the current study, treatment effects were not significantly different between groups for any measure of ADHD symptoms after 7 weeks of supplementation compared to placebo | Vit C was not the target of the current study |
| Sivrioglu,  2007  (17688987) | 17/0 | 18-55  36.3 (9.5) | 12/5 | SCZ | Intervention group:  PUFA, Vit E, Vit C | PUFA, 0.3  Vit E 400 IU  Vit C 1 | 4 m | BPRS, SANS, SAS, BARS, RBC-MDA, RBC-SOD, GPx, Vit E, Vit C | BPRS, SANS, SAS, and BARS scores were significantly lower at follow-up visits compared to baseline. Superoxide dismutase levels were significantly lower at the end of the study. No significant differences in other laboratory parameters were observed |  |

C: controls; CTI: Clinical Trial Identifier; d: day; F: female; g: grams; h: hours; IU: international units; KEGG: Kyoto Encyclopedia of Genes and Genomes; m: month; M: male; N: number of subjects; NA: not available; P: Patients; PMID: PubMed identifier; w: week; y: years.

Age is presented as mean (SD) or range.

* Doses are given in g/d, %/d, or IU/d unless otherwise specified.

**Phenotype/Disease:**

ADHD: attention deficit hyperactivity disorder; AS: Asperger syndrome; ASD: autism spectrum disorder; BD: Bipolar disorder; FEP: first-episode psychosis; MDD: major depressive disorder; PTSD: post-traumatic stress disorder; psych. ret.: psychomotor retardation; SCZ: schizophrenia.

**Nutraceutical supplements:**

5-HTP: 5-hydroxytryptophan; α-LA: alpha-linoleic-acid; ALCAR: Acetyl-L-carnitine; ALA: α -lipoic acid; Ca: calcium; CoQ: coenzyme Q10; Cr: Chromium; CT: combination therapy; DMG: dimethylglycine; Iron; K: Potassium; LA: linoleic acid; Li: lithium; MC: mixed carotenoids; Mg: magnesium; Mn: Manganese; Mo: Molybdenum; MT: mixed tocopherols; NAC: N-acetylcysteine; PUFA: polyunsaturated fatty acids; S: Sulphur; SAMe: s-adenosylmethionine; Se: selenium; Vit: vitamin; Zn: Zinc.

^§^ *B. bifidum* W23, *B. lactis* W51, *B. lactis* W52, *L. acidophilus* W22, *L. casei* W56, *L. paracasei* W20, *L. plantarum* W62, *L. salivarius* W24, *L. lactis* W19.

^❡^Dosage for a 27 kg child (adjusted up/down by bodyweight): Vit A (6500IU), Vit C (0.5 g), Vit D3 (1000 IU), Vit E (150 IU), Vit K (55 µg), Vit B1 (0.02 g), Vit B2 (0.04 mg), Niacin (0.035 g), Vit B6 (0.04 mg), Folic acid (600 µg), Vit B12 (500 µ), Biotin (225 µg), Pantothenic Acid (0.03g), Iodine (100 µg), Lithium (350 µg), Choline (0.25g), Inositol (0.1 g), Calcium (0.07 g), Magnesium (0.1 g), Zinc (0.015 g), Selenium (40 µg), Manganese (0.001 g), Chromium (70 µg), Molybdenum (100 µg), Potassium (0.05 g) 50, Methylsulfonylmethane (0.5 g), Vit E (0.1 g), CoQ10 (0.05 g), N-acetyl-cysteine (0.045 g), Acetyl-L-carnitine (0.2 g), Vanadium (25 µg), Boron (250 µg). Treatment began with a special vitamin/mineral supplement, and additional treatments were added sequentially, including essential fatty acids, Epsom salt baths, carnitine, digestive enzymes, and a healthy gluten-free, casein-free, soy-free (HGCSF) diet.

**Measures:**

6-GSI: 6-item Gastrointestinal Severity Index; ABC: Aberrant Behaviour Checklist; ADOS: Autism Diagnostic Observation Schedule; ATEC: Autism Treatment Evaluation Checklist; ATP: adenosine-5’-triphosphate; BARS: Barnes Akathisia Rating Scale; BDI-II: Beck Depression Inventory II; BDRS: Bipolar depression rating scale; BMI: Body Mass Index; BPRS: Brief Psychiatric Rating Scale; BPRS-E: BPRS Expanded Version; Ca: calcium; CARS-2: Childhood Autism Rating Scale 2; CDSS: Calgary Depression Scale for Schizophrenia; CGI: Clinical Global Impression; CGI-BP: CGI for use in BD; CPRS: Conner’s Parent Rating Scale; DSM-IV: Diagnostic and Statiscal Manual of Mental Disorders; EQ-5D-5L: health-related quality of life developed by the EuroQol Group; GAD-7: Generalized Anxiety Disorder 7 item scale; GAF: Global Assessment of Functioning; GA^2^LEN-FFQ: Global Allergy and Asthma European Network food frequency questionnaire; GLQI: gastrointestinal quality of life questionnaire; GPx: glutathione peroxidase; GSH: reduced glutathione; GSI: Global Severity Index; GSSG: oxidized glutathione; HDL-C: high-density lipoprotein cholesterol; HDRS: Hamilton Depression Rating Scale; IDS30-SR: Inventory of Depressive Symptomatology; LDL-C: low-density lipoprotein cholesterol; LIFE-RIFT: Longitudinal Interval Follow-up Evaluation – Range of Impairment Functioning Tool; MADRS: Montgomery-Asberg Depression Rating Scale; Mg: magnesium; MSS: Mania Self Rating Scale; NADH: reduced nicotinamide adenine dinucleotide; NADPH: reduced nicotinamide adenine dinucleotide phosphate; PANSS: Positive and Negative Syndrome Scale; PDD-BI: Pervasive Developmental Disorders Behaviour Inventory; PGI: Patient global impression; PGI-2: Parent Global Impressions—Revised-2; PGI-R: Parent Global Impressions—Revised; PHQ-9: Patient Health Questionnaire; PSDI: Positive Symptom Distress Index; PST: Positive Symptom Total; Q-LES-Q (sf): Quality of life enjoyment and satisfaction questionnaire, short form; RBC-MDA: red blood cells – malondialdehyde; RBC-PUFA: red blood cells – polyunsaturated fatty acids; RBC-SOD: red blood cells – superoxide dismutase; RIAS: Reynolds Intellectual Assessment Scales; SANS: Scale for the Assessment of Negative Symptoms; SAS: Simpson Angus Scale; SAS-Pro: Severity of Autism Scale; SCL-90: Symptom Checklist-90-Revised; SDQ: Strengths and Difficulties Questionnaire; SNAP-IV: Swanson, Nolan And Pelham rating scale for the assessment of attention deficit, hyperactivity/impulsivity; SOFAS: social and occupational functioning assessment scale; SQUASH: Short questionnaire to assess health enhancing physical activity; SRS: Social Responsiveness Scale; SSP: Short Sensory Profile; Tc: total cholesterol; TFEQ-R18: Three Factor Eating Questionnaire Revised; TOVA: Test of Variables of Attention; VABS-II: Vineland Adaptive Behaviour Scale II; Vit: vitamin; YMRS: Young Mania Rating Scale; Zn: zinc.
