# Supplementary material for: Clinical outcomes of mitochondrial‐enhancing nutraceutical supplementation in psychiatric disorders: A systematic review
Source: Gen Psychiatr. 2026 Jun 1;39(3):e70023. doi: 10.1002/gps3.70023 (PMC13239229; doi:10.1002/gps3.70023)
Supplement: Supplementary file 3 — Figure S2 [file GPS3-39-e70023-s003.pdf]

Supplementary Figure 2. Cochrane Risk of Bias (ROB) tool for randomized trials version 2 (RoB 2).

The figures were generated using the “robvis visualization tool”, a web application designed to visualize the ROB assessments performed as part of the systematic review. In this color-coded ranking system, green represents a low ROB if the study is judged to have a low ROB for all domains for that study; yellow represents some concerns if the study is judged to have some concerns for at least one domain for that outcome, but not a high ROB for any domain; and red represents a high ROB if the study is judged to have a high ROB for at least one domain for that outcome, or if the study is judged to have some concerns for multiple domains that substantially reduce confidence in the outcome. Multiple domains are examined: D1, bias due to the randomization process; D2, bias due to deviations from the intended intervention; D3, bias due to missing outcome data; D4, bias in the measurement of the outcome; D5, bias in the selection of the reported outcome.

ALA: alpha-lipoic acid; CoQ10: coenzyme; NAC: N-acetyl cysteine; Q10; Vit: vitamin.

CoQ10 (Study)	D1	D2	D3	D4	D5	Overall
Maguire A, 2021	+	+	+	+	-	-
Jahangard L, 2019	+	+	X	+	-	-
Mousavinejad E, 2018	-	+	+	+	-	-

ALA (Study)	D1	D2	D3	D4	D5	Overall
De Lima D, 2023	+	+	+	+	-	-
Mishra A, 2022	+	+	+	+	-	-
Vidovic B, 2014	X	+	+	+	-	X
Emsley R, 2014	+	-	+	+	-	-

Magnesium (Study)	D1	D2	D3	D4	D5	Overall
Hemamy M, 2021	+	+	+	+	-	-
Afsharfar M, 2021	+	+	+	+	-	-
Rajizadeh A, 2017	+	+	+	+	-	-

Vit B6 (Study)	D1	D2	D3	D4	D5	Overall
Rizzo R, 2022	-	X	+	X	-	X
Badrfam R, 2021	+	+	+	+	-	-
Kaluzna-Czaplinska J, 2011	X	-	+	+	-	X

Judgement: X High; - Some concerns; + Low

Vit D3 (Study)	D1	D2	D3	D4	D5	Overall
Rahman S, 2023	+	+	+	+	+	+
Yang J, 2023	X	+	+	+	-	X
Vyas C, 2023	+	+	+	+	-	-
Samadi M, 2022	+	+	+	+	-	-
Kumar S, 2022	+	+	+	+	-	-
Mohammadzadeh N, 2022	+	X	+	+	-	X
Amini S, 2022	+	-	+	+	-	-
Gaughran D, 2021	+	+	+	+	+	+
Okereke O, 2021	+	+	+	+	+	+
Libuda L, 2020	+	X	+	+	-	X
Alghamdi S, 2020	X	-	X	-	-	X
Kerley C, 2020	-	-	+	+	-	-
Kaviani M, 2020	+	+	+	+	-	-
Hansen J, 2019	+	X	X	+	-	X
Ghaderi A, 2019	+	-	+	+	-	-
Mazahery H, 2019	+	+	X	+	-	X
Mousa A, 2018	+	X	+	+	-	X
Mohammadpour N, 2018	+	X	+	X	-	X
Krivoy A, 2017	+	+	+	+	-	-
Kerley C, 2017	+	+	+	X	-	X
Marsh W, 2017	+	X	X	+	-	X
Feng J, 2017	X	X	+	X	-	X
Vaziri F, 2016	-	X	+	X	-	X
Wang Y, 2016	+	+	+	+	-	-
Sikoglu E, 2015	X	X	+	X	-	X
Snoeijen-Schouwenaars F, 2015	X	+	+	+	-	X
Kilpinen-Loisa P, 2009	X	+	+	+	-	X

Folic Acid	D1	D2	D3	D4	D5	Overall
Hosseini S, 2023	+	-	+	+	-	-
Surman C, 2019	+	+	+	+	-	-
Roffman J, 2018	+	+	+	+	+	+
Sun C, 2016	X	+	X	X	-	X
Bedson E, 2014	+	+	+	+	-	-
Loria-Kohen V, 2013	+	+	X	+	-	X
Hill M, 2011	+	+	+	+	-	-

Vit B12	D1	D2	D3	D4	D5	Overall
Roffman J, 2013	+	+	X	+	+	X
Christensen H, 2011	+	+	+	+	+	+

Vit A	D1	D2	D3	D4	D5	Overall
Lai X, 2021	-	+	+	X	-	X
Guo M, 2018	X	+	+	X	-	X
Liu J, 2017	-	+	+	X	-	X

Vit C	D1	D2	D3	D4	D5	Overall
Zhang J, 2023	X	-	+	+	-	X
Wang Y, 2013	+	+	+	+	+	+
Raz R, 2009	+	+	+	+	-	-

Vit B3	D1	D2	D3	D4	D5	Overall
Raison C, 2023	-	+	+	+	-	-

Vit B7	D1	D2	D3	D4	D5	Overall
Reininghaus E, 2020	X	+	+	+	-	X

Multiple	D1	D2	D3	D4	D5	Overall
Russell S, 2023	+	+	+	+	-	-
Weggen J, 2021	+	X	+	+	-	X
Van der Burg K, 2020	X	X	X	+	-	X
Bot M, 2019	+	+	+	+	-	-
Allot K, 2019	+	+	+	+	+	+
Adams J, 2018	-	+	X	+	+	X
Kaluzna-Czaplinska J, 2017	X	X	+	+	-	X
Adams J, 2011	+	+	X	+	+	X
Almeida O, 2010	+	+	X	+	+	X

Study

Vit E	D1	D2	D3	D4	D5	Overall
Pawelzyk T, 2021	+	X	+	+	-	X
Szeszko P, 2021	-	X	+	+	-	X
Robinson D, 2019	+	-	+	+	-	-
Qiao Y, 2018	+	-	+	+	-	-
Kean J, 2017	-	X	+	+	-	X
Chhetry B, 2016	-	+	+	+	-	-
Pawelzyk T, 2016	+	+	+	+	-	-
Boskovic M, 2016	+	+	+	+	-	-
Pawelzyk T, 2015	+	+	+	+	-	-
Amminger G, 2015	X	+	+	+	-	X
Smesny S, 2014	-	-	+	+	-	-
Bentsen H, 2013	+	+	+	+	-	-
Amminger G, 2013	-	-	+	+	-	-
Meyer B, 2013	+	+	+	+	-	-
Germano M, 2007	X	+	+	+	-	X

Study

NAC	D1	D2	D3	D4	D5	Overall
Kanaan R, 2023	+	+	+	+	-	-
Neill E, 2022	+	-	+	+	-	-
Bortolasci C, 2021	-	X	+	+	-	X
Ashton M, 2020	-	+	+	+	-	-
Mullier E, 2019	+	X	+	+	-	X
Yang Y, 2019	+	+	X	+	-	X
Yang Y, 2018	+	+	+	+	-	-
Breier A, 2018	+	X	+	+	-	X
Conus P, 2018	+	-	+	+	-	-
Sepehrmanesh Z, 2018	-	+	+	+	-	-
Rapado-Castro M, 2017	+	-	+	+	-	-
Dean O, 2017	+	+	+	+	+	+
Wink L, 2016	+	-	+	+	-	-
Ghanizadeh A, 2013	+	X	+	+	-	X
Hardan A, 2012	+	X	+	+	-	X
Berk M, 2008	+	X	+	+	-	X
